# Arpeggio: harmonic compression of ChIP-seq data reveals protein-chromatin interaction signatures

**DOI:** 10.1093/nar/gkt627

**Published:** 2013-07-19

**Authors:** Kelly Patrick Stanton, Fabio Parisi, Francesco Strino, Neta Rabin, Patrik Asp, Yuval Kluger

**Affiliations:** ^1^Department of Pathology, Yale University School of Medicine, 333 Cedar Street, New Haven, CT 06520, USA, ^2^Department of Exact Sciences, Afeka - Tel-Aviv Academic College of Engineering, Tel-Aviv 69107, Israel, ^3^Department Of Liver Transplant, Montefiore Medical Center, Albert Einstein College of Medicine, Bronx, NY 10467, USA and ^4^NYU Center for Health Informatics and Bioinformatics, New York University Langone Medical Center, 227 East 30th Street, New York, NY 10016, USA

## Abstract

Researchers generating new genome-wide data in an exploratory sequencing study can gain biological insights by comparing their data with well-annotated data sets possessing similar genomic patterns. Data compression techniques are needed for efficient comparisons of a new genomic experiment with large repositories of publicly available profiles. Furthermore, data representations that allow comparisons of genomic signals from different platforms and across species enhance our ability to leverage these large repositories. Here, we present a signal processing approach that characterizes protein–chromatin interaction patterns at length scales of several kilobases. This allows us to efficiently compare numerous chromatin-immunoprecipitation sequencing (ChIP-seq) data sets consisting of many types of DNA-binding proteins collected from a variety of cells, conditions and organisms. Importantly, these interaction patterns broadly reflect the biological properties of the binding events. To generate these profiles, termed Arpeggio profiles, we applied harmonic deconvolution techniques to the autocorrelation profiles of the ChIP-seq signals. We used 806 publicly available ChIP-seq experiments and showed that Arpeggio profiles with similar spectral densities shared biological properties. Arpeggio profiles of ChIP-seq data sets revealed characteristics that are not easily detected by standard peak finders. They also allowed us to relate sequencing data sets from different genomes, experimental platforms and protocols. Arpeggio is freely available at http://sourceforge.net/p/arpeggio/wiki/Home/.

## INTRODUCTION

The advent of automation and use of high-throughput sequencing techniques has brought a remarkable increase in the rate at which biological data sets are accumulated. Public repositories, such as the Sequence Read Archive (SRA, http://www.ncbi.nlm.nih.gov/sra), and The Cancer Genome Atlas (http://cancergenome.nih.gov/) already store thousands of genome-wide data sets from a variety of cells and biological conditions. It is plausible that similarities of genomic profiles from different experiments (e.g. binding profiles of transcription factors or transcriptomes of unique samples) are due to similar biological mechanisms, and theoretically it is therefore possible to use existing repositories to explore uncharted relationships between a variety of genomic signals in various biological systems and conditions.

For instance, it would be possible to gain biological insights relevant to a new genome-wide study by using exploratory unsupervised learning approaches that link newly generated data with well-annotated data sets possessing similar genomic patterns. Integration of new data with existing repositories in standard pipelines for sequence analysis is computationally challenging owing to the high dimensionality of each genomic profile and the massive storage size of these databases. Data compression techniques are needed for addressing these issues and can be incorporated into these pipelines to provide efficient comparisons of new genomic profiles to the large volume of publicly available profiles. Furthermore, data compression techniques that allow comparison of genomic signals from different platforms and across species would enhance our ability to use large existing repositories.

Compressing and organizing large sequencing archives involves characterization of each experiment in terms of its genomic features, such as a list of peaks representing events along the genome, application of dissimilarity measures to determine pairwise affinities between the feature vectors of each pair of experiments (e.g. overlap between two lists of peaks), clustering, dimensional reduction, annotation and visualization of the collection of these feature vectors. We usually assume that the underlying cellular mechanisms captured by a pair of dissimilar feature vectors, such as lists of binding sites of two different DNA-binding proteins, are different. However, data analysis of each type of sequencing experiment can be done in numerous ways that affect the affinity between pairs of unique data sets. The most obvious factors that influence affinity include the preferred choice of feature space and dissimilarity measures used. In most sequencing analyses, practitioners tend to use standard feature spaces and common dissimilarity measures. Specifically, in RNA-seq analysis, the feature space of an experiment comprises counts of reads or sequenced fragments (e.g. RPKMs or FPKMs) of all genes, and the similarity between two transcriptomes is characterized by standard correlation measures (1,2); in DNA-seq analysis of cancer samples, the standard feature space includes point mutations, indels, copy number alterations and translocations, and similarities are evaluated using basic association measures to determine prevalence (3); in chromatin-immunoprecipitation sequencing (ChIP-seq) experiments, binding events are determined by peak detectors, and similarities between two experiments are typically evaluated simply by the number or fraction of overlapping peaks (4–6).

ChIP-seq in particular has been widely used to unravel transcriptional and epigenetic regulatory programs that ultimately determine the biological phenotype. Thousands of ChIP-seq experiments have already been collected by large community-wide efforts such as the ENCODE project (7,8), pilot initiatives (4–6,9), and smaller projects (10–45). Application of computational approaches for interrogating the genome-wide interactions between chromatin and proteins by high-throughput short read sequencing of genomic DNA from ChIP-seq experiments can reveal certain aspects of the underlying biology (46–48).

In the present study, we use deconvolution to extract the biological component that is indicative of distinctive protein–chromatin interaction configurations from the autocorrelation profiles of ChIP-seq signals. We explored the space of the Fourier transform of the autocorrelation profiles (spectral densities of the read coverage distributions) using machine-learning approaches to characterize protein–chromatin interaction patterns at intermediate length scales of several kilobases and showed its utility in the organization of large repositories of ChIP-seq data. These characteristic spectral density profiles allowed us to efficiently compare a large number of ChIP-seq data sets consisting of transcription factors, epigenetic marks and other types of chromatin interacting proteins collected from a variety of cell types, conditions and organisms. Moreover, the deconvolved autocorrelation functions, which we term Arpeggio profiles, reflect the biological nature of protein–chromatin interactions, such as events that are locally isolated, coordinated events or dynamically flexible events.

We used 806 publicly available ChIP-seq experiments from several unrelated studies (4,5,8–45) and showed that Arpeggio profiles with similar spectral densities shared biological properties. Arpeggio profiles can be modeled using a small number of parameters and thus are mappable to a low-dimensional space that captures biological aspects of the interaction between proteins and chromatin. This representation facilitates efficient indexing of databases and application of supervised, unsupervised and inference methods to large repositories of sequencing data comprising different genomes, experimental platforms and protocols. We also show that our approach can be used to derive experimental and biologically meaningful quantities, such as fragment length distributions, as well as the expected nucleosome spacing. Our results suggest that harmonic analysis of ChIP-seq data unravels signatures that are not easily captured by standard computational means. Analogously to cataloging cerebral activity with Electroencephalography (EEG), Arpeggio analysis efficiently locates a new sample in the map of differing protein–chromatin interaction states.

## MATERIALS AND METHODS

### Data sets and preprocessing

#### Data

We analyzed 806 public ChIP-seq experiments from data sets obtained from the SRA (http://www.ncbi.nlm.nih.gov/sra, Supplementary Table S1). The analyzed proteins included transcription factors or histone modifications from human, mouse or fruit fly (Supplementary Figure S1). In all experiments, chromatin was cross-linked and fragmented using either sonication or MNase digestion followed by immunoprecipitation using specific antibodies (Supplementary Table S2). The ChIP-seq protocols used in all these studies are transcribed verbatim and provided in Supplementary Table S3. Controls consist of high-throughput sequencing of immunoglobulin G immunoprecipitation (IP) or total DNA input.

#### Preprocessing

With the exception of sequenced reads from the study by Barski *et al.* (5), whose genome-wide alignment is provided by the authors, all sequenced reads were mapped to their corresponding reference genomes using the Bowtie aligner (49) with parameters ‘-n2 -k1 -m1 –best –strata’, corresponding to reporting unique alignments for each read with at most two mismatches.

### Autocorrelation and cross-correlation of ChIP-seq profiles

#### Autocorrelation

Given a short read sequencing sample consisting of a set *R* of *N* aligned reads on the same strand 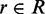, where *r* indicates the 5′ end of an aligned read, we defined the count of all pairs of reads separated by a fixed distance of τ nucleotides as:
(1)
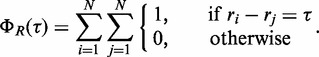


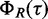
 is equivalent to the autocorrelation of the empirical read coverage depth *D*(*t*) at each position *t* along the genome,
(2)
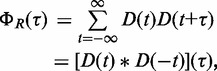

where 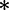 is the convolution operator.

We note that proper normalization of the read count data is ambiguous (50,51); therefore, rather than the statistical definition of autocorrelation, which is mean centered and normalized by variance, we use the digital signal processing (DSP) definition of autocorrelation. The latter does not require prior knowledge of the distribution shape. To minimize the impact of PCR artifacts, duplicated reads were only considered once.

#### Cross-correlation

Given a short read sequencing sample consisting of a set *R* of aligned reads 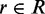, where 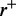 indicates the starting position of an aligned read on the positive strand, and 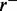 indicates the starting position of an aligned read on the negative strand, we defined the aggregated cross distance between reads on opposing strands as:
(3)
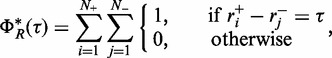

where τ is the offset in nucleotides and 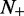 and 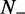 are the number of reads on the positive and negative strand, respectively. This is equivalent to the cross-correlation of the empirical read coverage depth on the positive strand 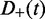 with the empirical read coverage depth on the negative strand 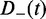 at each position *t* along the genome,
(4)
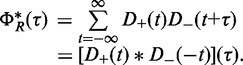



As in the case of autocorrelation, duplicated reads were only considered once.

#### Principal component analysis

Principal Component Analysis (PCA) was applied to the collection of Fourier transforms of the Arpeggio profiles. Only the real part of the transform should exist, and to avoid numerical errors, the negligible imaginary part was discarded. To avoid bias due to noise affecting length-scales below 40 bp, we applied a low-pass filter to the Fourier transform.

#### Davies–Bouldin index

The data in our data set were annotated by several class variables (e.g. Antibody target, cell line, cellular mechanism, organism, study ID), each consisting of multiple class labels (e.g. for Antibody target: histone 3 Lysine 27 tri-methylation (H3K27me3), H3K27me2, E2F4, etc.). For each class variable, a sample was assigned one and only one class label. We assigned samples to clusters based on class label and computed the Davies–Bouldin index (52) as:
(5)
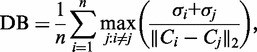

where 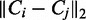 was the Euclidean distance between the two centroids *C_i_* and *C_j_*, computed using the six leading principal components, *n* was the number of clusters, and 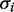 and 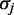 were the mean distances of the points in the *i*-th and *j*-th clusters from the cluster centroids *C_i_* and *C_j_*. *P*-values were computed via bootstrapping (*n* = 1000).

#### Class label aggregation for different data representations

For each sample, we tested whether proximity in a given data representation is indicative of similar class label assignments. We selected a class variable (e.g. Antibody target, orgnanism, cellular mechanism) and for a given sample, we constructed a binary class that designates as positives other samples with the same class label and designates all other samples as negatives. Using this binary class label, we computed the Area Under the Receiver Operator Characteristic Curve (AUC) for the pairwise distances of the given sample with all other samples (53). As the fraction of positive samples in close proximity to the given sample increases, so does the AUC.

To avoid sampling bias, we considered only one replicate at random for each group of replicated samples. Once a sample that determines the positive class label is chosen, its replicates are excluded. This AUC calculation was repeated 100 times (different replicate combinations chosen at random) for each sample, and we computed the sample-specific expected value for the AUC as well as its standard deviation. For all samples with the same class label, we computed the average over these expected AUCs and the average standard deviation for each class label, similarly to standard approaches (54). Finally, for each class variable, we reported the medians across all class labels (e.g. human, mouse and fly for the organism class variable) for both the average expected AUC and its average standard deviation. We report the median rather than the mean because it is more robust to outliers and is a better estimator of expected value for arbitrary distributions.

### Supervised analyses

K-nearest neighbors classifiers (k = 1) were trained to identify the class labels in the class variables of interest. To avoid sampling bias, we considered only one replicate at random for each group of replicated samples. For each class variable, we kept one sample for testing, and performance was computed using the balanced accuracy (accuracy for each class label, averaged over all labels) (55). This procedure was repeated for all samples, and *P*-values were computed via bootstrapping (*n* = 100).

### Statistical analyses and software

All analyses were performed using our Java-based software package and the R statistical software (56). Our Arpeggio software can be used to download data from the SRA, map reads to reference genomes, compute autocorrelation and Arpeggio profiles. An R script is also available to generate and plot Arpeggio profiles. The Arpeggio software suite is freely available at http://sourceforge.net/p/arpeggio/wiki/Home/ together with a detailed tutorial.

## RESULTS

### Spectral density of ChIP-seq signals

To enhance our understanding of a new ChIP-seq sample, it is often beneficial to relate it to relevant ChIP-seq experiments in public repositories. Here, we relate experiments based on their signal proximity, and therefore an appropriate distance metric is needed.

A naïve comparison of ChIP-seq experiments can be done by measuring the distance between their coverage depth graphs. The genome consists of a large number of genomic positions (∼3 billion for the human genome) from which one can theoretically sample reads. This pairwise comparison is not efficient for large data sets, and the dimension may be too large to identify neighbors (57). In addition, at single nucleotide resolution, meaningful patterns can be obscured by noise. Standard comparisons between pairs of ChIP-seq experiments are commonly done by evaluating the overlap between peaks detected in these experiments (4,5,8). Intuitively, the union of peaks from a large data sets, which is needed to generate pairwise distances, produces many genomic intervals and is thus high dimensional. To quickly relate a given ChIP-seq sample to relevant experiments, we sought to design a data representation that captures the underlying biology, is easy to compute, can be expressed by a relatively small number of dimensions and finally is robust to suboptimal read coverage.

We leveraged two important characteristics of ChIP-seq data to create this low-dimensional representation. First, reads are localized in islands surrounding the interacting proteins (e.g. factors, histones or polymerases) that were targeted by the antibody (58). We therefore examined the system at intermediate genomic length scales. Specifically, we consider the autocorrelation function of the read coverage depth. This function captures recurrent events along the genome and aggregates this information to signatures of read co-occurrence at specific length scales or lags (Supplementary Figures S2–S4). As a consequence of the localized nature of ChIP-seq, the autocorrelation function exhibits regular non-random interactions within a relatively small offset 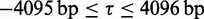 after which it is uninformative. Second, read count data are stochastic, and typically undersampled, resulting in spikey noise that obscures signals occurring at nucleotide resolution (Supplementary Figure S5). At long length scales far beyond the length of protein–chromatin interaction islands and also at short length scales on the order of nucleotides, the signal is dominated by noise, and therefore we set out to capture the signal at intermediate length scales where the signal-to-noise ratio is the highest. To this aim, we applied a Fast Fourier Transform to the autocorrelation function for all lags 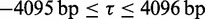 (see ‘Materials and Methods’ section) resulting in the spectral density of the empirical read coverage distribution, 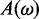, as a function of the resolution ω.

The autocorrelation is restricted to a fixed range (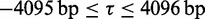), and thus the associated spectral density, is fast and easy to compute, and it is constructed as the histogram of pairwise distances between all *N* reads (see ‘Materials and Methods’ section). This approach enables a large coverage for each lag (τ) in the autocorrelation function.

### Extraction of the IP signal using deconvolution

The observed autocorrelation signal consists of a biological component relevant to the ChIP-seq experiment modulated on a component capturing irrelevant properties such as DNA accessibility and experimental bias. We therefore designed an approach to deconvolve the component of the signal that captures the biological aspects of the experiment.

We formulated the problem using a DSP approach. The ChIP-independent properties of the signal are denoted **technical variability**, *X*(*t*), and arise from technical biases and the stochastic nature of read count data. We denote by *Z*(*t*) the **true IP signal**, which reflects effects associated with experiment-specific components, e.g. antibody precipitation, cross-linking of chromatin to other proteins in the same complex. We therefore model the **measured ChIP signal** *Y*(*t*) as the convolution of the true IP signal with the technical variability, 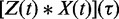. For brevity, we will omit 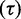 in the equations when clear from the context.

**Figure 1.**
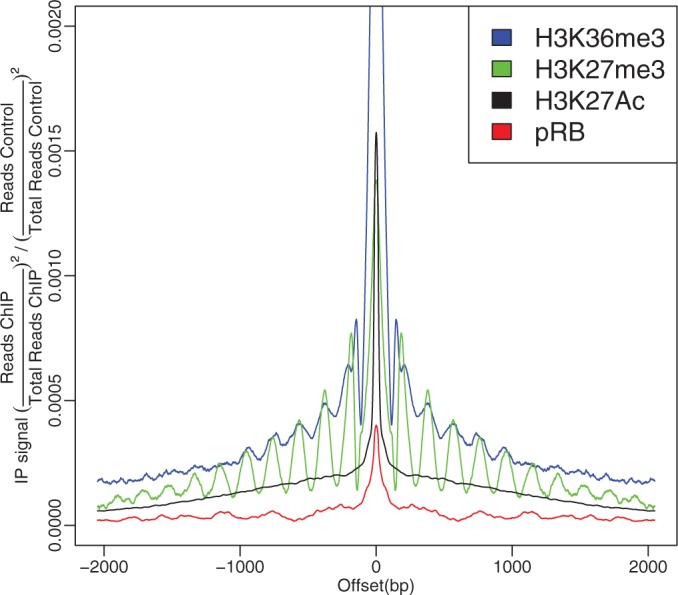
Examples of the deconvolved Arpeggio profiles for histone marks H3K27me3, Histone 3 lysine 36 tri-methylation (H3K36me3), Histone 3 lysine 27 acetylation (H3K27Ac) and for retinoblastoma (pRB). The H3K27me3 and H3K36me3 profiles show periodicity of 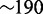 bp consistent with previous reports of nucleosome frequency (60). The value representing the total read count difference between ChIP and control at 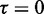 was removed, and smoothing was applied to aid in visualization.

In the DSP framework *X*(*t*) is the input, *Z*(*t*) is the finite impulse response function associated with the specific biological signal, and *Y*(*t*) is the observed output. To recover the specific finite impulse response and remove ChIP-independent components, we used harmonic analysis techniques that are commonly used in engineering disciplines (59). In harmonic analysis, signals are represented as the sum of characteristic harmonic components (i.e. sinusoidal functions, each with a specific period and phase). In this formulation, if the technical variability *X*(*t*) is known, then applying the convolution theorem, it is possible to recover the true IP signal *Z*(*t*) from the measured signal *Y*(*t*); consequently, if the autocorrelation 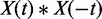 is known, then it is possible to recover the autocorrelation of the true IP signal 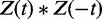,
(6)
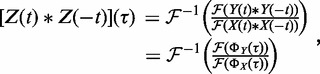

where 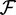 is the Fourier transform operator, 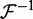 is its inverse, 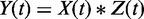, and 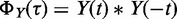 and 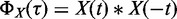 are the autocorrelations of the ChIP-seq signal *Y* and of the control *X*, respectively. We named the recovered autocorrelation of the true IP signal 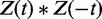 the **Arpeggio** profile (Figure 1).

**Figure 2.**
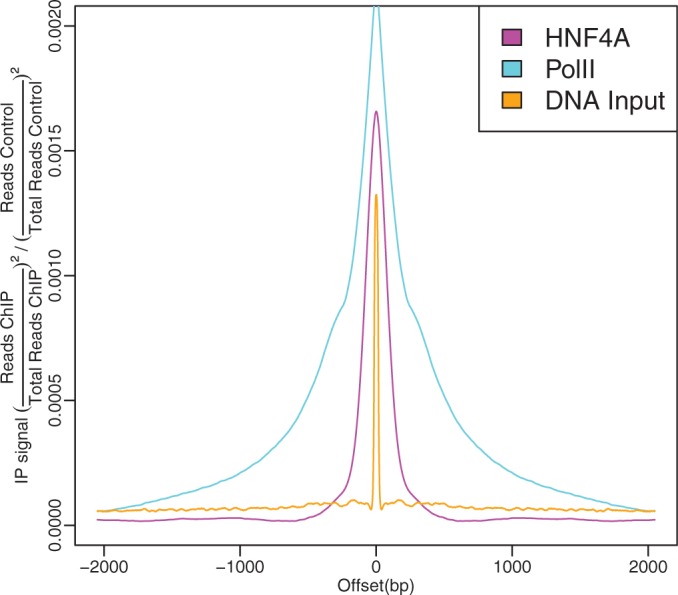
Examples of the deconvolved Arpeggio profiles for a transcription factor, PolII and a total DNA input sample. The transcription factor HNF4A shows a strong spike followed by a steep decay indicating isolated binding, and PolII shows an isolated pulse followed by gradual decay. The isolated pulse for total DNA input indicates that sequenced reads provide no additional information beyond technical variability 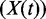. The DNA input Arpeggio was constructed using the sample’s best matched control. As in the previous figure, the read count difference between the sample and the matched control at 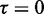 was removed, and smoothing was applied to aid in visualization.

In studies where multiple controls are available or no controls are available, we matched to each ChIP-seq experiment the control that is closest in terms of its spectral properties. We applied the same control matching procedure to the rest of the samples and found that most samples matched controls done in the same study or cell line. This control matching procedure is a conservative approach in which we try to identify the parts of the *Y* spectra that are significantly distinct from the *X* spectra and thus increases specificity. In the extreme case, where the autocorrelation of the measured ChIP signal 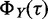 and of the technical variability 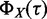 have proportional spectral densities, i.e. they are linearly correlated, their ratio will be constant and their deconvolution is an impulse, indicating that there is no true IP signal (Figure 2).

We matched controls to experiments from the pool of controls in our data set by first matching the organism and DNA-shearing technique and selected the control with the highest correlation (Pearson’s 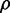) to the ChIP experiment in the base resolution domain (frequency domain, i.e. after applying the Fourier transform). We also require that *ρ* > 0.85. We note that this leads to the highest specificity in the context of our spectral analysis. We recall that correlation in the resolution domain does not imply correlation in the genomic co-ordinates domain. The value of the Arpeggio profile at 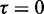 reflects differences in read count between experiment and control. From the values of 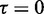 recorded in our data set, we concluded that no experiment control pair had a perfectly matching read count. In general, this did not significantly affect our ability to recover the autocorrelation of the true IP signal.

However, for 31 samples of 806 in our data set, we could not identify any matching control. Of the remaining 775, there was also a small fraction of experiments for which the resulting Arpeggio profile exhibited several artifacts, suggesting poorly matched controls. In particular, when the autocorrelation of the control had a different decay rate than that of the experiment, we observed dips around 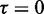 or gradual rises or falls as opposed to leveling out at greater values of 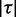 (Supplementary Figure S6). This difference in decay rate is likely due to technical variability dependent on read counts. However, there may be biological components to it as well, e.g. if a polymerase binds and then moves along the genome, it is reasonable to believe that the reads will be more spread than a transcription factor that binds to a single location.

It would have been desirable to extract the true IP signal *Z*(*t*) from 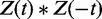 directly by square root in the resolution domain. However, in general, 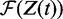 is not entirely positive or real; thus, there is no unique solution for *Z*(*t*) because the phase information is lost in the autocorrelation operation. In practice, the Arpeggio profiles 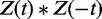 are sufficient for the purpose of comparing ChIP-seq experiments and can also be used to examine the spectral density as a function of base resolution.

### Recovering the length distribution of ChIP-seq fragments

The fragment length distribution is an important parameter for algorithms that seeks to identify binding event locations from short read experiments such as ChIP-seq peak callers (61). If paired-end reads are available, they can be used to recover the fragment length distribution empirically; however, many experiments in current repositories were not done using paired-end reads. We note that for a given number of nucleotides sequenced, the single end approach represents twice as many fragments and thus is more sensitive than the paired-end approach. The latter, however, has an advantage in mapability in repetitive regions.

Previous studies have used cross-correlation to infer the average fragment length of singled-end short read ChIP-seq data (62–64). As known, when considering reads from opposing strands, some of the measured distances reflect fragment lengths (62–66).

We modeled the probability associated with sampling a fragment starting at any given position on the positive strand of the genome as 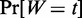. If the sequenced read from this fragment happens to map to the positive strand, it will start at the same genomic position *t*, giving the same probability distribution for the reads 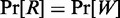. If, however, the sequenced read maps to the negative strand, then its starting position is *F* = *f*, the fragment length, nucleotides downstream from the starting position of the fragment *W* = *t*. Thus, given the probability of sampling a read on the positive strand 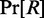, there is an equivalent probability of sampling a read on the negative strand 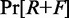, where *F* is a random variable representing the fragment length.

We show that harmonic deconvolution can be used to determine not only the average fragment length but also the full empirical fragment length distribution. This is done by deconvolving the cross-correlation (see ‘Materials and Methods’ section) between the start of reads aligning to opposite strands, which we denoted as 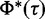, from the autocorrelation of reads aligning to the same strand for the same experiment, 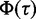. We recall that the probability distribution of the sum of two random variables is equivalent to the convolution of their individual probability distributions, and thus
(7)
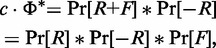

where *c* is a normalization constant such that 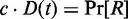. Similarly to Equation (6), we deconvolve the fragment length distribution:
(8)
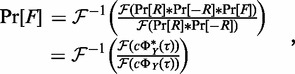



**Figure 3.**
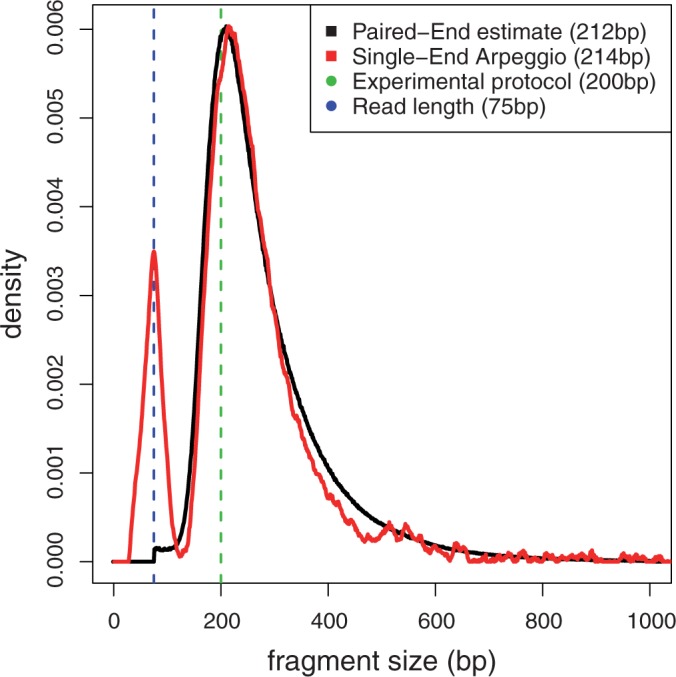
Comparison between paired-end fragment length distribution and Arpeggio fragment length distribution. The Arpeggio fragment length distribution was estimated from only one read of each read pair. The reported fragment length from the experimental protocol, together with the sequenced read length, is shown as dashed lines. The arpeggio fragment length distribution shows an additional spike at the read length, which has been previously observed in opposing strand cross-correlation (63).

We found that the reported fragment size from the different studies included in our data set matched the fragment size inferred using our deconvolution approach (Supplementary Figure S7 and Supplementary Table S2). Moreover, our deconvolution approach, using only one read from each read pair of a paired end experiment, produced an estimate of the fragment length distribution that closely matched to the length distribution of the paired-end fragments. (Figure 3, see Supplementary Note A).

### Arpeggio captures the biology of protein–chromatin interaction

#### The space of Arpeggio spectral densities is low dimensional

To facilitate organization of large sequencing archives, in particular, during search operations for complex queries, or computationally intensive machine-learning tasks, it is desirable to represent the samples using a small number of features. Compared with the size of the genome, spectral densities of Arpeggio profiles, described by only 8192 elements, are already relatively small. We investigated whether the space comprising all spectral densities in our database could be further reduced using Multi-Dimensional Scaling (MDS), such as PCA. We found that six principal components were sufficient to capture 85% of the variability present in our collection of spectral densities.

Although more advanced techniques (67) may result in a better compression, i.e. fewer dimensions, we decided to use PCA, which is readily available and familiar for many practitioners. We used the leading six spectral density principal components to organize hundreds of ChIP-seq experiments and aid annotation of novel samples. We termed this representation **Arpeggio MDS**.

#### Use of Arpeggio MDS coordinates for classification and clustering

Classification and clustering are affected by choice of data representation and dissimilarity measures. Here, we use the Davies–Bouldin index (52) to assess discernability between clusters and inferred class labels for each class variable using a k-nearest neighbor classification.

First, for each class variables, e.g. antibody target, cellular mechanism, or organism, we considered class labels as cluster indices and computed the Davies–Bouldin index (see ‘Materials and Methods’ section). Our analysis showed significantly small Davies–Bouldin indices for all class variables (Supplementary Table S4). This indicates that the organization of the data based on Arpeggio MDS coordinates has a structure amenable for a variety of machine-learning approaches. The Davies–Bouldin index might have been affected by sampling bias of proteins analyzed within each organism group, skewing the index for the organism class variable.

**Figure 4.**
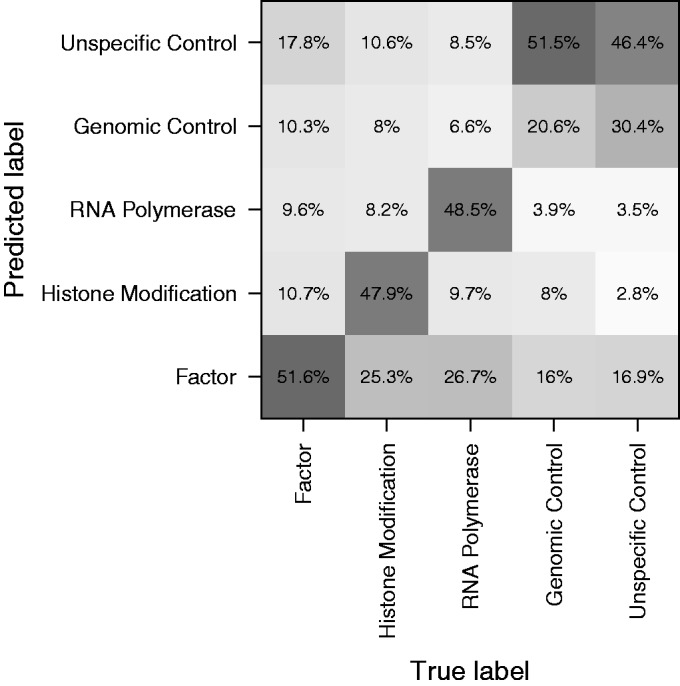
Confusion matrix of predicting functional annotations from the Arpeggio profiles using a k-nearest neighbor classifier with k = 1. The values along each column of the matrix represent how the instances in the actual class were assigned to the predicted class. The diagonal elements indicate sensitivity. Darker colors indicate higher classification frequencies. Most misclassifications occurred between controls.

Second, for each class variable, we trained a k-nearest neighbor classifier (k = 1) and using a leave-one-out approach, we computed the balanced accuracy of class label assignments (see ‘Materials and Methods’ section). For most class variables, the performance of the trained classifier was above the performance of a classifier assigning labels at random (Supplementary Table S4). Importantly, misclassification was rare but was more common between total DNA input and immunoglobulin G controls (Figure 4).

### Similarity between Arpeggio profiles is indicative of biological functions

We showed that the Arpeggio profiles could be used to train classifiers that suggest annotation for new experiments. We sought to extend this paradigm and address whether, given a new set of experiments, Arpeggio profiles could be used to rapidly select available ChIP-seq experiments that would enable a more complete description of the biological mechanism of interest. Typically, this analysis involves identification of peaks from the ChIP-seq signals and the study of the overlap between events in two or more different experiments (8). For this reason, we compared the proximity mapping determined by the Arpeggio profiles with the proximity score determined using the Jaccard distance of peak overlaps.

For each ChIP-seq experiment, peaks were identified using the Qeseq program (61), assigning to each experiment the best matching control as described in the previous sections. We set the fragment size to 150 bp for experiments using MNase digestion and to 250 bp for experiments using sonication (see Supplementary Table S2). We considered two peaks to be overlapping if they shared at least 1 bp. For any pair of experiments, the total number of peaks was computed as the sum of the number of peaks in each experiment, minus the number of overlapping peaks. The pairwise peak overlap score was computed using the Jaccard distance, namely, one minus the ratio between the number of overlapping peaks and the total number of peaks. In contrast to our Arpeggio approach, peak overlap does not directly allow cross-species comparison. To ensure a fair comparison between data representations, we split our data set into a set of human samples (*n* = 541) and a set of murine samples (*n* = 237) and analyzed them separately.

Applying PCA to the peak overlap Jaccard distance matrix revealed that, for the human set, 365 principal components were needed to capture 85% of the variability in the data. In contrast, 85% of the variability of the pairwise distance matrix of the Fourier transforms of the Arpeggio profiles was captured by the six leading principal components. Thus, the Arpeggio MDS provides a more compact representation of the data.

Next, for each class variable, we studied the classification performance using three distance measures: peak overlap Jaccard distance, inverse correlation measure between spectral density of Arpeggio profiles and Euclidean distance between Arpeggio MDS coordinates. We analyzed human and mouse samples separately. For each class variable, we quantified the performance using the Area Under the receiver operator characteristic Curve (AUC) as described in the ‘Materials and Methods’ section.

**Figure 5.**
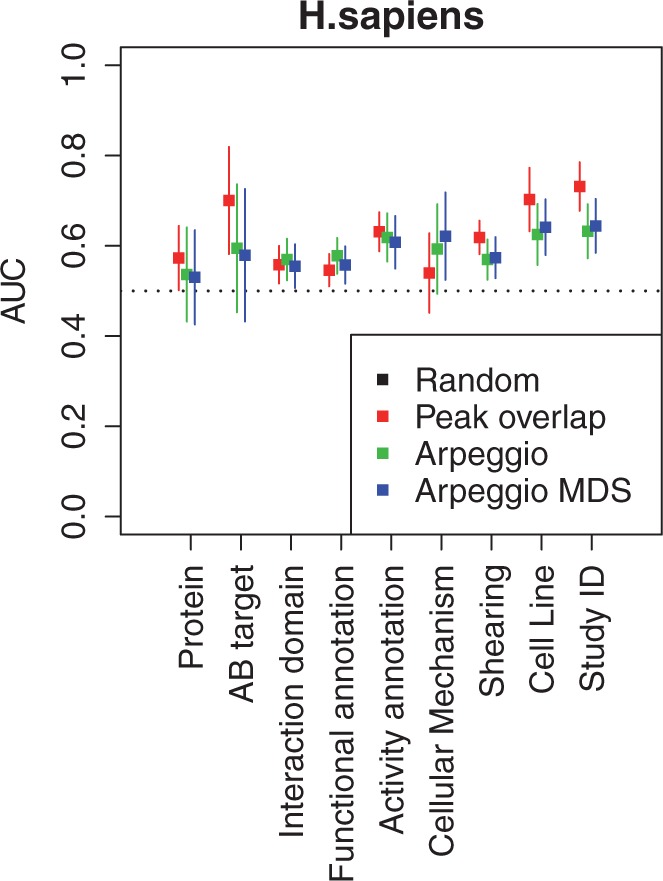
Classification of biological and experimental factors in terms of peak-based and Arpeggio-based ChIP-seq signatures for human samples. The comparison was performed based on proximity computed using peak overlap between pairs of experiments, inverse correlation of Arpeggio spectral densities or Euclidean distances of Arpeggio MDS coordinates. The AUC of a classifier assigning random labels is shown as a black dashed line. Compared with peak overlap, proximity based on Arpeggio MDS had a higher median performance of predicting cellular mechanism.

Application of a k-nearest neighbor classifier with k = 1 to these three distance measures resulted in comparable performances in most class variables. However, proximity between Arpeggio MDS profiles as compared with peak overlap Jaccard distance reflected higher association for cellular mechanisms. This was more evident in the human set where the larger number of experiments corresponded to smaller error bars (Figure 5).

Arpeggio retains information related to mode of binding and performs best in clustering cellular mechanism, in contrast peak overlap contains information about binding locations and best clusters more variables such as batch effects associated with the Study ID, and cell line (Supplementary Table S5, Figure 5 and Supplementary Figure S8).

### Reading biological features from Arpeggio profiles

In the previous sections, we have provided evidence that Arpeggio profiles and their spectral densities can be used to rapidly compare a large number of experiments. In this section, we show that Arpeggio profiles can also be used to derive biologically and technically meaningful information.

**Figure 6.**
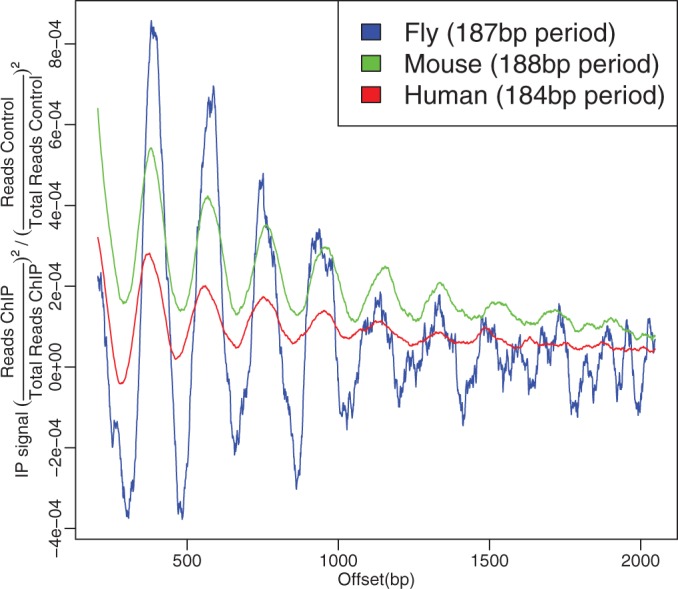
H3K27me3 shows similar periodicity across fly, mouse and human. Periodicity was calculated using the local maxima of the spectral density for the range of the Arpeggio profiles shown.

For instance, H3K27me3 Arpeggio profiles exhibited distinct periodicity with high amplitudes of oscillation (Figure 1). This suggested a highly ordered array of nucleosomes consistent with a static chromatin structure where nucleosomes are precisely positioned. This agreed well with the known role of Polycomb and H3K27 tri-methylation in transcriptional repression and heterochromatin formation. We recall that the profile represents an aggregate of binding events across the whole genome: the clear and distinct periodicity for H3K27me3 suggested that nucleosomes with the tri-methylated H3K27 mark had remarkably constant periodicities throughout the genome (4). Further, we show that this periodicity and the width of the signal (number of clear oscillations) is similar across species (Figure 6).

Another oscillatory pattern can be observed for Histone 3 Lysine 36 tri-methylation (H3K36me3). This histone modification mark is deposited along with actively transcribing PolII complexes and is by far the most reliable histone mark for actively transcribed genes. In contrast to H3K27me3 profiles where the oscillation is only slowly dampening due to the static chromatin structure imposed by this mark, H3K36me3 profiles show a central peak surrounded by slightly smaller peaks with a rapidly degrading oscillation, indicative of a fluidic chromatin state where nucleosomes are not precisely positioned. This is in agreement with this mark being found in actively transcribed genes as the nucleosomes in actively transcribed chromatin are perturbed by the passing polymerase complexes (Figure 1).

**Figure 7.**
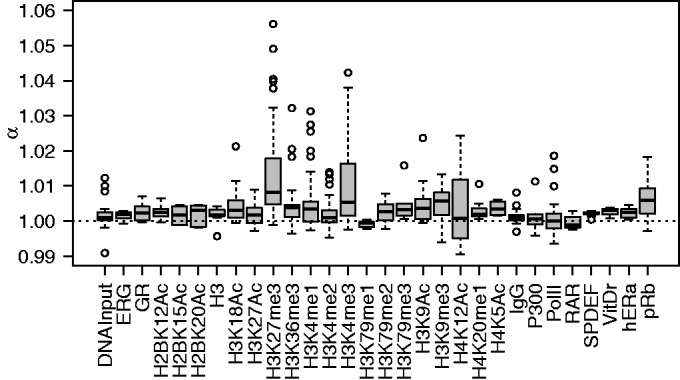
Flexibility of nucleosome spacing across different experiments. Flexible spacing, or isolated events (Supplementary Figure S2), is represented by ratios close to one. Ratios clearly above one indicate strict spacing of 190 bp between events (Supplementary Figure S4). Histone modifications associated with heterochromatin exhibit higher ratios, indicating less flexibility.

The difference in chromatin state is particularly evident in the harmonic analysis of Arpeggio profiles. Inspired by previous work on nucleosome spacing (60), we computed the ratio:
(9)
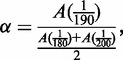

where 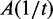 is the magnitude of the *t* bp length scale in the spectral density. H3K27me3 exhibited a stronger α compared with H3K36me3 (Figure 7), which suggests flexibility in the spacing of nucleosomes carrying H3K36me3 mark. Interestingly, the ratio α was also large in Histone 3 Lysine 4 tri-methylation (H3K4me3) and in the Retinoblastoma protein (pRb). Although the reasons for such a precise placement of H3K4me3 are unclear, pRb is an important factor in establishing heterochromatin.

The effects of open fluidic chromatin states are stronger in the Arpeggio profiles of histone acetylations. These profiles are characterized by a high central peak surrounded by unorganized oscillations. These profiles are clearly indicative of an open fluid chromatin configuration, which is consistent with actively transcribed regions (Figure 1).

However, the reference ChIP-seq signal for open actively transcribed chromatin is the Arpeggio profile of RNA Polymerase II. The PolII complexes move along actively transcribed genes; thus, the ChIP-seq information is spread over a relatively large spatial region. For this reason, PolII ChIP experiments need significant sequencing depth to obtain a good picture of PolII activity. The Arpeggio profiles for PolII show a strong central signal surrounded by two shoulders, each flanked by disorganized nucleosomes (Figure 2). The central peak is where PolII is located most closely to the DNA strand, and thus efficient cross-linking can occur. However, PolII is part of a large ‘holoenzyme’ transcription machinery, and the surrounding, smaller humps are likely where this complex is close enough to the DNA strand to be cross-linked and enriched in a ChIP-seq experiment. The unorganized pattern surrounding these peaks is likely a result of the actively transcribing PolII complex. As it moves through the chromatin, it perturbs and/or disassembles nucleosomes in front and re-deposits them behind giving highly disorganized chromatin structure (Figure 2).

Other proteins, such as Androgen Receptor, SPDEF (SAM pointed domain-containing Ets transcription factor), ERG (Ets-Related Gene), FL1 (Follicular lymphoma, susceptibility to, 1), display typical site-specific DNA-binding profiles of transcription factors in which a strong signal occurs at the binding site, accompanied by disorganized surrounding patterns indicative of active, fluidic chromatin (Figure 2).

## DISCUSSION

The contribution of this study is the design of a compact ChIP-seq data representation based on the denoised autocorrelation. We show that use of this compact data representation has several advantages that facilitates efficient computation and data storage, linking the cellular mechanisms of protein targets in novel ChIP-seq experiments to data in current repositories, low-dimensional organization of large repositories of ChIP-seq data that facilitates exploratory data analysis, cross-species and cross-cell line comparisons, extraction of technical features such as fragment length distribution, and biological relevant interpretation.

Large collections of ChIP-seq data have been leveraged to gain new biological insights (8). The volume of ChIP-seq data in public repositories has noticeably increased in recent years. Typically, users retrieve samples based on their prior knowledge and expectations of the biological system. Currently available data retrieval systems are based on matching qualitative annotations such as organism, cell-type, condition and specific immunoprecipitated protein. We suggest the use of our novel compression technique, Arpeggio, to enable searching for samples similar to the query experiment in terms of quantitative patterns present in theirs signals, thus facilitating novel biological discoveries. We note that non-linear data compression approaches applied to the autocorrelation functions can organize the data and reveal new insights (see diffusion map analysis Supplementary Note B). We present Arpeggio profiles and their spectral densities. This low-dimensional harmonic data representation can be used for selecting publicly available experiments that are biologically related to an experiment of interest. The Arpeggio profiles are computed from the autocorrelation of ChIP-seq signals, which have been previously explored in the context of data quality assessment (7).

In contrast to previous approaches, we applied signal processing techniques to derive a profile of the IP autocorrelation that is diminished in technical variability and requires little pre-filtering. We found that Arpeggio profiles were remarkably organized in four main categories, corresponding to intuitive classes of structural interactions: factors showed peaks with sharply decaying tails; polymerases showed peaks as well but with slowly decaying tails; histone modifications showed damped oscillations corresponding to trains of peaks at fixed distances from one another; lastly, controls showed a single pulse sharper than the peak of factors, indicating that, as expected, sequenced reads from total DNA inputs have no recurrent properties. Typical binding patterns of a particular protein–chromatin interaction are obscured by noise. Arpeggio profiles overcome this problem by aggregating the recurrent patterns of protein–chromatin interaction. The quality of autocorrelation also improves quicker than the read coverage density as the number of reads increases. In peak finding, the average coverage is expected to increase linearly, on the order of 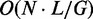, where *N* is the number of sequenced reads, *L* is the read-length and *G* is the size of the genome; in contrast, the number of distances between reads contributing to the computation of the autocorrelation scales quadratically, in the worst case as 
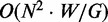
, where *W* is the maximum lag at which the autocorrelation is evaluated, where 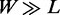. We note that Arpeggio profiles do not provide the location of such binding events; however, we plan to further develop these characteristic spectral binding patterns for locating peaks.

In this work, we used an unsupervised approach to organize a large volume of ChIP-seq experiments. We show that close proximity between the denoised spectral densities of two different proteins is often associated with similar cellular mechanism. In this work, we manually annotated 806 of the 14 306 currently marked as ChIP-seq samples in the SRA. Databases are expected to evolve to be more structured and enable automatic retrieval, eliminating the need for data entry tasks and allowing us to organize thousands of samples at a time. Moreover high-throughput sequencing is becoming cheap, facilitating the mass survey of novel ChIP targets for which function is yet to be determined. Applying the proposed spectral representation to thousands of existing annotated ChIP-seq experiments will allow us to screen these new ChIP targets reducing the resources required to elucidate their functions. Interpretation of spectral patterns in many fields of science and engineering (i.e. radiology, control systems analysis, imaging) is often the product of years of study. In this study, we focused on properties that discriminate coarse categories of protein–chromatin interaction. There is a wealth of knowledge hidden in Arpeggio representation, and we anticipate that with increasing database size and quality, it will provide information on a much finer scale.

## SUPPLEMENTARY DATA

Supplementary Data are available at NAR Online, including [68–122].

## FUNDING

National Institute of Health [T15 LM07056 to K.S.] and [CA-158167 to Y.K.]; Yale Cancer Center translational research pilot funds (to F.P. and F.S.); American Cancer Society Award [M130572 to F.S.]; the American-Italian Cancer Foundation [Post-Doctoral Research Fellowship to F.S.]; the Peter T. Rowley Breast Cancer Research Projects funded by the New York State Department of Health [FAU 0812160900 Y.K.]. Funding for open access charge: The Peter T. Rowley Breast Cancer Research Projects funded by the New York State Department of Health [FAU 0812160900 Y.K.].

*Conflict of interest statement*. None declared.

## Supplementary Material

Supplementary Data
