# Supplementary material for: Arpeggio: harmonic compression of ChIP-seq data reveals protein-chromatin interaction signatures
Source: Nucleic Acids Res. 2013 Jul 19;41(16):e161. doi: 10.1093/nar/gkt627 (PMC3763565; doi:10.1093/nar/gkt627)
Supplement: Supplementary Data [file supp_41_16_e161__index.html]

Arpeggio: harmonic compression of ChIP-seq data reveals protein-chromatin interaction signatures — Arpeggio: harmonic compression of ChIP-seq data reveals protein-chromatin interaction signatures — Supplementary Data 

# Arpeggio: harmonic compression of ChIP-seq data reveals protein-chromatin interaction signatures

## 

files

**Files in this Data Supplement:**

- Supplementary Data - pdf file
