# Supplementary material for: Arpeggio: harmonic compression of ChIP-seq data reveals protein-chromatin interaction signatures
Source: Nucleic Acids Res. 2013 Jul 19;41(16):e161. doi: 10.1093/nar/gkt627 (PMC3763565; doi:10.1093/nar/gkt627)
Supplement: Supplementary Data [file supp_gkt627_nar-01284-met-k-2013-File011.pdf]

Supplementary Data
Arpeggio: harmonic compression of ChIP-seq data reveals protein-chromatin interaction signatures

Kelly Patrick Stanton, Fabio Parisi, Francesco Strino,
Neta Rabin, Patrik Asp and Yuval Kluger

Contents

A Supplementary Methods **2**
A.1 Comparison of estimated fragment size distributions 2
A.1.1 Data and preprocessing 2
A.1.2 Fragment length distribution from paired-end data 2
A.1.3 Fragment length distribution from Arpeggio 2

B Diffusion map of the autocorrelation of ChIP-seq signals reveals a coarse structure associated with functional properties of the antibody targets **3**
B.1 Introduction 3
B.2 Methods 3
B.2.1 Diffusion map framework 3
B.2.2 Gaussian diffusion kernel 4
B.2.3 Choosing the scale parameter ϵ 5
B.2.4 Embedding of the data 5
B.3 Diffusion map of ChIP-seq autocorrelation functions 5

Supplementary Tables **7**

Supplementary Figures **40**

Supplementary References **49**

A Supplementary Methods

A.1 Comparison of estimated fragment size distributions

A.1.1 Data and preprocessing

Reads from the Short Read Archive (SRA) total DNA Input sample SRR121540 [68] were aligned to the Drosophila melanogaster genome with the Bowtie aligner [49] with parameters "-n2 -k1 -m1 --best --strata --maxins 1000".

A.1.2 Fragment length distribution from paired-end data

The empirical fragment length distribution for the paired-end data was obtained as the histogram of the distances between mapped reads from the same fragment.

A.1.3 Fragment length distribution from Arpeggio

We considered one read at random from each pair of reads from the same fragment thus generating a single-end read library. This single-end library was mapped to the reference genome as described in the main text. Next, we applied our technique to infer the fragment length distribution as described in the Results.

B Diffusion map of the autocorrelation of ChIP-seq signals reveals a coarse structure associated with functional properties of the antibody targets

B.1 Introduction

Diffusion maps and diffusion distances provide a method for finding meaningful geometric descriptions in low-dimensional datasets described by a large number of features. The diffusion maps construct coordinates that parameterize the data using a local-preserving metric called diffusion distance. The non-linear dimensionality reduction, which reveals global geometric information, is then constructed by local overlapping structures. We applied a diffusion map method to investigate the organization of the space of autocorrelation functions prior to deconvolving the **true IP signal** and identified a rough structure associated with functional properties of the antibody targets.

B.2 Methods

B.2.1 Diffusion map framework

Let Γ be a set of points in R^n. In order to study the intrinsic geometry of the set Γ, we construct a graph $G = (\Gamma, W)$ with a kernel $W = w(x, y)$. The kernel, which is a weight function, measures the pair-wise similarity between points and it satisfies three properties: i) symmetry, $w(x, y) = w(y, x)$; ii) positive-preserving, $w(x, y) \geq 0, \forall x, y \in \Gamma$; iii) positive semi-definite, for all real-valued bounded functions $f \in \Gamma$, $\sum_{x\in\Gamma} \sum_{y\in\Gamma} w(x, y) f(x) f(y) \geq 0$. Choosing a kernel that satisfies these properties allows normalizing W into a Markov transition matrix P, which is constructed as follows:

$$P = p(x, y) = \frac{w(x, y)}{d(x)}, d(x) = \sum_{y\in\Gamma} w(x, y), \tag{1}$$

where $d(x)$ is the degree of the node x. In spectral graph theory, this normalization is known as the weighted Graph Laplacian normalization [69]. The transition matrix P is conjugate to a symmetric matrix A given by

$$a(x, y) = \sqrt{d(x)} p(x, y) \frac{1}{\sqrt{d(y)}}, \tag{2}$$

or, in matrix notation, $A = D^{1/2} P D^{-1/2}$, where D is the diagonal matrix with values $\sum_y w(x, y)$ along the diagonal. The symmetric matrix A has n real eigenvalues $\{\lambda_k\}_{k=0}^{n-1}$ and a set of orthogonal eigenvectors $\{v_k\}$ in R^n. Thus, A has the following spectral decomposition:

$$a(x, y) = \sum_{k\geq 0} \lambda_k v_k(x) v_k(y). \tag{3}$$

Since P is conjugate to A, the eigenvalues of both matrices are identical. In addition, if $\{\phi_k\}$ and $\{\psi_k\}$ are the corresponding left and right eigenvectors of P, then we have the following connections:

$$\phi_k = D^{1/2} v_k, \psi_k = D^{-1/2} v_k. \tag{4}$$

From the orthonormality of $\{v_i\}$ and equation 4 it follows that ϕ_i and ψ_i are biorthonormal, which means $\langle \phi_l, \psi_k \rangle = \delta_{lk}$. Combining equations 3 and 4 with the biorthogonality of $\{\phi_k\}$ and $\{\psi_k\}$ leads to the following eigendecomposition of the transition matrix P

$$p(x, y) = \sum_{k \geq 0} \lambda_k \psi_k(x) \phi_k(y). \tag{5}$$

Because of the fast decay of the spectrum, only a few terms are required to achieve sufficient accuracy in the sum. The family of diffusion maps, defined by

$$\Psi(x) = \{\lambda_1 \psi_1(x), \lambda_2 \psi_2(x), \ldots\} \tag{6}$$

embeds the dataset into a Euclidean space. This embedding is a new parametrization of the data into a lower space. In the present analysis we used the Gaussian diffusion kernel.

B.2.2 Gaussian diffusion kernel

The construction of diffusion maps and diffusion distance may vary according to the way the kernel is defined. We decided to focus on kernels of the form $w_\epsilon(x, y) = h\left(-\|x - y\|^2/2\epsilon\right)$ that are direction independent. A common choice is the Gaussian kernel $w_\epsilon(x, y) = e^{-|x-y|^2/2\epsilon}$. A general form of a kernel, with a parameter α that controls the normalization type, given by

$$w_\epsilon^{(\alpha)}(x, y) = \frac{w_\epsilon(x, y)}{q^\alpha(x) q^\alpha(y)}, q(x) = \sum_{y \in \Gamma} w_\epsilon(x, y) \tag{7}$$

was introduced in [70].

The transition matrix is defined by

$$p_\epsilon^{(\alpha(x,y))} = \frac{w_\epsilon^{(\alpha)}(x, y)}{d_\epsilon^{(\alpha)}(x)}, d_\epsilon^{(\alpha)}(y) = \sum_{y \in \Gamma} w_\epsilon^{(\alpha)}(x, y) \tag{8}$$

The value of the parameter α defines the effect of the density in the diffusion kernel. The asymptotic behavior, which is obtained as $\epsilon \to 0$, generates different infinitesimal operator for different values of α. Coifman et al. showed that the eigenfunction of the Markov matrix P_ϵ^α approximates a symmetric Schrödinger operator [70–72]. In the present study we used $\alpha = 1$, which converges to the Laplace Beltrami operator [69].

B.2.3 Choosing the scale parameter ϵ

The diffusion maps reveals and extracts global information in local structures. The scale parameter ϵ determines the size of the local neighborhood of each point. In this analysis we used the method described in [73] for setting the value of ϵ. A large ϵ defines a wide neighborhood and thus produces a coarse analysis of the data since most of the neighborhoods will contain a large number of data points. In contrast, for a small ϵ, many neighborhoods will contain a single data point. Clearly, an appropriate ϵ should be between these two cases and should stem from the data. For a dataset Γ, which contains m data points, the pair-wise Euclidean distance matrix between data points in Γ is defined as $\bar{D} = \{d_{ij}\}_{i,j=1...m}$. The median determines ϵ as

$$\epsilon = \text{median}[\{d_{ij}\}_{i,j=1...m}] \tag{9}$$

The median of $\bar{D}$ provides an estimate of the average pair-wise distance that is robust to outliers.

B.2.4 Embedding of the data

We embedded the profiles of local distances between 200bp and 1kbp using our embedding algorithm. The embedding algorithm consists of three steps: data samples are re-scaled to have a unit norm, data is whitened using PCA whitening and then it is embedded using diffusion maps.

In details, in order to organize the dataset Γ according to the shapes of the measurements $\{x_i\}_{i=1}^m$, each measurement is first divided by its norm. The normalized dataset, denoted by $\tilde{\Gamma}$, is thus obtained by $\tilde{\Gamma} = \{x_i/\|x_i\|\}_{i=1}^m$. Then we apply PCA whitening, which includes PCA and normalization along the principal component directions. The whitened, uncorrelated dataset is denoted by $\tilde{\Gamma}_w$. $\tilde{\Gamma}_w$ is obtained by $\tilde{\Gamma}_w = \Delta^{-1/2}\Phi^T\tilde{\Gamma}$, where Δ and Φ are the corresponding matrices of the eigenvalues and eigenvectors of the covariance matrix of $\tilde{\Gamma}$, respectively. We then embed the data using diffusion maps. Namely, the first k uncorrelated components, which belong to the matrix $\tilde{\Gamma}_w$, are used as input for the diffusion maps algorithm. The leading l diffusion maps coordinates ($l < k$) are used to embed the data.

To avoid artifacts due to inefficient PCR amplifications, we trimmed the autocorrelation profiles restricting them in the range 200bp $\leq \tau \leq$ 1000bp. We set the number of whitened PCA coordinates to $k = 10$, although, from the spectrum of the eigenvalues, we concluded that $k = 6$ was sufficient to capture more than 90% of the variability in the data.

B.3 Diffusion map of ChIP-seq autocorrelation functions

We embedded the autocorrelation functions of ChIP-seq signals using our embedding algorithm with a Gaussian diffusion kernel. From the diffusion map coordinates, we found that the samples are organized in branches having a common origin (Figure B.3). In addition, we grouped experiments according

to their functions and similarity in terms of IP-ed proteins. We noticed that groups tended to coarsely form local clusters in the low-dimensional projection (Figure B.3). The diffusion coordinates organize the data points based on the similarity of their profiles. Promisingly, the first three coordinates could be roughly ascribed to four functional groups: transcription factors, histone marks, polymerases and controls. Although it may be hard to clearly define the dominant property of the data that is emphasized in each diffusion coordinate, one can see that the diffusion coordinates arrange the data based on characteristics like oscillations, spatial decay of the signal and noise (Figure B.3).

Intuitively, the four groups of experiments highlighted by the diffusion map were compatible with the following four scenarios. **Localized:** the interaction occurs only at certain genomic locations, each location being very far from all other locations in terms of genomic distance (e.g. a transcription factor): in terms of autocorrelation, this scenario should correspond to an isolated pulse centered at $\tau = 0$. **Periodic.** The interaction occurs at certain genomic locations, which are arranged in multiples each at short fixed distances from one another (e.g. a histone modification mark): the corresponding autocorrelation should exhibit periodicity with period equal to the inter-interaction distance. **Elongated.** The interaction initially occurs at fixed locations, but subsequently, the protein moves along the DNA and detaches after a certain distance (e.g. Polymerases): since shorter distances are common to all interaction events, while the longer distances are uncommon, the corresponding autocorrelation is expected to be a decaying function. **Random.** Interactions occur at random locations (e.g. breakpoints from sonication): as each location has the same probability of being selected: the corresponding autocorrelation should correspond to a constant number, or a slowly decaying function, regardless of the lag.

Despite the power of this approach, as we have shown in the main text, deconvolution of the **true IP signal** using DSP techniques, enabled the use of simpler techniques, such as PCA, to achieve a clearer characterization of the space of ChIP-seq signals. PCA coordinates are easier to compute and quicker to update, and thus preferrable for indexing large databases.

Supplementary Tables

Table S1: **Summary of the ChIP-Seq experiments used in the study**

Experiment ID	Antibody target	Organism	SRA Experiment	SRA Run	Reference
AR_VCaP_NULL_1	AR	H.sapiens	SRX021799	SRR054876	[42]
BAP18_HeLa_NULL_1	BAP18	H.sapiens	SRX023657	SRR060530	[40]
BAP18_HeLa_NULL_2	BAP18	H.sapiens	SRX023651	SRR060524	[40]
bCAT_ColCan_NULL_1	bCAT	H.sapiens	SRX017112	SRR038620	[12]
BRG1_HESC_NULL_1	BRG1	H.sapiens	SRX027488	SRR067376	[36]
CDX2_CACO2_DIFF_1	CDX2	H.sapiens	SRX026264	SRR064947	[41]
CDX2_CACO2_DIFF_2	CDX2	H.sapiens	SRX026264	SRR064948	[41]
CDX2_CACO2_PROL_1	CDX2	H.sapiens	SRX026263	SRR064946	[41]
CEBPA_HEPG2_NULL_1	CEBPA	H.sapiens	ERX004461	ERR011990	[39]
CEBPA_HEPG2_NULL_2	CEBPA	H.sapiens	ERX004459	ERR011989	[39]
CTCF_H1ES_NULL_1	CTCF	H.sapiens	SRX031244	SRR073078	[7, 8]
CTCF_H1ES_NULL_2	CTCF	H.sapiens	SRX031244	SRR073079	[7, 8]
CTCF_HEPG2_NULL_1	CTCF	H.sapiens	ERX004460	ERR011987	[39]
CTCF_HEPG2_NULL_2	CTCF	H.sapiens	ERX004456	ERR011988	[39]
CTCF_HUVEC_NULL_1	CTCF	H.sapiens	SRX031246	SRR073082	[7, 8]
CTCF_HUVEC_NULL_2	CTCF	H.sapiens	SRX031246	SRR073083	[7, 8]
CTCF_HeLaS3_NULL_1	CTCF	H.sapiens	SRX031245	SRR073080	[7, 8]
CTCF_HeLaS3_NULL_2	CTCF	H.sapiens	SRX031245	SRR073081	[7, 8]
CTCF_K562_NULL_1	CTCF	H.sapiens	SRX031247	SRR073084	[7, 8]
CTCF_K562_NULL_2	CTCF	H.sapiens	SRX031247	SRR073085	[7, 8]
CTCF_K562_NULL_3	CTCF	H.sapiens	SRX031247	SRR073086	[7, 8]
CTCF_MCF7_ER_1	CTCF	H.sapiens	ERX004466	ERR012001	[39]
CTCF_MCF7_NULL_1	CTCF	H.sapiens	ERX004470	ERR012002	[39]
CTCF_MCF7_NULL_2	CTCF	H.sapiens	ERX008596	ERR022043	[22]
CTCF_MCF7_NULL_3	CTCF	H.sapiens	ERX008591	ERR022045	[22]
CTCF_MCF7_NULL_4	CTCF	H.sapiens	ERX008590	ERR022056	[22]
CTCF_MCF7_NULL_5	CTCF	H.sapiens	ERX008611	ERR022047	[22]
CTCF_MEF_NULL_1	CTCF	M.musculus	SRX022698	SRR058991	[27]
CTCF_MEF_NULL_2	CTCF	M.musculus	SRX022699	SRR058992	[27]
CTCF_NHEK_NULL_1	CTCF	H.sapiens	SRX031248	SRR073087	[7, 8]
CTCF_NHEK_NULL_2	CTCF	H.sapiens	SRX031248	SRR073088	[7, 8]
CTCF_TCELL_NULL_1	CTCF	H.sapiens	SRX000138	SRR001460	[5]
DNAInput_HEPG2_NULL_1	DNAInput	H.sapiens	ERX004451	ERR011991	[39]
DNAInput_MCF7_ER_1	DNAInput	H.sapiens	ERX004448	ERR011972	[39]
DNAInput_MCF7_NULL_1	DNAInput	H.sapiens	ERX004469	ERR011971	[39]
DNAInput_MCF7_NULL_2	DNAInput	H.sapiens	ERX008601	ERR022054	[22]
DNAInput_MCF7_NULL_3	DNAInput	H.sapiens	ERX008599	ERR022055	[22]
DNAInput_SalGlan_NULL_1	DNAInput	D.melanogaster	ERX004311	ERR011351	[32]
DNAInput_T47D_NULL_1	DNAInput	H.sapiens	ERX008616	ERR022012	[22]
DNAInput_T47D_NULL_2	DNAInput	H.sapiens	ERX008609	ERR022013	[22]
DNAInput_WLiver_NULL_1	DNAInput	M.musculus	SRX020707	SRR043782	[21]
DNAInput_WLiver_NULL_2	DNAInput	M.musculus	SRX020706	SRR043783	[21]
DNAInput_WLiver_NULL_3	DNAInput	M.musculus	SRX020706	SRR043784	[21]
DNAInput_WLiver_NULL_4	DNAInput	M.musculus	SRX020705	SRR043781	[21]
DNAInput_ZR75_NULL_1	DNAInput	H.sapiens	ERX008571	ERR022017	[22]
DNAInput_ZR75_NULL_2	DNAInput	H.sapiens	ERX008606	ERR022016	[22]
DNAInput_c2c12_MB_1	DNAInput	M.musculus	SRX062124	SRR202920	[4]
DNAInput_c2c12_MB_10	DNAInput	M.musculus	SRX062122	SRR202915	[4]
DNAInput_c2c12_MB_2	DNAInput	M.musculus	SRX062124	SRR202921	[4]
DNAInput_c2c12_MB_3	DNAInput	M.musculus	SRX062124	SRR202922	[4]
DNAInput_c2c12_MB_4	DNAInput	M.musculus	SRX062124	SRR202923	[4]
DNAInput_c2c12_MB_5	DNAInput	M.musculus	SRX062124	SRR202924	[4]
DNAInput_c2c12_MB_6	DNAInput	M.musculus	SRX062124	SRR202925	[4]
DNAInput_c2c12_MB_7	DNAInput	M.musculus	SRX062122	SRR202912	[4]
DNAInput_c2c12_MB_8	DNAInput	M.musculus	SRX062122	SRR202913	[4]
DNAInput_c2c12_MB_9	DNAInput	M.musculus	SRX062122	SRR202914	[4]
DNAInput_c2c12_MT_1	DNAInput	M.musculus	SRX062125	SRR202926	[4]
DNAInput_c2c12_MT_10	DNAInput	M.musculus	SRX062123	SRR202919	[4]
DNAInput_c2c12_MT_2	DNAInput	M.musculus	SRX062125	SRR202927	[4]
DNAInput_c2c12_MT_3	DNAInput	M.musculus	SRX062125	SRR202928	[4]
DNAInput_c2c12_MT_4	DNAInput	M.musculus	SRX062125	SRR202929	[4]
DNAInput_c2c12_MT_5	DNAInput	M.musculus	SRX062125	SRR202930	[4]
DNAInput_c2c12_MT_6	DNAInput	M.musculus	SRX062125	SRR202931	[4]
DNAInput_c2c12_MT_7	DNAInput	M.musculus	SRX062123	SRR202916	[4]
DNAInput_c2c12_MT_8	DNAInput	M.musculus	SRX062123	SRR202917	[4]
DNAInput_c2c12_MT_9	DNAInput	M.musculus	SRX062123	SRR202918	[4]
DNAInput_hESCH1_NULL_1	DNAInput	H.sapiens	SRX027888	SRR067977	[9]
DNAInput_hESCH1_NULL_2	DNAInput	H.sapiens	SRX027887	SRR067976	[9]
DNAInput_hESCH1_NULL_3	DNAInput	H.sapiens	SRX027886	SRR067975	[9]
DNAInput_hESCH1_NULL_4	DNAInput	H.sapiens	SRX027885	SRR067974	[9]
DNAInput_hESCH1_NULL_5	DNAInput	H.sapiens	SRX027884	SRR067973	[9]
DNAInput_hESCH1_NULL_6	DNAInput	H.sapiens	SRX027883	SRR067971	[9]
DNAInput_hESCH1_NULL_7	DNAInput	H.sapiens	SRX027883	SRR067972	[9]
DNAInput_hESCH1_NULL_8	DNAInput	H.sapiens	SRX027882	SRR067970	[9]
E2F1_HeLa_NULL_1	E2F1	H.sapiens	SRX022432	SRR058522	[41]
E2F4_GM06990_NULL_1	E2F4	H.sapiens	SRX019963	SRR042292	[31]
E2F4_MEF_NULL_1	E2F4	M.musculus	SRX027460	SRR067348	n.a.

Continued on next page

Table S1 – *Continued from previous page*

Experiment ID	Antibody target	Organism	SRA Experiment	SRA Run	Reference
ELF1_Jurkat_NULL_1_C	ELF1	H.sapiens	SRX021788	SRR054859	[42]
ERG_CADO_NULL_1_C	ERG	H.sapiens	SRX021784	SRR054853	[42]
ERG_CADO_NULL_2_C	ERG	H.sapiens	SRX021784	SRR054854	[42]
ERG_CADO_NULL_3_C	ERG	H.sapiens	SRX021784	SRR054855	[42]
ERG_CADO_NULL_4_C	ERG	H.sapiens	SRX021654	SRR054758	[42]
ERG_CADO_NULL_5_C	ERG	H.sapiens	SRX021654	SRR054852	[42]
ERG_VCaP_NULL_1	ERG	H.sapiens	SRX021801	SRR054881	[42]
ERG_VCaP_NULL_2	ERG	H.sapiens	SRX021801	SRR054882	[42]
ERG_VCaP_NULL_3	ERG	H.sapiens	SRX021801	SRR054883	[42]
ERG_VCaP_NULL_4	ERG	H.sapiens	SRX021801	SRR054884	[42]
ERG_VCaP_NULL_5	ERG	H.sapiens	SRX021801	SRR054885	[42]
ERG_VCaP_NULL_6	ERG	H.sapiens	SRX021801	SRR054886	[42]
FAIRE_HESC_NULL_1	FAIRE	H.sapiens	SRX027489	SRR067377	[36]
FLI1_SKNMC_NULL_1_C	FLI1	H.sapiens	SRX021794	SRR054870	[42]
FLI1_SKNMC_NULL_2_C	FLI1	H.sapiens	SRX021792	SRR054866	[42]
FLI1_SKNMC_NULL_3_C	FLI1	H.sapiens	SRX021792	SRR054867	[42]
FLI1_SKNMC_NULL_4_C	FLI1	H.sapiens	SRX021792	SRR054868	[42]
FLI1_SKNMC_NULL_5_C	FLI1	H.sapiens	SRX021792	SRR054869	[42]
GABPA_HPC_NULL_1	GABPA	H.sapiens	SRX029315	SRR070250	[45]
GATA2_HSC_NULL_1	GATA2	M.musculus	SRX035992	SRR087806	[32]
GATA6_CACO2_DIFF_1	GATA6	H.sapiens	SRX026266	SRR064950	[41]
GATA6_CACO2_PROL_1	GATA6	H.sapiens	SRX026265	SRR064949	[41]
GATAD1_HeLa_NULL_1	GATAD1	H.sapiens	SRX023649	SRR060522	[40]
GATAD1_HeLa_NULL_2	GATAD1	H.sapiens	SRX023655	SRR060528	[40]
GR_3134_DEX_1	GR	M.musculus	SRX034863	SRR088671	[24]
GR_3134_DEX_2	GR	M.musculus	SRX034863	SRR088672	[24]
GR_3134_DEX_3	GR	M.musculus	SRX034862	SRR088670	[24]
GR_3134_DEX_4	GR	M.musculus	SRX034862	SRR088669	[24]
GR_3134_DEX_5	GR	M.musculus	SRX034861	SRR088663	[24]
GR_3134_DEX_6	GR	M.musculus	SRX034861	SRR088664	[24]
GR_3134_NULL_1	GR	M.musculus	SRX034860	SRR088661	[24]
GR_3134_NULL_2	GR	M.musculus	SRX034860	SRR088662	[24]
GR_ATT20_DEX_1	GR	M.musculus	SRX034870	SRR088686	[24]
GR_ATT20_DEX_2	GR	M.musculus	SRX034870	SRR088687	[24]
GR_ATT20_DEX_3	GR	M.musculus	SRX034870	SRR088688	[24]
GR_ATT20_DEX_4	GR	M.musculus	SRX034869	SRR088683	[24]
GR_ATT20_DEX_5	GR	M.musculus	SRX034869	SRR088684	[24]
GR_ATT20_DEX_6	GR	M.musculus	SRX034869	SRR088685	[24]
GR_ATT20_NULL_1	GR	M.musculus	SRX034868	SRR088682	[24]
GR_ATT20_NULL_2	GR	M.musculus	SRX034868	SRR088681	[24]
GR_ATT20_NULL_3	GR	M.musculus	SRX034868	SRR088680	[24]
GR_ATT20_NULL_4	GR	M.musculus	SRX034867	SRR088677	[24]
GR_ATT20_NULL_5	GR	M.musculus	SRX034867	SRR088678	[24]
GR_ATT20_NULL_6	GR	M.musculus	SRX034867	SRR088679	[24]
H2AK5Ac_HESC_NULL_1	H2AK5Ac	H.sapiens	SRX027889	SRR067978	[9]
H2AK5Ac_HESC_NULL_2	H2AK5Ac	H.sapiens	SRX027691	SRR067766	[9]
H2AK5Ac_IMR90_NULL_1	H2AK5Ac	H.sapiens	SRX017487	SRR037566	[9]
H2AK5Ac_IMR90_NULL_2	H2AK5Ac	H.sapiens	SRX017486	SRR037565	[9]
H2AK5Ac_IMR90_NULL_3	H2AK5Ac	H.sapiens	SRX017485	SRR037564	[9]
H2AZ_TCELL_NULL_1	H2AZ	H.sapiens	SRX000139	SRR001442	[5]
H2AZ_TCELL_NULL_2	H2AZ	H.sapiens	SRX000139	SRR001459	[5]
H2BK120Ac_HESC_NULL_1	H2BK120Ac	H.sapiens	SRX027844	SRR067930	[9]
H2BK120Ac_IMR90_NULL_1	H2BK120Ac	H.sapiens	SRX017489	SRR037569	[9]
H2BK120Ac_IMR90_NULL_2	H2BK120Ac	H.sapiens	SRX017489	SRR037570	[9]
H2BK120Ac_IMR90_NULL_3	H2BK120Ac	H.sapiens	SRX017488	SRR037567	[9]
H2BK120Ac_IMR90_NULL_4	H2BK120Ac	H.sapiens	SRX017488	SRR037568	[9]
H2BK12Ac_HESC_NULL_1	H2BK12Ac	H.sapiens	SRX027846	SRR067932	[9]
H2BK12Ac_HESC_NULL_2	H2BK12Ac	H.sapiens	SRX027845	SRR067931	[9]
H2BK12Ac_IMR90_NULL_1	H2BK12Ac	H.sapiens	SRX017493	SRR037574	[9]
H2BK12Ac_IMR90_NULL_2	H2BK12Ac	H.sapiens	SRX017492	SRR037573	[9]
H2BK12Ac_IMR90_NULL_3	H2BK12Ac	H.sapiens	SRX017491	SRR037572	[9]
H2BK12Ac_IMR90_NULL_4	H2BK12Ac	H.sapiens	SRX017490	SRR037571	[9]
H2BK15Ac_HESC_NULL_1	H2BK15Ac	H.sapiens	SRX027848	SRR067934	[9]
H2BK15Ac_HESC_NULL_2	H2BK15Ac	H.sapiens	SRX027847	SRR067933	[9]
H2BK15Ac_IMR90_NULL_1	H2BK15Ac	H.sapiens	SRX017497	SRR037578	[9]
H2BK15Ac_IMR90_NULL_2	H2BK15Ac	H.sapiens	SRX017496	SRR037577	[9]
H2BK15Ac_IMR90_NULL_3	H2BK15Ac	H.sapiens	SRX017495	SRR037576	[9]
H2BK15Ac_IMR90_NULL_4	H2BK15Ac	H.sapiens	SRX017494	SRR037575	[9]
H2BK20Ac_HESC_NULL_1	H2BK20Ac	H.sapiens	SRX027850	SRR067936	[9]
H2BK20Ac_HESC_NULL_2	H2BK20Ac	H.sapiens	SRX027849	SRR067935	[9]
H2BK20Ac_IMR90_NULL_1	H2BK20Ac	H.sapiens	SRX017499	SRR037581	[9]
H2BK20Ac_IMR90_NULL_2	H2BK20Ac	H.sapiens	SRX017499	SRR037582	[9]
H2BK20Ac_IMR90_NULL_3	H2BK20Ac	H.sapiens	SRX017498	SRR037580	[9]
H2BK20Ac_IMR90_NULL_4	H2BK20Ac	H.sapiens	SRX017498	SRR037579	[9]
H2BK5Ac_HESC_NULL_1	H2BK5Ac	H.sapiens	SRX027852	SRR067938	[9]
H2BK5Ac_HESC_NULL_2	H2BK5Ac	H.sapiens	SRX027851	SRR067937	[9]
H2BK5me1_TCELL_NULL_1	H2BK5me1	H.sapiens	SRX000140	SRR001401	[5]
H2BK5me1_TCELL_NULL_2	H2BK5me1	H.sapiens	SRX000140	SRR001412	[5]
H2BK5me1_TCELL_NULL_3	H2BK5me1	H.sapiens	SRX000140	SRR001413	[5]
H3_38b9_NULL_1_B	H3	M.musculus	SRX026100	SRR064706	n.a.
H3_ES_NULL_1	H3	M.musculus	SRX027333	SRR066768	[14]
H3_ES_NULL_1_B	H3	M.musculus	SRX026102	SRR064708	n.a.

Continued on next page

Table S1 – *Continued from previous page*

Experiment ID	Antibody target	Organism	SRA Experiment	SRA Run	Reference
H3_ES_TGFB_1_B	H3	M.musculus	SRX026103	SRR064709	n.a.
H3_MEF_NULL_1	H3	M.musculus	SRX027350	SRR066785	[14]
H3_WLiver_NULL_1	H3	M.musculus	SRX027341	SRR066776	[14]
H3_c2c12_NULL_1_B	H3	M.musculus	SRX026101	SRR064707	n.a.
H3_proB_NULL_1	H3	M.musculus	SRX027346	SRR066781	[14]
H3K14Ac_IMR90_NULL_1	H3K14Ac	H.sapiens	SRX017502	SRR037585	[9]
H3K14Ac_IMR90_NULL_2	H3K14Ac	H.sapiens	SRX017502	SRR037586	[9]
H3K14Ac_IMR90_NULL_3	H3K14Ac	H.sapiens	SRX017501	SRR037584	[9]
H3K14Ac_IMR90_NULL_4	H3K14Ac	H.sapiens	SRX017500	SRR037583	[9]
H3K18Ac_HESC_NULL_1	H3K18Ac	H.sapiens	SRX027853	SRR067939	[9]
H3K18Ac_HESC_NULL_2	H3K18Ac	H.sapiens	SRX027692	SRR067767	[9]
H3K18Ac_IMR90_NULL_1	H3K18Ac	H.sapiens	SRX017503	SRR037557	[9]
H3K18Ac_IMR90_NULL_2	H3K18Ac	H.sapiens	SRX017503	SRR037559	[9]
H3K18Ac_IMR90_NULL_3	H3K18Ac	H.sapiens	SRX012495	SRR029625	[9]
H3K18Ac_IMR90_NULL_4	H3K18Ac	H.sapiens	SRX012495	SRR029629	[9]
H3K18Ac_c2c12_MB_1	H3K18Ac	M.musculus	SRX062118	SRR202898	[4]
H3K18Ac_c2c12_MB_2	H3K18Ac	M.musculus	SRX062118	SRR202899	[4]
H3K18Ac_c2c12_MB_3	H3K18Ac	M.musculus	SRX062118	SRR202900	[4]
H3K18Ac_c2c12_MT_1	H3K18Ac	M.musculus	SRX062119	SRR202901	[4]
H3K18Ac_c2c12_MT_2	H3K18Ac	M.musculus	SRX062119	SRR202902	[4]
H3K18Ac_c2c12_MT_3	H3K18Ac	M.musculus	SRX062119	SRR202903	[4]
H3K23Ac_IMR90_NULL_1	H3K23Ac	H.sapiens	SRX017505	SRR037589	[9]
H3K23Ac_IMR90_NULL_2	H3K23Ac	H.sapiens	SRX017505	SRR037590	[9]
H3K23Ac_IMR90_NULL_3	H3K23Ac	H.sapiens	SRX017504	SRR037587	[9]
H3K23Ac_IMR90_NULL_4	H3K23Ac	H.sapiens	SRX017504	SRR037588	[9]
H3K23me2_HESC_NULL_1	H3K23me2	H.sapiens	SRX027855	SRR067941	[9]
H3K23me2_HESC_NULL_2	H3K23me2	H.sapiens	SRX027854	SRR067940	[9]
H3K27Ac_ES_NULL_1	H3K27Ac	M.musculus	SRX027332	SRR066767	[14]
H3K27Ac_ES_NULL_2	H3K27Ac	M.musculus	SRX027331	SRR066766	[14]
H3K27Ac_HESC_NULL_1	H3K27Ac	H.sapiens	SRX027856	SRR067942	[9]
H3K27Ac_HESC_NULL_2	H3K27Ac	H.sapiens	SRX012367	SRR029350	[9]
H3K27Ac_HESC_NULL_3	H3K27Ac	H.sapiens	SRX012366	SRR029348	[9]
H3K27Ac_HESC_NULL_4	H3K27Ac	H.sapiens	SRX027485	SRR067373	[36]
H3K27Ac_IMR90_NULL_1	H3K27Ac	H.sapiens	SRX017506	SRR037561	[9]
H3K27Ac_IMR90_NULL_2	H3K27Ac	H.sapiens	SRX012497	SRR029631	[9]
H3K27Ac_IMR90_NULL_3	H3K27Ac	H.sapiens	SRX012497	SRR029632	[9]
H3K27Ac_IMR90_NULL_4	H3K27Ac	H.sapiens	SRX012496	SRR029626	[9]
H3K27Ac_IMR90_NULL_5	H3K27Ac	H.sapiens	SRX012496	SRR029630	[9]
H3K27Ac_NEC_NULL_1	H3K27Ac	H.sapiens	SRX027493	SRR067381	[36]
H3K27Ac_NeuProg_NULL_1	H3K27Ac	M.musculus	SRX027338	SRR066773	[14]
H3K27Ac_WLiver_NULL_1	H3K27Ac	M.musculus	SRX027340	SRR066775	[14]
H3K27Ac_proB_NULL_1	H3K27Ac	M.musculus	SRX027345	SRR066780	[14]
H3K27me1_TCELL_NULL_1	H3K27me1	H.sapiens	SRX000141	SRR001438	[5]
H3K27me1_TCELL_NULL_2	H3K27me1	H.sapiens	SRX000141	SRR001448	[5]
H3K27me1_TCELL_NULL_3	H3K27me1	H.sapiens	SRX000141	SRR001463	[5]
H3K27me1_TCELL_NULL_4	H3K27me1	H.sapiens	SRX000141	SRR001464	[5]
H3K27me2_TCELL_NULL_1	H3K27me2	H.sapiens	SRX000142	SRR001433	[5]
H3K27me2_TCELL_NULL_2	H3K27me2	H.sapiens	SRX000142	SRR001434	[5]
H3K27me2_TCELL_NULL_3	H3K27me2	H.sapiens	SRX000142	SRR001435	[5]
H3K27me2_TCELL_NULL_4	H3K27me2	H.sapiens	SRX000142	SRR001436	[5]
H3K27me3_BAM_NULL_1	H3K27me3	D.melanogaster	SRX017855	SRR038290	[18]
H3K27me3_BAM_NULL_2	H3K27me3	D.melanogaster	SRX017855	SRR038291	[18]
H3K27me3_CD4T_NULL_1_C	H3K27me3	M.musculus	SRX021636	SRR054679	[43]
H3K27me3_CD4T_NULL_2_C	H3K27me3	M.musculus	SRX021628	SRR054669	[43]
H3K27me3_CD4T_NULL_3_C	H3K27me3	M.musculus	SRX021628	SRR054670	[43]
H3K27me3_CD4T_S4KO_1_C	H3K27me3	M.musculus	SRX021629	SRR054671	[43]
H3K27me3_CD4T_S4KO_2_C	H3K27me3	M.musculus	SRX021629	SRR054672	[43]
H3K27me3_CD4T_S6KO_1_C	H3K27me3	M.musculus	SRX021637	SRR054680	[43]
H3K27me3_ES_NULL_1_C	H3K27me3	M.musculus	SRX018520	SRR038976	[38]
H3K27me3_ES_NULL_2_C	H3K27me3	M.musculus	SRX018519	SRR038975	[38]
H3K27me3_HESC_NULL_1	H3K27me3	H.sapiens	SRX027857	SRR067943	[9]
H3K27me3_HESC_NULL_10	H3K27me3	H.sapiens	SRX026074	SRR064676	[20]
H3K27me3_HESC_NULL_11	H3K27me3	H.sapiens	SRX026073	SRR064675	[20]
H3K27me3_HESC_NULL_12	H3K27me3	H.sapiens	SRX026072	SRR064674	[20]
H3K27me3_HESC_NULL_13	H3K27me3	H.sapiens	SRX026071	SRR064673	[20]
H3K27me3_HESC_NULL_14	H3K27me3	H.sapiens	SRX026070	SRR064669	[20]
H3K27me3_HESC_NULL_15	H3K27me3	H.sapiens	SRX026070	SRR064670	[20]
H3K27me3_HESC_NULL_16	H3K27me3	H.sapiens	SRX026070	SRR064671	[20]
H3K27me3_HESC_NULL_17	H3K27me3	H.sapiens	SRX026070	SRR064672	[20]
H3K27me3_HESC_NULL_2	H3K27me3	H.sapiens	SRX012370	SRR029345	[9]
H3K27me3_HESC_NULL_3	H3K27me3	H.sapiens	SRX012370	SRR029347	[9]
H3K27me3_HESC_NULL_4	H3K27me3	H.sapiens	SRX012369	SRR029343	[9]
H3K27me3_HESC_NULL_5	H3K27me3	H.sapiens	SRX012368	SRR029349	[9]
H3K27me3_HESC_NULL_6	H3K27me3	H.sapiens	SRX006874	SRR019561	[9]
H3K27me3_HESC_NULL_7	H3K27me3	H.sapiens	SRX027484	SRR067372	[36]
H3K27me3_HESC_NULL_8	H3K27me3	H.sapiens	SRX027456	SRR067320	n.a.
H3K27me3_HESC_NULL_9	H3K27me3	H.sapiens	SRX026075	SRR064677	[20]
H3K27me3_HFGM_NULL_1	H3K27me3	H.sapiens	SRX026069	SRR064668	[20]
H3K27me3_HeLa_NULL_1	H3K27me3	H.sapiens	SRX023664	SRR060537	[40]
H3K27me3_IMR90_NULL_1	H3K27me3	H.sapiens	SRX017508	SRR037555	[9]
H3K27me3_IMR90_NULL_2	H3K27me3	H.sapiens	SRX017508	SRR037560	[9]
H3K27me3_IMR90_NULL_3	H3K27me3	H.sapiens	SRX012498	SRR029611	[9]

Continued on next page

Table S1 – *Continued from previous page*

Experiment ID	Antibody target	Organism	SRA Experiment	SRA Run	Reference
H3K27me3_IMR90_NULL_4	H3K27me3	H.sapiens	SRX012498	SRR029616	[9]
H3K27me3_IMR90_NULL_5	H3K27me3	H.sapiens	SRX012498	SRR029623	[9]
H3K27me3_IMR90_NULL_6	H3K27me3	H.sapiens	SRX012498	SRR029627	[9]
H3K27me3_Jurkat_NULL_1	H3K27me3	H.sapiens	SRX024361	SRR061747	n.a.
H3K27me3_MDER10T_NULL_1	H3K27me3	M.musculus	SRX024352	SRR061738	n.a.
H3K27me3_MEF_0Div_1	H3K27me3	M.musculus	SRX035974	SRR087788	[29]
H3K27me3_MEF_1Div_1	H3K27me3	M.musculus	SRX035975	SRR087789	[29]
H3K27me3_MEF_2Div_1	H3K27me3	M.musculus	SRX035976	SRR087790	[29]
H3K27me3_MEF_3Div_1	H3K27me3	M.musculus	SRX035977	SRR087791	[29]
H3K27me3_MEF_NULL_1	H3K27me3	M.musculus	SRX027461	SRR067349	n.a.
H3K27me3_MEF_NULL_2	H3K27me3	M.musculus	SRX003803	SRR015023	[35]
H3K27me3_MEF_NULL_3	H3K27me3	M.musculus	SRX035973	SRR087787	[29]
H3K27me3_NEC_NULL_1	H3K27me3	H.sapiens	SRX027492	SRR067380	[36]
H3K27me3_S2_NULL_1	H3K27me3	D.melanogaster	SRX017850	SRR038285	[18]
H3K27me3_TCELL_NULL_1	H3K27me3	H.sapiens	SRX000143	SRR001426	[5]
H3K27me3_TCELL_NULL_2	H3K27me3	H.sapiens	SRX000143	SRR001427	[5]
H3K27me3_TCELL_NULL_3	H3K27me3	H.sapiens	SRX000143	SRR001428	[5]
H3K27me3_TCELL_NULL_4	H3K27me3	H.sapiens	SRX000143	SRR001429	[5]
H3K27me3_TSC_NULL_1_C	H3K27me3	M.musculus	SRX018523	SRR038979	[38]
H3K27me3_TSC_NULL_2_C	H3K27me3	M.musculus	SRX018522	SRR038978	[38]
H3K27me3_XEN_NULL_1_C	H3K27me3	M.musculus	SRX018527	SRR038983	[38]
H3K27me3_XEN_NULL_2_C	H3K27me3	M.musculus	SRX018526	SRR038982	[38]
H3K27me3_c2c12_MB_1	H3K27me3	M.musculus	SRX062110	SRR202874	[4]
H3K27me3_c2c12_MB_2	H3K27me3	M.musculus	SRX062110	SRR202875	[4]
H3K27me3_c2c12_MB_3	H3K27me3	M.musculus	SRX062110	SRR202876	[4]
H3K27me3_c2c12_MT_1	H3K27me3	M.musculus	SRX062111	SRR202877	[4]
H3K27me3_c2c12_MT_2	H3K27me3	M.musculus	SRX062111	SRR202878	[4]
H3K27me3_c2c12_MT_3	H3K27me3	M.musculus	SRX062111	SRR202879	[4]
H3K27me3_hiPS_NULL_1	H3K27me3	H.sapiens	SRX026082	SRR064684	[20]
H3K27me3_hiPS_NULL_2	H3K27me3	H.sapiens	SRX026080	SRR064682	[20]
H3K27me3_hiPS_NULL_3	H3K27me3	H.sapiens	SRX026079	SRR064681	[20]
H3K27me3_hiPS_NULL_4	H3K27me3	H.sapiens	SRX026078	SRR064680	[20]
H3K27me3_hiPS_NULL_5	H3K27me3	H.sapiens	SRX026077	SRR064679	[20]
H3K27me3_hiPS_NULL_6	H3K27me3	H.sapiens	SRX026076	SRR064678	[20]
H3K27me3_hiPS_PDB2lox_1	H3K27me3	H.sapiens	SRX026083	SRR064685	[20]
H3K27me3_hiPS_PDB2lox_2	H3K27me3	H.sapiens	SRX026081	SRR064683	[20]
H3K36me1_TCELL_NULL_1	H3K36me1	H.sapiens	SRX000144	SRR001397	[5]
H3K36me1_TCELL_NULL_2	H3K36me1	H.sapiens	SRX000144	SRR001406	[5]
H3K36me1_TCELL_NULL_3	H3K36me1	H.sapiens	SRX000144	SRR001407	[5]
H3K36me3_BAM_NULL_1	H3K36me3	D.melanogaster	SRX017856	SRR038292	[18]
H3K36me3_CD4T_NULL_1_C	H3K36me3	M.musculus	SRX021638	SRR054681	[43]
H3K36me3_CD4T_NULL_2_C	H3K36me3	M.musculus	SRX021630	SRR054673	[43]
H3K36me3_CD4T_S4KO_1_C	H3K36me3	M.musculus	SRX021631	SRR054674	[43]
H3K36me3_CD4T_S6KO_1_C	H3K36me3	M.musculus	SRX021639	SRR054682	[43]
H3K36me3_HESC_NULL_1	H3K36me3	H.sapiens	SRX027859	SRR067945	[9]
H3K36me3_HESC_NULL_2	H3K36me3	H.sapiens	SRX027858	SRR067944	[9]
H3K36me3_HESC_NULL_3	H3K36me3	H.sapiens	SRX012372	SRR029344	[9]
H3K36me3_HESC_NULL_4	H3K36me3	H.sapiens	SRX012372	SRR029346	[9]
H3K36me3_HESC_NULL_5	H3K36me3	H.sapiens	SRX012371	SRR029340	[9]
H3K36me3_HESC_NULL_6	H3K36me3	H.sapiens	SRX012371	SRR029341	[9]
H3K36me3_HESC_NULL_7	H3K36me3	H.sapiens	SRX012371	SRR029342	[9]
H3K36me3_HESC_NULL_8	H3K36me3	H.sapiens	SRX006235	SRR018454	[9]
H3K36me3_HeLa_NULL_1	H3K36me3	H.sapiens	SRX023666	SRR060539	[40]
H3K36me3_IMR90_NULL_1	H3K36me3	H.sapiens	SRX017511	SRR037562	[9]
H3K36me3_IMR90_NULL_2	H3K36me3	H.sapiens	SRX017510	SRR037546	[9]
H3K36me3_IMR90_NULL_3	H3K36me3	H.sapiens	SRX017510	SRR037550	[9]
H3K36me3_IMR90_NULL_4	H3K36me3	H.sapiens	SRX017510	SRR037553	[9]
H3K36me3_IMR90_NULL_5	H3K36me3	H.sapiens	SRX017509	SRR037592	[9]
H3K36me3_MEF_1Div_1	H3K36me3	M.musculus	SRX035985	SRR087799	[29]
H3K36me3_MEF_3Div_1	H3K36me3	M.musculus	SRX035986	SRR087800	[29]
H3K36me3_MEF_NULL_1	H3K36me3	M.musculus	SRX035984	SRR087798	[29]
H3K36me3_S2_NULL_1	H3K36me3	D.melanogaster	SRX017851	SRR038286	[18]
H3K36me3_TCELL_NULL_1	H3K36me3	H.sapiens	SRX000145	SRR001392	[5]
H3K36me3_TCELL_NULL_2	H3K36me3	H.sapiens	SRX000145	SRR001393	[5]
H3K36me3_TCELL_NULL_3	H3K36me3	H.sapiens	SRX000145	SRR001394	[5]
H3K36me3_TCELL_NULL_4	H3K36me3	H.sapiens	SRX000145	SRR001450	[5]
H3K36me3_c2c12_MB_1	H3K36me3	M.musculus	SRX062112	SRR202880	[4]
H3K36me3_c2c12_MB_2	H3K36me3	M.musculus	SRX062112	SRR202881	[4]
H3K36me3_c2c12_MB_3	H3K36me3	M.musculus	SRX062112	SRR202882	[4]
H3K36me3_c2c12_MT_1	H3K36me3	M.musculus	SRX062113	SRR202883	[4]
H3K36me3_c2c12_MT_2	H3K36me3	M.musculus	SRX062113	SRR202884	[4]
H3K36me3_c2c12_MT_3	H3K36me3	M.musculus	SRX062113	SRR202885	[4]
H3K4Ac_HESC_NULL_1	H3K4Ac	H.sapiens	SRX027860	SRR067946	[9]
H3K4Ac_IMR90_NULL_1	H3K4Ac	H.sapiens	SRX017513	SRR037595	[9]
H3K4Ac_IMR90_NULL_2	H3K4Ac	H.sapiens	SRX017513	SRR037596	[9]
H3K4Ac_IMR90_NULL_3	H3K4Ac	H.sapiens	SRX017512	SRR037593	[9]
H3K4Ac_IMR90_NULL_4	H3K4Ac	H.sapiens	SRX017512	SRR037594	[9]
H3K4me1_ES_NULL_1	H3K4me1	M.musculus	SRX027336	SRR066770	[14]
H3K4me1_ES_NULL_2	H3K4me1	M.musculus	SRX027335	SRR066771	[14]
H3K4me1_ES_NULL_3	H3K4me1	M.musculus	SRX027330	SRR066765	[14]
H3K4me1_ES_NULL_4	H3K4me1	M.musculus	SRX031218	SRR072987	n.a.
H3K4me1_HESC_NULL_1	H3K4me1	H.sapiens	SRX027861	SRR067947	[9]

Continued on next page

Table S1 – *Continued from previous page*

Experiment ID	Antibody target	Organism	SRA Experiment	SRA Run	Reference
H3K4me1_HESC_NULL_2	H3K4me1	H.sapiens	SRX012499	SRR029615	[9]
H3K4me1_HESC_NULL_3	H3K4me1	H.sapiens	SRX012499	SRR029619	[9]
H3K4me1_HESC_NULL_4	H3K4me1	H.sapiens	SRX012373	SRR029339	[9]
H3K4me1_HESC_NULL_5	H3K4me1	H.sapiens	SRX006236	SRR018456	[9]
H3K4me1_HESC_NULL_6	H3K4me1	H.sapiens	SRX027486	SRR067374	[36]
H3K4me1_IMR90_NULL_1	H3K4me1	H.sapiens	SRX017517	SRR037598	[9]
H3K4me1_IMR90_NULL_2	H3K4me1	H.sapiens	SRX017516	SRR037551	[9]
H3K4me1_IMR90_NULL_3	H3K4me1	H.sapiens	SRX017516	SRR037556	[9]
H3K4me1_IMR90_NULL_4	H3K4me1	H.sapiens	SRX017515	SRR037547	[9]
H3K4me1_IMR90_NULL_5	H3K4me1	H.sapiens	SRX017515	SRR037548	[9]
H3K4me1_IMR90_NULL_6	H3K4me1	H.sapiens	SRX017515	SRR037549	[9]
H3K4me1_IMR90_NULL_7	H3K4me1	H.sapiens	SRX017515	SRR037552	[9]
H3K4me1_IMR90_NULL_8	H3K4me1	H.sapiens	SRX017514	SRR037597	[9]
H3K4me1_MEF_1Div_1	H3K4me1	M.musculus	SRX035979	SRR087793	[29]
H3K4me1_MEF_3Div_1	H3K4me1	M.musculus	SRX035980	SRR087794	[29]
H3K4me1_MEF_NULL_1	H3K4me1	M.musculus	SRX027351	SRR066786	[14]
H3K4me1_MEF_NULL_2	H3K4me1	M.musculus	SRX027349	SRR066784	[14]
H3K4me1_MEF_NULL_3	H3K4me1	M.musculus	SRX035978	SRR087792	[29]
H3K4me1_NEC_NULL_1	H3K4me1	H.sapiens	SRX027494	SRR067382	[36]
H3K4me1_TCELL_NULL_1	H3K4me1	H.sapiens	SRX000146	SRR001439	[5]
H3K4me1_TCELL_NULL_2	H3K4me1	H.sapiens	SRX000146	SRR001444	[5]
H3K4me1_TCELL_NULL_3	H3K4me1	H.sapiens	SRX000146	SRR001445	[5]
H3K4me1_TCELL_NULL_4	H3K4me1	H.sapiens	SRX000146	SRR001446	[5]
H3K4me1_VCaP_NULL_1	H3K4me1	H.sapiens	SRX021803	SRR054892	[42]
H3K4me1_VCaP_NULL_2	H3K4me1	H.sapiens	SRX021803	SRR054894	[42]
H3K4me1_VCaP_NULL_3	H3K4me1	H.sapiens	SRX021803	SRR054895	[42]
H3K4me1_WLiver_NULL_1	H3K4me1	M.musculus	SRX027339	SRR066774	[14]
H3K4me1_c2c12_MB_1	H3K4me1	M.musculus	SRX062104	SRR202856	[4]
H3K4me1_c2c12_MB_2	H3K4me1	M.musculus	SRX062104	SRR202857	[4]
H3K4me1_c2c12_MB_3	H3K4me1	M.musculus	SRX062104	SRR202858	[4]
H3K4me1_c2c12_MT_1	H3K4me1	M.musculus	SRX062105	SRR202859	[4]
H3K4me1_c2c12_MT_2	H3K4me1	M.musculus	SRX062105	SRR202860	[4]
H3K4me1_c2c12_MT_3	H3K4me1	M.musculus	SRX062105	SRR202861	[4]
H3K4me1_proB_NULL_1	H3K4me1	M.musculus	SRX027344	SRR066779	[14]
H3K4me2_HESC_NULL_1	H3K4me2	H.sapiens	SRX027863	SRR067949	[9]
H3K4me2_HESC_NULL_2	H3K4me2	H.sapiens	SRX027862	SRR067948	[9]
H3K4me2_HESC_NULL_3	H3K4me2	H.sapiens	SRX027694	SRR067769	[9]
H3K4me2_HESC_NULL_4	H3K4me2	H.sapiens	SRX027693	SRR067768	[9]
H3K4me2_HeLa_NULL_1	H3K4me2	H.sapiens	SRX022431	SRR058521	[41]
H3K4me2_IMR90_NULL_1	H3K4me2	H.sapiens	SRX017519	SRR037554	[9]
H3K4me2_IMR90_NULL_2	H3K4me2	H.sapiens	SRX017519	SRR037558	[9]
H3K4me2_IMR90_NULL_3	H3K4me2	H.sapiens	SRX017518	SRR037599	[9]
H3K4me2_IMR90_NULL_4	H3K4me2	H.sapiens	SRX017518	SRR037600	[9]
H3K4me2_MEF_0Div_1	H3K4me2	M.musculus	SRX035969	SRR087783	[29]
H3K4me2_MEF_1Div_1	H3K4me2	M.musculus	SRX035970	SRR087784	[29]
H3K4me2_MEF_2Div_1	H3K4me2	M.musculus	SRX035971	SRR087785	[29]
H3K4me2_MEF_3Div_1	H3K4me2	M.musculus	SRX035972	SRR087786	[29]
H3K4me2_MEF_NULL_1	H3K4me2	M.musculus	SRX035968	SRR087782	[29]
H3K4me2_TCELL_NULL_1	H3K4me2	H.sapiens	SRX000147	SRR001449	[5]
H3K4me2_TCELL_NULL_2	H3K4me2	H.sapiens	SRX000147	SRR001465	[5]
H3K4me2_TCELL_NULL_3	H3K4me2	H.sapiens	SRX000147	SRR001466	[5]
H3K4me2_c2c12_MB_1	H3K4me2	M.musculus	SRX062106	SRR202862	[4]
H3K4me2_c2c12_MB_2	H3K4me2	M.musculus	SRX062106	SRR202863	[4]
H3K4me2_c2c12_MT_1	H3K4me2	M.musculus	SRX062107	SRR202864	[4]
H3K4me2_c2c12_MT_2	H3K4me2	M.musculus	SRX062107	SRR202865	[4]
H3K4me3_BAM_NULL_1	H3K4me3	D.melanogaster	SRX017854	SRR038289	[18]
H3K4me3_CD4T_NULL_1_C	H3K4me3	M.musculus	SRX021634	SRR054677	[43]
H3K4me3_CD4T_NULL_2_C	H3K4me3	M.musculus	SRX021626	SRR054667	[43]
H3K4me3_CD4T_NULL_3_C	H3K4me3	M.musculus	SRX020335	SRR043201	[15]
H3K4me3_CD4T_S3KO_1_C	H3K4me3	M.musculus	SRX020336	SRR043202	[15]
H3K4me3_CD4T_S4KO_1_C	H3K4me3	M.musculus	SRX021627	SRR054668	[43]
H3K4me3_CD4T_S6KO_1_C	H3K4me3	M.musculus	SRX021635	SRR054678	[43]
H3K4me3_ES_NULL_1	H3K4me3	M.musculus	SRX027334	SRR066769	[14]
H3K4me3_ES_NULL_1_C	H3K4me3	M.musculus	SRX018528	SRR038984	[38]
H3K4me3_ES_NULL_2_C	H3K4me3	M.musculus	SRX018518	SRR038974	[38]
H3K4me3_HESC_NULL_1	H3K4me3	H.sapiens	SRX027865	SRR067951	[9]
H3K4me3_HESC_NULL_10	H3K4me3	H.sapiens	SRX026057	SRR064656	[20]
H3K4me3_HESC_NULL_11	H3K4me3	H.sapiens	SRX026056	SRR064655	[20]
H3K4me3_HESC_NULL_12	H3K4me3	H.sapiens	SRX026055	SRR064651	[20]
H3K4me3_HESC_NULL_13	H3K4me3	H.sapiens	SRX026055	SRR064652	[20]
H3K4me3_HESC_NULL_14	H3K4me3	H.sapiens	SRX026055	SRR064653	[20]
H3K4me3_HESC_NULL_15	H3K4me3	H.sapiens	SRX026055	SRR064654	[20]
H3K4me3_HESC_NULL_2	H3K4me3	H.sapiens	SRX027864	SRR067950	[9]
H3K4me3_HESC_NULL_3	H3K4me3	H.sapiens	SRX012502	SRR029620	[9]
H3K4me3_HESC_NULL_4	H3K4me3	H.sapiens	SRX012501	SRR029609	[9]
H3K4me3_HESC_NULL_5	H3K4me3	H.sapiens	SRX006237	SRR018455	[9]
H3K4me3_HESC_NULL_6	H3K4me3	H.sapiens	SRX027487	SRR067375	[36]
H3K4me3_HESC_NULL_7	H3K4me3	H.sapiens	SRX026060	SRR064659	[20]
H3K4me3_HESC_NULL_8	H3K4me3	H.sapiens	SRX026059	SRR064658	[20]
H3K4me3_HESC_NULL_9	H3K4me3	H.sapiens	SRX026058	SRR064657	[20]
H3K4me3_HFGM_NULL_1	H3K4me3	H.sapiens	SRX026054	SRR064650	[20]
H3K4me3_HeLa_NULL_6	H3K4me3	H.sapiens	SRX023663	SRR060536	[40]

Continued on next page

Table S1 – *Continued from previous page*

Experiment ID	Antibody target	Organism	SRA Experiment	SRA Run	Reference
H3K4me3_IMR90_NULL_1	H3K4me3	H.sapiens	SRX017520	SRR037601	[9]
H3K4me3_IMR90_NULL_2	H3K4me3	H.sapiens	SRX017520	SRR037602	[9]
H3K4me3_IMR90_NULL_3	H3K4me3	H.sapiens	SRX012500	SRR029610	[9]
H3K4me3_IMR90_NULL_4	H3K4me3	H.sapiens	SRX012500	SRR029618	[9]
H3K4me3_Jurkat_NULL_1	H3K4me3	H.sapiens	SRX024357	SRR061743	n.a.
H3K4me3_MCF7_NULL_1	H3K4me3	H.sapiens	SRX016702	SRR036005	[17]
H3K4me3_MCF7_NULL_2	H3K4me3	H.sapiens	SRX016702	SRR036006	[17]
H3K4me3_MCF7_NULL_3	H3K4me3	H.sapiens	SRX016702	SRR036001	[17]
H3K4me3_MCF7_NULL_4	H3K4me3	H.sapiens	SRX016702	SRR036002	[17]
H3K4me3_MCF7_NULL_5	H3K4me3	H.sapiens	SRX016702	SRR036003	[17]
H3K4me3_MCF7_NULL_6	H3K4me3	H.sapiens	SRX016702	SRR036004	[17]
H3K4me3_MDER10T_NULL_1	H3K4me3	M.musculus	SRX024353	SRR061739	n.a.
H3K4me3_MEF_1Div_1	H3K4me3	M.musculus	SRX035982	SRR087796	[29]
H3K4me3_MEF_3Div_1	H3K4me3	M.musculus	SRX035983	SRR087797	[29]
H3K4me3_MEF_NULL_1	H3K4me3	M.musculus	SRX035981	SRR087795	[29]
H3K4me3_NEC_NULL_1	H3K4me3	H.sapiens	SRX027495	SRR067383	[36]
H3K4me3_S2_NULL_1	H3K4me3	D.melanogaster	SRX017849	SRR038283	[18]
H3K4me3_S2_NULL_2	H3K4me3	D.melanogaster	SRX017849	SRR038284	[18]
H3K4me3_S2_NULL_3	H3K4me3	D.melanogaster	SRX027833	SRR067916	[16]
H3K4me3_TCELL_NULL_1	H3K4me3	H.sapiens	SRX000148	SRR001414	[5]
H3K4me3_TCELL_NULL_2	H3K4me3	H.sapiens	SRX000148	SRR001419	[5]
H3K4me3_TCELL_NULL_3	H3K4me3	H.sapiens	SRX000148	SRR001420	[5]
H3K4me3_TCELL_NULL_4	H3K4me3	H.sapiens	SRX000148	SRR001421	[5]
H3K4me3_TCELL_NULL_5	H3K4me3	H.sapiens	SRX000148	SRR001430	[5]
H3K4me3_TCELL_NULL_6	H3K4me3	H.sapiens	SRX000148	SRR001431	[5]
H3K4me3_TCELL_NULL_7	H3K4me3	H.sapiens	SRX000148	SRR001432	[5]
H3K4me3_TSC_NULL_1_C	H3K4me3	M.musculus	SRX018521	SRR038977	[38]
H3K4me3_TSC_NULL_2_C	H3K4me3	M.musculus	SRX018517	SRR038973	[38]
H3K4me3_WLiver_NULL_1	H3K4me3	M.musculus	SRX027342	SRR066777	[14]
H3K4me3_XEN_NULL_1_C	H3K4me3	M.musculus	SRX018525	SRR038981	[38]
H3K4me3_XEN_NULL_2_C	H3K4me3	M.musculus	SRX018524	SRR038980	[38]
H3K4me3_c2c12_MB_1	H3K4me3	M.musculus	SRX062108	SRR202866	[4]
H3K4me3_c2c12_MB_2	H3K4me3	M.musculus	SRX062108	SRR202867	[4]
H3K4me3_c2c12_MB_3	H3K4me3	M.musculus	SRX062108	SRR202868	[4]
H3K4me3_c2c12_MB_4	H3K4me3	M.musculus	SRX062108	SRR202869	[4]
H3K4me3_c2c12_MT_1	H3K4me3	M.musculus	SRX062109	SRR202870	[4]
H3K4me3_c2c12_MT_2	H3K4me3	M.musculus	SRX062109	SRR202871	[4]
H3K4me3_c2c12_MT_3	H3K4me3	M.musculus	SRX062109	SRR202872	[4]
H3K4me3_c2c12_MT_4	H3K4me3	M.musculus	SRX062109	SRR202873	[4]
H3K4me3_hiPS_NULL_1	H3K4me3	H.sapiens	SRX026067	SRR064666	[20]
H3K4me3_hiPS_NULL_2	H3K4me3	H.sapiens	SRX026065	SRR064664	[20]
H3K4me3_hiPS_NULL_3	H3K4me3	H.sapiens	SRX026064	SRR064663	[20]
H3K4me3_hiPS_NULL_4	H3K4me3	H.sapiens	SRX026063	SRR064662	[20]
H3K4me3_hiPS_NULL_5	H3K4me3	H.sapiens	SRX026062	SRR064661	[20]
H3K4me3_hiPS_NULL_6	H3K4me3	H.sapiens	SRX026061	SRR064660	[20]
H3K4me3_hiPS_PDB2lox_1	H3K4me3	H.sapiens	SRX026068	SRR064667	[20]
H3K4me3_hiPS_PDB2lox_2	H3K4me3	H.sapiens	SRX026066	SRR064665	[20]
H3K4me3_proB_NULL_1	H3K4me3	M.musculus	SRX027347	SRR066782	[14]
H3K56Ac_HSEC_NULL_1	H3K56Ac	H.sapiens	SRX027866	SRR067952	[9]
H3K56Ac_IMR90_NULL_1	H3K56Ac	H.sapiens	SRX017522	SRR037605	[9]
H3K56Ac_IMR90_NULL_2	H3K56Ac	H.sapiens	SRX017521	SRR037603	[9]
H3K56Ac_IMR90_NULL_3	H3K56Ac	H.sapiens	SRX017521	SRR037604	[9]
H3K79me1_HESC_NULL_1	H3K79me1	H.sapiens	SRX027869	SRR067955	[9]
H3K79me1_HESC_NULL_2	H3K79me1	H.sapiens	SRX027868	SRR067954	[9]
H3K79me1_HESC_NULL_3	H3K79me1	H.sapiens	SRX027867	SRR067953	[9]
H3K79me1_IMR90_NULL_1	H3K79me1	H.sapiens	SRX017527	SRR037612	[9]
H3K79me1_IMR90_NULL_2	H3K79me1	H.sapiens	SRX017526	SRR037611	[9]
H3K79me1_IMR90_NULL_3	H3K79me1	H.sapiens	SRX017525	SRR037609	[9]
H3K79me1_IMR90_NULL_4	H3K79me1	H.sapiens	SRX017525	SRR037610	[9]
H3K79me1_IMR90_NULL_5	H3K79me1	H.sapiens	SRX017524	SRR037607	[9]
H3K79me1_IMR90_NULL_6	H3K79me1	H.sapiens	SRX017524	SRR037608	[9]
H3K79me1_IMR90_NULL_7	H3K79me1	H.sapiens	SRX017523	SRR037606	[9]
H3K79me1_TCELL_NULL_1	H3K79me1	H.sapiens	SRX000149	SRR001398	[5]
H3K79me1_TCELL_NULL_2	H3K79me1	H.sapiens	SRX000149	SRR001398	[5]
H3K79me1_TCELL_NULL_3	H3K79me1	H.sapiens	SRX000149	SRR001398	[5]
H3K79me2_HESC_NULL_1	H3K79me2	H.sapiens	SRX027871	SRR067957	[9]
H3K79me2_HESC_NULL_2	H3K79me2	H.sapiens	SRX027870	SRR067956	[9]
H3K79me2_HESC_NULL_3	H3K79me2	H.sapiens	SRX027457	SRR067321	n.a.
H3K79me2_IMR90_NULL_1	H3K79me2	H.sapiens	SRX017530	SRR037616	[9]
H3K79me2_IMR90_NULL_2	H3K79me2	H.sapiens	SRX017529	SRR037615	[9]
H3K79me2_IMR90_NULL_3	H3K79me2	H.sapiens	SRX017528	SRR037613	[9]
H3K79me2_IMR90_NULL_4	H3K79me2	H.sapiens	SRX017528	SRR037614	[9]
H3K79me2_TCELL_NULL_1	H3K79me2	H.sapiens	SRX000150	SRR001410	[5]
H3K79me3_HeLa_NULL_1	H3K79me3	H.sapiens	SRX023667	SRR060540	[40]
H3K79me3_MEF_NULL_1	H3K79me3	M.musculus	SRX022044	SRR057603	n.a.
H3K79me3_MEF_NULL_2	H3K79me3	M.musculus	SRX022043	SRR057602	n.a.
H3K79me3_TCELL_NULL_1	H3K79me3	H.sapiens	SRX000151	SRR001441	[5]
H3K79me3_TCELL_NULL_2	H3K79me3	H.sapiens	SRX000151	SRR001457	[5]
H3K79me3_TCELL_NULL_3	H3K79me3	H.sapiens	SRX000151	SRR001458	[5]
H3K9Ac_HESC_NULL_1	H3K9Ac	H.sapiens	SRX027873	SRR067959	[9]
H3K9Ac_HESC_NULL_2	H3K9Ac	H.sapiens	SRX027872	SRR067958	[9]
H3K9Ac_HeLa_NULL_1	H3K9Ac	H.sapiens	SRX023668	SRR060541	[40]

Continued on next page

Table S1 – *Continued from previous page*

Experiment ID	Antibody target	Organism	SRA Experiment	SRA Run	Reference
H3K9Ac_IMR90_NULL_1	H3K9Ac	H.sapiens	SRX017531	SRR037563	[9]
H3K9Ac_IMR90_NULL_2	H3K9Ac	H.sapiens	SRX012503	SRR029613	[9]
H3K9Ac_IMR90_NULL_3	H3K9Ac	H.sapiens	SRX012503	SRR029621	[9]
H3K9Ac_c2c12_MB_1	H3K9Ac	M.musculus	SRX062116	SRR202892	[4]
H3K9Ac_c2c12_MB_2	H3K9Ac	M.musculus	SRX062116	SRR202893	[4]
H3K9Ac_c2c12_MB_3	H3K9Ac	M.musculus	SRX062116	SRR202894	[4]
H3K9Ac_c2c12_MT_1	H3K9Ac	M.musculus	SRX062117	SRR202895	[4]
H3K9Ac_c2c12_MT_2	H3K9Ac	M.musculus	SRX062117	SRR202896	[4]
H3K9Ac_c2c12_MT_3	H3K9Ac	M.musculus	SRX062117	SRR202897	[4]
H3K9me1_TCELL_NULL_1	H3K9me1	H.sapiens	SRX000152	SRR001399	[5]
H3K9me1_TCELL_NULL_2	H3K9me1	H.sapiens	SRX000152	SRR001400	[5]
H3K9me1_TCELL_NULL_3	H3K9me1	H.sapiens	SRX000152	SRR001454	[5]
H3K9me1_TCELL_NULL_4	H3K9me1	H.sapiens	SRX000152	SRR001467	[5]
H3K9me2_DMLarvae_EHMTKO_1	H3K9me2	D.melanogaster	SRX022335	SRR058396	[30]
H3K9me2_DMLarvae_NULL_1	H3K9me2	D.melanogaster	SRX022334	SRR058395	[30]
H3K9me2_TCELL_NULL_1	H3K9me2	H.sapiens	SRX000153	SRR001440	[5]
H3K9me2_TCELL_NULL_2	H3K9me2	H.sapiens	SRX000153	SRR001455	[5]
H3K9me2_TCELL_NULL_3	H3K9me2	H.sapiens	SRX000153	SRR001456	[5]
H3K9me3_HESC_NULL_1	H3K9me3	H.sapiens	SRX027877	SRR067965	[9]
H3K9me3_HESC_NULL_2	H3K9me3	H.sapiens	SRX027876	SRR067964	[9]
H3K9me3_HESC_NULL_3	H3K9me3	H.sapiens	SRX027875	SRR067962	[9]
H3K9me3_HESC_NULL_4	H3K9me3	H.sapiens	SRX027875	SRR067963	[9]
H3K9me3_HESC_NULL_5	H3K9me3	H.sapiens	SRX027874	SRR067960	[9]
H3K9me3_HESC_NULL_6	H3K9me3	H.sapiens	SRX027874	SRR067961	[9]
H3K9me3_HESC_NULL_7	H3K9me3	H.sapiens	SRX006875	SRR019562	[9]
H3K9me3_HESC_NULL_8	H3K9me3	H.sapiens	SRX006238	SRR018453	[9]
H3K9me3_HeLa_NULL_1	H3K9me3	H.sapiens	SRX023665	SRR060538	[40]
H3K9me3_IMR90_NULL_1	H3K9me3	H.sapiens	SRX017533	SRR037619	[9]
H3K9me3_IMR90_NULL_2	H3K9me3	H.sapiens	SRX017532	SRR037617	[9]
H3K9me3_IMR90_NULL_3	H3K9me3	H.sapiens	SRX017532	SRR037618	[9]
H3K9me3_IMR90_NULL_4	H3K9me3	H.sapiens	SRX012504	SRR029612	[9]
H3K9me3_IMR90_NULL_5	H3K9me3	H.sapiens	SRX012504	SRR029617	[9]
H3K9me3_IMR90_NULL_6	H3K9me3	H.sapiens	SRX012504	SRR029624	[9]
H3K9me3_IMR90_NULL_7	H3K9me3	H.sapiens	SRX012504	SRR029628	[9]
H3K9me3_MDER10T_NULL_1	H3K9me3	M.musculus	SRX024354	SRR061740	n.a.
H3K9me3_TCELL_NULL_1	H3K9me3	H.sapiens	SRX000154	SRR001422	[5]
H3K9me3_TCELL_NULL_2	H3K9me3	H.sapiens	SRX000154	SRR001423	[5]
H3K9me3_TCELL_NULL_3	H3K9me3	H.sapiens	SRX000154	SRR001424	[5]
H3K9me3_TCELL_NULL_4	H3K9me3	H.sapiens	SRX000154	SRR001425	[5]
H3R2me1_TCELL_NULL_1	H3R2me1	H.sapiens	SRX000155	SRR001395	[5]
H3R2me1_TCELL_NULL_2	H3R2me1	H.sapiens	SRX000155	SRR001396	[5]
H3R2me1_TCELL_NULL_3	H3R2me1	H.sapiens	SRX000155	SRR001453	[5]
H3R2me2_TCELL_NULL_1	H3R2me2	H.sapiens	SRX000156	SRR001411	[5]
H3R2me2_TCELL_NULL_2	H3R2me2	H.sapiens	SRX000156	SRR001443	[5]
H4K12Ac_c2c12_MB_1	H4K12Ac	M.musculus	SRX062120	SRR202904	[4]
H4K12Ac_c2c12_MB_2	H4K12Ac	M.musculus	SRX062120	SRR202905	[4]
H4K12Ac_c2c12_MB_3	H4K12Ac	M.musculus	SRX062120	SRR202906	[4]
H4K12Ac_c2c12_MB_4	H4K12Ac	M.musculus	SRX062120	SRR202907	[4]
H4K12Ac_c2c12_MT_1	H4K12Ac	M.musculus	SRX062121	SRR202908	[4]
H4K12Ac_c2c12_MT_2	H4K12Ac	M.musculus	SRX062121	SRR202909	[4]
H4K12Ac_c2c12_MT_3	H4K12Ac	M.musculus	SRX062121	SRR202910	[4]
H4K12Ac_c2c12_MT_4	H4K12Ac	M.musculus	SRX062121	SRR202911	[4]
H4K20me1_HESC_NULL_1	H4K20me1	H.sapiens	SRX027878	SRR067966	[9]
H4K20me1_HeLa_NULL_1	H4K20me1	H.sapiens	SRX022435	SRR058525	[41]
H4K20me1_IMR90_NULL_1	H4K20me1	H.sapiens	SRX017536	SRR037623	[9]
H4K20me1_IMR90_NULL_2	H4K20me1	H.sapiens	SRX017535	SRR037621	[9]
H4K20me1_IMR90_NULL_3	H4K20me1	H.sapiens	SRX017535	SRR037622	[9]
H4K20me1_IMR90_NULL_4	H4K20me1	H.sapiens	SRX017534	SRR037620	[9]
H4K20me1_TCELL_NULL_1	H4K20me1	H.sapiens	SRX000157	SRR001402	[5]
H4K20me1_TCELL_NULL_2	H4K20me1	H.sapiens	SRX000157	SRR001403	[5]
H4K20me1_TCELL_NULL_3	H4K20me1	H.sapiens	SRX000157	SRR001452	[5]
H4K20me3_TCELL_NULL_1	H4K20me3	H.sapiens	SRX000158	SRR001404	[5]
H4K20me3_TCELL_NULL_2	H4K20me3	H.sapiens	SRX000158	SRR001405	[5]
H4K20me3_TCELL_NULL_3	H4K20me3	H.sapiens	SRX000158	SRR001451	[5]
H4K5Ac_HESC_NULL_1	H4K5Ac	H.sapiens	SRX027880	SRR067968	[9]
H4K5Ac_HESC_NULL_2	H4K5Ac	H.sapiens	SRX027879	SRR067967	[9]
H4K5Ac_IMR90_NULL_1	H4K5Ac	H.sapiens	SRX017537	SRR037624	[9]
H4K5Ac_IMR90_NULL_2	H4K5Ac	H.sapiens	SRX017537	SRR037625	[9]
H4K5Ac_IMR90_NULL_3	H4K5Ac	H.sapiens	SRX012505	SRR029614	[9]
H4K5Ac_IMR90_NULL_4	H4K5Ac	H.sapiens	SRX012505	SRR029622	[9]
H4K8Ac_IMR90_NULL_1	H4K8Ac	H.sapiens	SRX017542	SRR037630	[9]
H4K8Ac_IMR90_NULL_2	H4K8Ac	H.sapiens	SRX017541	SRR037629	[9]
H4K8Ac_IMR90_NULL_3	H4K8Ac	H.sapiens	SRX017540	SRR037628	[9]
H4K8Ac_IMR90_NULL_4	H4K8Ac	H.sapiens	SRX017539	SRR037627	[9]
H4K8Ac_IMR90_NULL_5	H4K8Ac	H.sapiens	SRX017538	SRR037626	[9]
H4K91Ac_HESC_NULL_1	H4K91Ac	H.sapiens	SRX027881	SRR067969	[9]
H4K91Ac_IMR90_NULL_1	H4K91Ac	H.sapiens	SRX017544	SRR037633	[9]
H4K91Ac_IMR90_NULL_2	H4K91Ac	H.sapiens	SRX017543	SRR037631	[9]
H4K91Ac_IMR90_NULL_3	H4K91Ac	H.sapiens	SRX017543	SRR037632	[9]
H4R3me2_TCELL_NULL_1	H4R3me2	H.sapiens	SRX000159	SRR001415	[5]
H4R3me2_TCELL_NULL_2	H4R3me2	H.sapiens	SRX000159	SRR001416	[5]
H4R3me2_TCELL_NULL_3	H4R3me2	H.sapiens	SRX000159	SRR001417	[5]

Continued on next page

Table S1 – *Continued from previous page*

Experiment ID	Antibody target	Organism	SRA Experiment	SRA Run	Reference
H4R3me2_TCELL_NULL_4	H4R3me2	H.sapiens	SRX000159	SRR001418	[5]
hERa_MCF7_NULL_1	hERa	H.sapiens	ERX004472	ERR011973	[39]
hERa_MCF7_NULL_2	hERa	H.sapiens	ERX004457	ERR011978	[39]
hERa_MCF7_NULL_3	hERa	H.sapiens	ERX008576	ERR022053	[22]
hERa_MCF7_NULL_4	hERa	H.sapiens	ERX008575	ERR022018	[22]
hERa_MCF7_NULL_5	hERa	H.sapiens	ERX008572	ERR022052	[22]
hERa_MCF7_NULL_6	hERa	H.sapiens	ERX008617	ERR022019	[22]
hERa_MCF7_NULL_7	hERa	H.sapiens	ERX008603	ERR022057	[22]
hERa_T47D_NULL_1	hERa	H.sapiens	ERX008586	ERR022011	[22]
hERa_T47D_NULL_2	hERa	H.sapiens	ERX008580	ERR022006	[22]
hERa_T47D_NULL_3	hERa	H.sapiens	ERX008615	ERR022005	[22]
hERa_T47D_NULL_4	hERa	H.sapiens	ERX008610	ERR022007	[22]
hERa_T47D_NULL_5	hERa	H.sapiens	ERX008607	ERR022041	[22]
hERa_ZR75_NULL_1	hERa	H.sapiens	ERX008594	ERR022037	[22]
hERa_ZR75_NULL_2	hERa	H.sapiens	ERX008592	ERR022010	[22]
hERa_ZR75_NULL_3	hERa	H.sapiens	ERX008583	ERR022009	[22]
hERa_ZR75_NULL_4	hERa	H.sapiens	ERX008569	ERR022015	[22]
hERa_ZR75_NULL_5	hERa	H.sapiens	ERX008613	ERR022008	[22]
hERa_ZR75_NULL_6	hERa	H.sapiens	ERX008604	ERR022014	[22]
HNF4A_CACO2_DIFF_1	HNF4A	H.sapiens	SRX026268	SRR064952	[41]
HNF4A_CACO2_PROL_1	HNF4A	H.sapiens	SRX026267	SRR064951	[41]
HNF4A_HEPG2_NULL_1	HNF4A	H.sapiens	ERX004453	ERR011992	[39]
HOXC9_Ainv15_NULL_1_C	HOXC9	M.musculus	SRX021369	SRR051942	[26]
HOXC9_Ainv15_NULL_2_C	HOXC9	M.musculus	SRX021369	SRR051943	[26]
IgG_CADO_NULL_1_C	IgG	H.sapiens	SRX021785	SRR054856	[42]
IgG_CADO_NULL_2_C	IgG	H.sapiens	SRX021785	SRR054857	[42]
IgG_GM12878_NULL_1_C	IgG	H.sapiens	SRX017998	SRR038401	[28]
IgG_GM12878_NULL_2_C	IgG	H.sapiens	SRX017991	SRR038394	[28]
IgG_GM12878_NULL_3_C	IgG	H.sapiens	SRX017990	SRR038393	[28]
IgG_GM12891_NULL_1_C	IgG	H.sapiens	SRX017999	SRR038402	[28]
IgG_GM18526_NULL_1_C	IgG	H.sapiens	SRX017982	SRR038380	[28]
IgG_GM18526_NULL_2_C	IgG	H.sapiens	SRX017982	SRR038381	[28]
IgG_GM18526_NULL_3_C	IgG	H.sapiens	SRX017982	SRR038382	[28]
IgG_GM18526_NULL_4_C	IgG	H.sapiens	SRX017982	SRR038383	[28]
IgG_GM18526_NULL_5_C	IgG	H.sapiens	SRX017981	SRR038379	[28]
IgG_GM18526_NULL_6_C	IgG	H.sapiens	SRX017980	SRR038378	[28]
IgG_GM18951_NULL_1_C	IgG	H.sapiens	SRX017976	SRR038374	[28]
IgG_GM18951_NULL_2_C	IgG	H.sapiens	SRX017975	SRR038371	[28]
IgG_GM18951_NULL_3_C	IgG	H.sapiens	SRX017975	SRR038372	[28]
IgG_GM18951_NULL_4_C	IgG	H.sapiens	SRX017975	SRR038373	[28]
IgG_HESC_NULL_1_C	IgG	H.sapiens	SRX021068	SRR049932	n.a.
IgG_HL60_NULL_1_C	IgG	H.sapiens	SRX021787	SRR057328	[42]
IgG_HSC_NULL_1	IgG	M.musculus	SRX035993	SRR087807	[32]
IgG_HSC_NULL_2	IgG	M.musculus	SRX035993	SRR087808	[32]
IgG_Jurkat_NULL_1	IgG	H.sapiens	SRX021789	SRR054860	[42]
IgG_MCF7_NULL_1	IgG	H.sapiens	SRX022742	SRR059121	[44]
IgG_SKNMC_NULL_1	IgG	H.sapiens	SRX021796	SRR054873	[42]
IgG_SKNMC_NULL_2	IgG	H.sapiens	SRX021796	SRR054872	[42]
IgG_SKNMC_NULL_3	IgG	H.sapiens	SRX021795	SRR054871	[42]
IgG_VCaP_NULL_1	IgG	H.sapiens	SRX021806	SRR054907	[42]
IgG_VCaP_NULL_2	IgG	H.sapiens	SRX021804	SRR054896	[42]
IgG_VCaP_NULL_3	IgG	H.sapiens	SRX021804	SRR054897	[42]
IgG_VCaP_NULL_4	IgG	H.sapiens	SRX021800	SRR054877	[42]
IgG_VCaP_NULL_5	IgG	H.sapiens	SRX021800	SRR054878	[42]
IgG_VCaP_NULL_6	IgG	H.sapiens	SRX021800	SRR054879	[42]
LDB1_HSC_NULL_1	LDB1	M.musculus	SRX035990	SRR087804	[32]
LRWD1_HeLa_NULL_1	LRWD1	H.sapiens	SRX023661	SRR060534	[40]
MCRS2_SalGlan_NULL_1	MCRS2	D.melanogaster	ERX004310	ERR011354	[32]
MED1_ES_NULL_1	MED1	M.musculus	SRX022695	SRR058988	[27]
MED1_ES_NULL_2	MED1	M.musculus	SRX022694	SRR058987	[27]
MED1_MEF_NULL_1	MED1	M.musculus	SRX022700	SRR058993	[27]
MED12_ES_NULL_1	MED12	M.musculus	SRX022692	SRR058985	[27]
MED12_MEF_NULL_1	MED12	M.musculus	SRX022701	SRR058994	[27]
MED12_MEF_NULL_2	MED12	M.musculus	SRX022693	SRR058986	[27]
MSL3TAP_Dmel_NULL_1	MSL3TAP	D.melanogaster	SRX011651	SRR027999	[19]
NF1_MEF_NFICKO_1	NF1	M.musculus	SRX017080	SRR036759	n.a.
NF1_MEF_WT_1	NF1	M.musculus	SRX017079	SRR036758	n.a.
NFATC1_SELEX_NULL_1_C	NFATC1	H.sapiens	SRX017448	SRR037643	[25]
NFATC1_SELEX_NULL_2_C	NFATC1	H.sapiens	SRX017447	SRR037544	[25]
NIPBL_ES_NULL_1	NIPBL	M.musculus	SRX022697	SRR058990	[27]
NIPBL_ES_NULL_2	NIPBL	M.musculus	SRX022696	SRR058989	[27]
NSL1_SalGlan_NULL_1	NSL1	D.melanogaster	ERX004312	ERR011355	[32]
NULL_CD4T_NULL_1_C	NULL	M.musculus	SRX021640	SRR054683	[43]
NULL_CD4T_S4KO_1_C	NULL	M.musculus	SRX021625	SRR054666	[43]
NULL_CD4T_S6KO_1_C	NULL	M.musculus	SRX021633	SRR054676	[43]
NULL_HeLa_NULL_1	NULL	H.sapiens	SRX023662	SRR060535	[40]
OCT4_HESC_NULL_1_C	OCT4	H.sapiens	SRX021071	SRR049935	n.a.
OCT4_HESC_NULL_2_C	OCT4	H.sapiens	SRX021070	SRR049934	n.a.
OCT4_HESC_NULL_3_C	OCT4	H.sapiens	SRX021069	SRR049933	n.a.
P130_QuiFib_NULL_1	P130	H.sapiens	SRX016036	SRR034489	[13]
P130_QuiFib_NULL_2	P130	H.sapiens	SRX016031	SRR034483	[13]
P130_SenFib_NULL_1	P130	H.sapiens	SRX016038	SRR034491	[13]

Continued on next page

Table S1 – *Continued from previous page*

Experiment ID	Antibody target	Organism	SRA Experiment	SRA Run	Reference
P130_SenFib_NULL_2	P130	H.sapiens	SRX016034	SRR034487	[13]
P300_CD1_BRAIN_1	P300	M.musculus	SRX022495	SRR058612	[11]
P300_CD1_HEART_1	P300	M.musculus	SRX022494	SRR058611	[11]
P300_ES_NULL_1	P300	M.musculus	SRX027354	SRR066789	[14]
P300_ES_NULL_2	P300	M.musculus	SRX027353	SRR066788	[14]
P300_HESC_NULL_1	P300	H.sapiens	SRX027482	SRR067370	[36]
P300_NEC_NULL_1	P300	H.sapiens	SRX027490	SRR067378	[36]
PC_S2_NULL_1	PC	D.melanogaster	SRX027824	SRR067907	[16]
PC_S2_NULL_2	PC	D.melanogaster	SRX027823	SRR067906	[16]
PH_S2_NULL_1	PH	D.melanogaster	SRX027826	SRR067909	[16]
PH_S2_NULL_2	PH	D.melanogaster	SRX027825	SRR067908	[16]
PHF8_HeLa_NULL_1	PHF8	H.sapiens	SRX022433	SRR058523	[41]
PHF8_HeLa_NULL_2	PHF8	H.sapiens	SRX023659	SRR060532	[40]
PHF8_HeLa_NULL_3	PHF8	H.sapiens	SRX023653	SRR060526	[40]
PolII_BAM_NULL_1	PolII	D.melanogaster	SRX017857	SRR038293	[18]
PolII_ES_S7P_1_C	PolII	M.musculus	SRX021357	SRR051928	n.a.
PolII_GM12878_NULL_1_C	PolII	H.sapiens	SRX017989	SRR038391	[28]
PolII_GM12878_NULL_2_C	PolII	H.sapiens	SRX017989	SRR038392	[28]
PolII_GM12878_NULL_3_C	PolII	H.sapiens	SRX017988	SRR038389	[28]
PolII_GM12878_NULL_4_C	PolII	H.sapiens	SRX017988	SRR038390	[28]
PolII_GM12878_NULL_5_C	PolII	H.sapiens	SRX017987	SRR038388	[28]
PolII_GM12878_NULL_6_C	PolII	H.sapiens	SRX017986	SRR038387	[28]
PolII_GM12878_NULL_7_C	PolII	H.sapiens	SRX017985	SRR038386	[28]
PolII_GM12878_NULL_8_C	PolII	H.sapiens	SRX017984	SRR038385	[28]
PolII_GM12878_NULL_9_C	PolII	H.sapiens	SRX017983	SRR038384	[28]
PolII_GM12891_NULL_1_C	PolII	H.sapiens	SRX017997	SRR038400	[28]
PolII_GM12891_NULL_2_C	PolII	H.sapiens	SRX017996	SRR038399	[28]
PolII_GM12891_NULL_3_C	PolII	H.sapiens	SRX017995	SRR038398	[28]
PolII_GM12891_NULL_4_C	PolII	H.sapiens	SRX017994	SRR038397	[28]
PolII_GM12891_NULL_5_C	PolII	H.sapiens	SRX017993	SRR038396	[28]
PolII_GM12891_NULL_6_C	PolII	H.sapiens	SRX017992	SRR038395	[28]
PolII_GM18526_NULL_1_C	PolII	H.sapiens	SRX017979	SRR038377	[28]
PolII_GM18526_NULL_2_C	PolII	H.sapiens	SRX017978	SRR038376	[28]
PolII_GM18526_NULL_3_C	PolII	H.sapiens	SRX017977	SRR038375	[28]
PolII_GM18951_NULL_1_C	PolII	H.sapiens	SRX017974	SRR038370	[28]
PolII_GM18951_NULL_2_C	PolII	H.sapiens	SRX017973	SRR038368	[28]
PolII_GM18951_NULL_3_C	PolII	H.sapiens	SRX017973	SRR038369	[28]
PolII_GM18951_NULL_4_C	PolII	H.sapiens	SRX017972	SRR038366	[28]
PolII_GM18951_NULL_5_C	PolII	H.sapiens	SRX017972	SRR038367	[28]
PolII_GM18951_NULL_6_C	PolII	H.sapiens	SRX017971	SRR038364	[28]
PolII_GM18951_NULL_7_C	PolII	H.sapiens	SRX017971	SRR038365	[28]
PolII_HBG3_S2P_1	PolII	M.musculus	SRX033239	SRR077870	[33]
PolII_HBG3_S2P_2	PolII	M.musculus	SRX033239	SRR077871	[33]
PolII_Jurkat_10DRB_1	PolII	H.sapiens	SRX033328	SRR080727	[23]
PolII_Jurkat_25DRB_1	PolII	H.sapiens	SRX033329	SRR080728	[23]
PolII_Jurkat_DMSO_1	PolII	H.sapiens	SRX033327	SRR080726	[23]
PolII_Jurkat_NULL_1	PolII	H.sapiens	SRX024360	SRR061746	n.a.
PolII_Jurkat_NULL_2	PolII	H.sapiens	SRX024359	SRR061745	n.a.
PolII_MCF7_NULL_1	PolII	H.sapiens	SRX016705	SRR036012	[17]
PolII_MCF7_NULL_2	PolII	H.sapiens	SRX016705	SRR036013	[17]
PolII_MCF7_NULL_3	PolII	H.sapiens	SRX016705	SRR036014	[17]
PolII_MCF7_NULL_4	PolII	H.sapiens	SRX016704	SRR036009	[17]
PolII_MCF7_NULL_5	PolII	H.sapiens	SRX016704	SRR036010	[17]
PolII_MCF7_NULL_6	PolII	H.sapiens	SRX016704	SRR036011	[17]
PolII_MEF_NULL_1	PolII	M.musculus	SRX027462	SRR067350	n.a.
PolII_MEF_NULL_2	PolII	M.musculus	SRX022046	SRR057605	n.a.
PolII_MEF_NULL_3	PolII	M.musculus	SRX022045	SRR057604	n.a.
PolII_S2_NULL_1	PolII	D.melanogaster	SRX017852	SRR038287	[18]
PolII_TCELL_NULL_1	PolII	H.sapiens	SRX000160	SRR001437	[5]
PolII_TCELL_NULL_2	PolII	H.sapiens	SRX000160	SRR001447	[5]
PolII_TCELL_NULL_3	PolII	H.sapiens	SRX000160	SRR001461	[5]
PolII_TCELL_NULL_4	PolII	H.sapiens	SRX000160	SRR001462	[5]
PolII_c2c12_MB_1	PolII	M.musculus	SRX062102	SRR202850	[4]
PolII_c2c12_MB_2	PolII	M.musculus	SRX062102	SRR202851	[4]
PolII_c2c12_MB_3	PolII	M.musculus	SRX062102	SRR202852	[4]
PolII_c2c12_MT_1	PolII	M.musculus	SRX062103	SRR202853	[4]
PolII_c2c12_MT_2	PolII	M.musculus	SRX062103	SRR202854	[4]
PolII_c2c12_MT_3	PolII	M.musculus	SRX062103	SRR202855	[4]
pRb_GroFib_NULL_1	pRb	H.sapiens	SRX016028	SRR034478	[13]
pRb_GroFib_NULL_2	pRb	H.sapiens	SRX016028	SRR034479	[13]
pRb_QuiFib_NULL_1	pRb	H.sapiens	SRX016035	SRR034488	[13]
pRb_QuiFib_NULL_2	pRb	H.sapiens	SRX016030	SRR034482	[13]
pRb_QuiFib_NULL_3	pRb	H.sapiens	SRX016029	SRR034480	[13]
pRb_QuiFib_NULL_4	pRb	H.sapiens	SRX016029	SRR034481	[13]
pRb_SenFib_NULL_1	pRb	H.sapiens	SRX016037	SRR034490	[13]
pRb_SenFib_NULL_2	pRb	H.sapiens	SRX016033	SRR034486	[13]
pRb_SenFib_NULL_3	pRb	H.sapiens	SRX016032	SRR034484	[13]
pRb_SenFib_NULL_4	pRb	H.sapiens	SRX016032	SRR034485	[13]
Prdm14_ES_NULL_1	Prdm14	M.musculus	SRX031215	SRR072984	n.a.
PSC_KC_NULL_1	PSC	D.melanogaster	SRX018162	SRR038619	[10]
PSC_KC_NULL_2	PSC	D.melanogaster	SRX018161	SRR038618	[10]
PSC_KC_NULL_3	PSC	D.melanogaster	SRX018160	SRR038617	[10]

Continued on next page

Table S1 – *Continued from previous page*

Experiment ID	Antibody target	Organism	SRA Experiment	SRA Run	Reference
PSC_S2_NULL_1	PSC	D.melanogaster	SRX027828	SRR067911	[16]
PSC_S2_NULL_2	PSC	D.melanogaster	SRX027827	SRR067910	[16]
RAD21_HEPG2_NULL_1	RAD21	H.sapiens	ERX004454	ERR011985	[39]
RAD21_HEPG2_NULL_2	RAD21	H.sapiens	ERX004450	ERR011986	[39]
RAD21_MCF7_ER_1	RAD21	H.sapiens	ERX004467	ERR011977	[39]
RAD21_MCF7_ER_2	RAD21	H.sapiens	ERX004455	ERR011976	[39]
RAD21_MCF7_NULL_1	RAD21	H.sapiens	ERX004452	ERR011981	[39]
RAD21_MCF7_NULL_2	RAD21	H.sapiens	ERX004471	ERR011982	[39]
RAR_HBG3_NULL_1_C	RAR	M.musculus	SRX018669	SRR039155	[33]
RAR_HBG3_NULL_2_C	RAR	M.musculus	SRX018669	SRR039156	[33]
RAR_HBG3_NULL_3_C	RAR	M.musculus	SRX018669	SRR039157	[33]
RAR_HBG3_NULL_4_C	RAR	M.musculus	SRX018669	SRR039158	[33]
RAR_HBG3_NULL_5_C	RAR	M.musculus	SRX018669	SRR039159	[33]
RAR_HBG3_NULL_6_C	RAR	M.musculus	SRX018669	SRR039160	[33]
RAR_HBG3_RA_1_C	RAR	M.musculus	SRX018670	SRR039161	[33]
RAR_HBG3_RA_2_C	RAR	M.musculus	SRX018670	SRR039162	[33]
RAR_HBG3_RA_3_C	RAR	M.musculus	SRX018670	SRR039163	[33]
RAR_HBG3_RA_4_C	RAR	M.musculus	SRX018670	SRR039164	[33]
RAR_HBG3_RA_5_C	RAR	M.musculus	SRX018670	SRR039165	[33]
RAR_HBG3_RA_6_C	RAR	M.musculus	SRX018670	SRR039166	[33]
RFX1_NeuProg_NULL_1	RFX1	M.musculus	SRX027337	SRR066772	[14]
RPA116_HEK293T_NULL_1	RPA116	H.sapiens	SRX035784	SRR087747	n.a.
SCLTAL1_HSC_NULL_1	SCLTAL1	M.musculus	SRX035991	SRR087805	[32]
SGF29_HeLa_NULL_1	SGF29	H.sapiens	SRX023650	SRR060523	[40]
SGF29_HeLa_NULL_2	SGF29	H.sapiens	SRX023656	SRR060529	[40]
SMC1_ES_NULL_1	SMC1	M.musculus	SRX022689	SRR058982	[27]
SMC1_ES_NULL_2	SMC1	M.musculus	SRX022688	SRR058981	[27]
SMC1_MEF_NULL_1	SMC1	M.musculus	SRX022703	SRR058996	[27]
SMC1_MEF_NULL_2	SMC1	M.musculus	SRX022702	SRR058995	[27]
SMC3_ES_NULL_1	SMC3	M.musculus	SRX022691	SRR058984	[27]
SMC3_ES_NULL_2	SMC3	M.musculus	SRX022690	SRR058983	[27]
SMC4_HeLa_NULL_1	SMC4	H.sapiens	SRX022434	SRR058524	[41]
SOX2_ES_NULL_1	SOX2	M.musculus	SRX020915	SRR050356	[34]
SPDEF_VCaP_NULL_1	SPDEF	H.sapiens	SRX021805	SRR054898	[42]
SPDEF_VCaP_NULL_2	SPDEF	H.sapiens	SRX021805	SRR054899	[42]
SPDEF_VCaP_NULL_3	SPDEF	H.sapiens	SRX021805	SRR054900	[42]
SPDEF_VCaP_NULL_4	SPDEF	H.sapiens	SRX021805	SRR054901	[42]
SPDEF_VCaP_NULL_5	SPDEF	H.sapiens	SRX021805	SRR054902	[42]
SPDEF_VCaP_NULL_6	SPDEF	H.sapiens	SRX021805	SRR054903	[42]
SPDEF_VCaP_NULL_7	SPDEF	H.sapiens	SRX021805	SRR054904	[42]
SPDEF_VCaP_NULL_8	SPDEF	H.sapiens	SRX021805	SRR054905	[42]
SPDEF_VCaP_NULL_9	SPDEF	H.sapiens	SRX021805	SRR054906	[42]
SPI1_HL60_NULL_1	SPI1	H.sapiens	SRX021786	SRR054858	[42]
STAG1_HEPG2_NULL_1	STAG1	H.sapiens	ERX004474	ERR011983	[39]
STAG1_HEPG2_NULL_2	STAG1	H.sapiens	ERX004464	ERR011984	[39]
STAG1_MCF7_ER_1	STAG1	H.sapiens	ERX004463	ERR011975	[39]
STAG1_MCF7_NULL_1	STAG1	H.sapiens	ERX004477	ERR011974	[39]
STAG1_MCF7_NULL_2	STAG1	H.sapiens	ERX004473	ERR011979	[39]
STAG1_MCF7_NULL_3	STAG1	H.sapiens	ERX004468	ERR011980	[39]
STAT3_CD4T_NULL_1_C	STAT3	M.musculus	SRX020337	SRR043203	[15]
STAT4_CD4T_NULL_1_C	STAT4	M.musculus	SRX021624	SRR054665	[43]
STAT6_CD4T_NULL_1_C	STAT6	M.musculus	SRX021632	SRR054675	[43]
SUZ12_HESC_NULL_1	SUZ12	H.sapiens	SRX027458	SRR067322	n.a.
TBP_ES_NULL_1	TBP	M.musculus	SRX022048	SRR057607	n.a.
TBP_ES_NULL_2	TBP	M.musculus	SRX022047	SRR057606	n.a.
TDRD3_MCF7_NULL_1	TDRD3	H.sapiens	SRX022741	SRR059117	[44]
TDRD3_MCF7_NULL_2	TDRD3	H.sapiens	SRX022741	SRR059118	[44]
TDRD3_MCF7_NULL_3	TDRD3	H.sapiens	SRX022741	SRR059119	[44]
TDRD3_MCF7_NULL_4	TDRD3	H.sapiens	SRX022741	SRR059120	[44]
TRRAP_HeLa_NULL_1	TRRAP	H.sapiens	SRX023652	SRR060525	[40]
TRRAP_HeLa_NULL_2	TRRAP	H.sapiens	SRX023658	SRR060531	[40]
TRX_S2_NULL_1	TRX	D.melanogaster	SRX027830	SRR067913	[16]
TRX_S2_NULL_2	TRX	D.melanogaster	SRX027829	SRR067912	[16]
UBF_HEK293T_NULL_1	UBF	H.sapiens	SRX035783	SRR087746	n.a.
UBF_K562_NULL_1	UBF	H.sapiens	SRX035786	SRR087754	n.a.
VitDr_GM10855_NULL_1	VitDr	H.sapiens	SRX022391	SRR058461	[37]
VitDr_GM10855_NULL_2	VitDr	H.sapiens	SRX022390	SRR058460	[37]
VitDr_GM10855_VitD_1	VitDr	H.sapiens	SRX022393	SRR058463	[37]
VitDr_GM10855_VitD_2	VitDr	H.sapiens	SRX022392	SRR058462	[37]
VitDr_GM10861_NULL_1	VitDr	H.sapiens	SRX022395	SRR058465	[37]
VitDr_GM10861_NULL_2	VitDr	H.sapiens	SRX022394	SRR058464	[37]
VitDr_GM10861_VitD_1	VitDr	H.sapiens	SRX022397	SRR058467	[37]
VitDr_GM10861_VitD_2	VitDr	H.sapiens	SRX022396	SRR058466	[37]

Table S2: **Summary of the shearing technique, inferred average fragment size and interval of fragment sizes to capture 33% of the fragment size distribution**

**Experiment ID**	**Shearing technique**	**Estimated average fragment size [bp]**	**33% fragment size distribution [bp]**
AR_VCaP_NULL_1	Sonication	117	[92 - 142]
BAP18_HeLa_NULL_1	Sonication	191	[158 - 224]
BAP18_HeLa_NULL_2	Sonication	224	[187 - 261]
bCAT_ColCan_NULL_1	Sonication	95	[70 - 120]
BRG1_HESC_NULL_1	Sonication	122	[77 - 167]
CDX2_CACO2_DIFF_1	Sonication	155	[101 - 208]
CDX2_CACO2_DIFF_2	Sonication	112	[87 - 137]
CDX2_CACO2_PROL_1	Sonication	145	[99 - 191]
CTCF_MCF7_NULL_4	Sonication	120	[68 - 171]
CTCF_MEF_NULL_1	Sonication	95	[49 - 140]
CTCF_MEF_NULL_2	Sonication	112	[84 - 140]
DNAInput_c2c12_MB_1	MNase	149	[1 - 407]
DNAInput_c2c12_MB_10	Sonication	238	[130 - 345]
DNAInput_c2c12_MB_2	MNase	140	[1 - 303]
DNAInput_c2c12_MB_3	MNase	127	[1 - 450]
DNAInput_c2c12_MB_4	MNase	127	[1 - 335]
DNAInput_c2c12_MB_5	MNase	140	[1 - 280]
DNAInput_c2c12_MB_6	MNase	149	[71 - 227]
DNAInput_c2c12_MB_7	Sonication	222	[98 - 346]
DNAInput_c2c12_MB_8	Sonication	98	[1 - 247]
DNAInput_c2c12_MB_9	Sonication	253	[127 - 378]
DNAInput_c2c12_MT_1	MNase	139	[1 - 385]
DNAInput_c2c12_MT_10	Sonication	235	[149 - 321]
DNAInput_c2c12_MT_2	MNase	119	[1 - 356]
DNAInput_c2c12_MT_3	MNase	126	[1 - 338]
DNAInput_c2c12_MT_4	MNase	159	[15 - 303]
DNAInput_c2c12_MT_5	MNase	139	[33 - 245]
DNAInput_c2c12_MT_6	MNase	150	[85 - 214]
DNAInput_c2c12_MT_7	Sonication	220	[60 - 380]
DNAInput_c2c12_MT_8	Sonication	241	[93 - 388]
DNAInput_c2c12_MT_9	Sonication	225	[121 - 328]
DNAInput_HEPG2_NULL_1	Sonication	100	[36 - 164]
DNAInput_hESCH1_NULL_1	Sonication	97	[1 - 303]
DNAInput_hESCH1_NULL_2	Sonication	97	[26 - 168]
DNAInput_hESCH1_NULL_3	Sonication	115	[30 - 200]
DNAInput_hESCH1_NULL_4	Sonication	96	[1 - 198]
DNAInput_hESCH1_NULL_5	Sonication	88	[15 - 161]
DNAInput_hESCH1_NULL_6	Sonication	94	[43 - 144]
DNAInput_hESCH1_NULL_7	Sonication	92	[23 - 161]
DNAInput_hESCH1_NULL_8	Sonication	111	[1 - 303]
DNAInput_MCF7_ER_1	Sonication	113	[39 - 187]
DNAInput_MCF7_NULL_1	Sonication	102	[34 - 169]
DNAInput_MCF7_NULL_2	Sonication	125	[45 - 205]
DNAInput_MCF7_NULL_3	Sonication	134	[44 - 224]
DNAInput_SalGlan_NULL_1	Sonication	110	[88 - 131]
DNAInput_T47D_NULL_1	Sonication	106	[11 - 201]
DNAInput_T47D_NULL_2	Sonication	91	[1 - 209]
DNAInput_WLiver_NULL_1	Sonication	138	[1 - 279]
DNAInput_WLiver_NULL_2	Sonication	101	[1 - 287]
DNAInput_WLiver_NULL_3	Sonication	153	[6 - 299]
DNAInput_WLiver_NULL_4	Sonication	88	[1 - 265]
DNAInput_ZR75_NULL_1	Sonication	107	[22 - 191]
DNAInput_ZR75_NULL_2	Sonication	100	[12 - 188]
E2F1_HeLa_NULL_1	Sonication	94	[48 - 139]
E2F4_GM06990_NULL_1	Sonication	157	[75 - 238]
ELF1_Jurkat_NULL_1_C	Sonication	83	[5 - 161]
ERG_CADO_NULL_1_C	Sonication	129	[1 - 294]
ERG_CADO_NULL_2_C	Sonication	122	[1 - 306]
ERG_CADO_NULL_3_C	Sonication	129	[1 - 329]
ERG_CADO_NULL_4_C	Sonication	79	[1 - 374]
ERG_CADO_NULL_5_C	Sonication	75	[1 - 352]
ERG_VCaP_NULL_1	Sonication	209	[1 - 462]
ERG_VCaP_NULL_2	Sonication	109	[1 - 449]
ERG_VCaP_NULL_3	Sonication	105	[1 - 406]
ERG_VCaP_NULL_4	Sonication	102	[1 - 372]
ERG_VCaP_NULL_5	Sonication	95	[1 - 416]
ERG_VCaP_NULL_6	Sonication	104	[1 - 297]
FAIRE_HESC_NULL_1	Sonication	127	[49 - 205]
FLI1_SKNMC_NULL_1_C	Sonication	87	[1 - 413]
FLI1_SKNMC_NULL_2_C	Sonication	102	[1 - 424]
FLI1_SKNMC_NULL_3_C	Sonication	99	[1 - 466]
FLI1_SKNMC_NULL_4_C	Sonication	81	[1 - 460]
FLI1_SKNMC_NULL_5_C	Sonication	98	[1 - 408]
GATA2_HSC_NULL_1	Sonication	102	[41 - 162]
GATA6_CACO2_DIFF_1	Sonication	97	[22 - 171]
GATAD1_HeLa_NULL_1	Sonication	260	[215 - 304]
GATAD1_HeLa_NULL_2	Sonication	195	[165 - 225]
GR_3134_DEX_3	Sonication	167	[131 - 203]

Continued on next page

Table S2 – *Continued from previous page*

Experiment ID	Shearing technique	Estimated average fragment size [bp]	33% fragment size distribution [bp]
GR_3134_DEX_4	Sonication	149	[113 - 185]
GR_3134_DEX_5	Sonication	76	[1 - 549]
GR_3134_DEX_6	Sonication	76	[1 - 480]
GR_3134_NULL_1	Sonication	79	[1 - 214]
GR_3134_NULL_2	Sonication	78	[1 - 202]
GR_ATT20_DEX_1	Sonication	91	[24 - 158]
GR_ATT20_DEX_2	Sonication	94	[15 - 173]
GR_ATT20_DEX_3	Sonication	139	[65 - 213]
GR_ATT20_DEX_4	Sonication	133	[53 - 212]
GR_ATT20_DEX_5	Sonication	139	[72 - 206]
GR_ATT20_DEX_6	Sonication	144	[80 - 208]
GR_ATT20_NULL_1	Sonication	151	[1 - 462]
GR_ATT20_NULL_2	Sonication	85	[1 - 404]
GR_ATT20_NULL_3	Sonication	94	[1 - 441]
GR_ATT20_NULL_4	Sonication	87	[1 - 372]
GR_ATT20_NULL_5	Sonication	80	[1 - 448]
GR_ATT20_NULL_6	Sonication	86	[1 - 423]
H2AK5Ac_HESC_NULL_1	Sonication	112	[44 - 179]
H2AK5Ac_HESC_NULL_2	Sonication	85	[44 - 125]
H2AK5Ac_IMR90_NULL_1	Sonication	101	[62 - 139]
H2AK5Ac_IMR90_NULL_2	Sonication	92	[64 - 119]
H2AK5Ac_IMR90_NULL_3	Sonication	116	[80 - 152]
H2AZ_TCELL_NULL_1	MNase	121	[96 - 146]
H2AZ_TCELL_NULL_2	MNase	122	[100 - 143]
H2BK120Ac_HESC_NULL_1	Sonication	112	[38 - 186]
H2BK120Ac_IMR90_NULL_1	Sonication	113	[85 - 140]
H2BK120Ac_IMR90_NULL_2	Sonication	115	[88 - 141]
H2BK120Ac_IMR90_NULL_3	Sonication	123	[90 - 156]
H2BK120Ac_IMR90_NULL_4	Sonication	125	[92 - 158]
H2BK12Ac_HESC_NULL_1	Sonication	99	[58 - 140]
H2BK12Ac_HESC_NULL_2	Sonication	100	[51 - 148]
H2BK12Ac_IMR90_NULL_1	Sonication	125	[90 - 160]
H2BK12Ac_IMR90_NULL_2	Sonication	126	[97 - 155]
H2BK12Ac_IMR90_NULL_3	Sonication	103	[77 - 129]
H2BK12Ac_IMR90_NULL_4	Sonication	117	[83 - 151]
H2BK15Ac_HESC_NULL_1	Sonication	109	[56 - 162]
H2BK15Ac_HESC_NULL_2	Sonication	91	[31 - 150]
H2BK15Ac_IMR90_NULL_1	Sonication	84	[40 - 127]
H2BK15Ac_IMR90_NULL_2	Sonication	120	[52 - 187]
H2BK15Ac_IMR90_NULL_3	Sonication	105	[72 - 138]
H2BK15Ac_IMR90_NULL_4	Sonication	123	[77 - 168]
H2BK20Ac_HESC_NULL_1	Sonication	108	[47 - 168]
H2BK20Ac_HESC_NULL_2	Sonication	91	[38 - 144]
H2BK20Ac_IMR90_NULL_1	Sonication	119	[92 - 145]
H2BK20Ac_IMR90_NULL_2	Sonication	104	[78 - 130]
H2BK20Ac_IMR90_NULL_3	Sonication	126	[75 - 176]
H2BK20Ac_IMR90_NULL_4	Sonication	115	[64 - 165]
H2BK5Ac_HESC_NULL_1	Sonication	103	[54 - 152]
H2BK5Ac_HESC_NULL_2	Sonication	95	[46 - 143]
H2BK5me1_TCELL_NULL_1	MNase	75	[22 - 127]
H2BK5me1_TCELL_NULL_2	MNase	109	[60 - 157]
H2BK5me1_TCELL_NULL_3	MNase	75	[8 - 141]
H3_38b9_NULL_1_B	Sonication	91	[1 - 334]
H3_c2c12_NULL_1_B	Sonication	93	[1 - 720]
H3_ES_NULL_1	Sonication	92	[1 - 258]
H3_ES_NULL_1_B	Sonication	92	[1 - 495]
H3_ES_TGFB_1_B	Sonication	82	[1 - 419]
H3_MEF_NULL_1	Sonication	99	[1 - 268]
H3_proB_NULL_1	Sonication	101	[7 - 194]
H3_WLiver_NULL_1	Sonication	134	[20 - 248]
H3K14Ac_IMR90_NULL_1	Sonication	118	[81 - 154]
H3K14Ac_IMR90_NULL_2	Sonication	119	[80 - 158]
H3K14Ac_IMR90_NULL_3	Sonication	109	[74 - 143]
H3K14Ac_IMR90_NULL_4	Sonication	121	[72 - 169]
H3K18Ac_c2c12_MB_1	MNase	129	[59 - 198]
H3K18Ac_c2c12_MB_2	MNase	129	[95 - 162]
H3K18Ac_c2c12_MB_3	MNase	149	[110 - 188]
H3K18Ac_c2c12_MT_1	MNase	128	[2 - 254]
H3K18Ac_c2c12_MT_2	MNase	139	[76 - 201]
H3K18Ac_c2c12_MT_3	MNase	139	[82 - 196]
H3K18Ac_HESC_NULL_1	Sonication	115	[60 - 169]
H3K18Ac_HESC_NULL_2	Sonication	126	[61 - 191]
H3K18Ac_IMR90_NULL_1	Sonication	124	[102 - 146]
H3K18Ac_IMR90_NULL_2	Sonication	117	[94 - 139]
H3K18Ac_IMR90_NULL_3	Sonication	115	[88 - 141]
H3K18Ac_IMR90_NULL_4	Sonication	111	[84 - 137]
H3K23Ac_IMR90_NULL_1	Sonication	114	[78 - 149]
H3K23Ac_IMR90_NULL_2	Sonication	122	[82 - 161]
H3K23Ac_IMR90_NULL_3	Sonication	113	[57 - 168]
H3K23Ac_IMR90_NULL_4	Sonication	123	[65 - 181]

Continued on next page

Table S2 – *Continued from previous page*

Experiment ID	Shearing technique	Estimated average fragment size [bp]	33% fragment size distribution [bp]
H3K23me2_HESC_NULL_1	Sonication	115	[41 - 189]
H3K23me2_HESC_NULL_2	Sonication	112	[40 - 183]
H3K27Ac_ES_NULL_1	Sonication	141	[115 - 166]
H3K27Ac_ES_NULL_2	Sonication	144	[114 - 173]
H3K27Ac_HESC_NULL_1	Sonication	91	[53 - 128]
H3K27Ac_HESC_NULL_2	Sonication	110	[81 - 139]
H3K27Ac_HESC_NULL_3	Sonication	111	[90 - 132]
H3K27Ac_HESC_NULL_4	Sonication	156	[128 - 183]
H3K27Ac_IMR90_NULL_1	Sonication	130	[114 - 146]
H3K27Ac_IMR90_NULL_2	Sonication	125	[107 - 142]
H3K27Ac_IMR90_NULL_3	Sonication	126	[108 - 144]
H3K27Ac_IMR90_NULL_4	Sonication	109	[85 - 133]
H3K27Ac_IMR90_NULL_5	Sonication	108	[86 - 129]
H3K27Ac_NEC_NULL_1	Sonication	156	[105 - 207]
H3K27Ac_proB_NULL_1	Sonication	136	[110 - 161]
H3K27Ac_WLiver_NULL_1	Sonication	126	[95 - 156]
H3K27me1_TCELL_NULL_2	MNase	127	[1 - 684]
H3K27me1_TCELL_NULL_3	MNase	129	[1 - 529]
H3K27me1_TCELL_NULL_4	MNase	129	[1 - 545]
H3K27me2_TCELL_NULL_1	MNase	152	[1 - 641]
H3K27me2_TCELL_NULL_2	MNase	151	[1 - 573]
H3K27me2_TCELL_NULL_3	MNase	152	[1 - 587]
H3K27me2_TCELL_NULL_4	MNase	150	[1 - 598]
H3K27me3_BAM_NULL_1	Sonication	148	[54 - 242]
H3K27me3_BAM_NULL_2	Sonication	145	[62 - 227]
H3K27me3_c2c12_MB_1	MNase	150	[79 - 220]
H3K27me3_c2c12_MB_2	MNase	140	[86 - 194]
H3K27me3_c2c12_MB_3	MNase	148	[97 - 198]
H3K27me3_c2c12_MT_1	MNase	150	[89 - 211]
H3K27me3_c2c12_MT_2	MNase	149	[81 - 217]
H3K27me3_c2c12_MT_3	MNase	149	[91 - 206]
H3K27me3_CD4T_NULL_1_C	MNase	127	[91 - 163]
H3K27me3_CD4T_NULL_2_C	MNase	110	[1 - 329]
H3K27me3_CD4T_NULL_3_C	MNase	112	[1 - 355]
H3K27me3_CD4T_S4KO_1_C	MNase	110	[1 - 403]
H3K27me3_CD4T_S4KO_2_C	MNase	111	[1 - 410]
H3K27me3_CD4T_S6KO_1_C	MNase	128	[100 - 155]
H3K27me3_ES_NULL_1_C	MNase	151	[1 - 348]
H3K27me3_ES_NULL_2_C	MNase	151	[1 - 364]
H3K27me3_HeLa_NULL_1	Sonication	243	[226 - 259]
H3K27me3_HESC_NULL_1	Sonication	91	[51 - 130]
H3K27me3_HESC_NULL_10	Sonication	355	[279 - 430]
H3K27me3_HESC_NULL_11	Sonication	145	[87 - 202]
H3K27me3_HESC_NULL_12	Sonication	91	[18 - 163]
H3K27me3_HESC_NULL_13	Sonication	132	[56 - 207]
H3K27me3_HESC_NULL_15	Sonication	90	[1 - 218]
H3K27me3_HESC_NULL_16	Sonication	149	[1 - 347]
H3K27me3_HESC_NULL_17	Sonication	143	[41 - 244]
H3K27me3_HESC_NULL_2	Sonication	115	[104 - 126]
H3K27me3_HESC_NULL_3	Sonication	115	[105 - 125]
H3K27me3_HESC_NULL_4	Sonication	113	[98 - 128]
H3K27me3_HESC_NULL_5	Sonication	112	[99 - 125]
H3K27me3_HESC_NULL_6	Sonication	122	[81 - 163]
H3K27me3_HESC_NULL_7	Sonication	143	[67 - 219]
H3K27me3_HESC_NULL_8	Sonication	79	[1 - 161]
H3K27me3_HESC_NULL_9	Sonication	157	[110 - 203]
H3K27me3_HFGM_NULL_1	Sonication	147	[1 - 391]
H3K27me3_hiPS_NULL_1	Sonication	192	[146 - 238]
H3K27me3_hiPS_NULL_2	Sonication	151	[104 - 198]
H3K27me3_hiPS_NULL_3	Sonication	120	[23 - 217]
H3K27me3_hiPS_NULL_4	Sonication	136	[77 - 194]
H3K27me3_hiPS_NULL_5	Sonication	117	[19 - 215]
H3K27me3_hiPS_NULL_6	Sonication	154	[110 - 198]
H3K27me3_hiPS_PDB2lox_1	Sonication	145	[94 - 196]
H3K27me3_hiPS_PDB2lox_2	Sonication	155	[111 - 199]
H3K27me3_IMR90_NULL_1	Sonication	143	[83 - 203]
H3K27me3_IMR90_NULL_2	Sonication	144	[86 - 202]
H3K27me3_IMR90_NULL_3	Sonication	139	[37 - 240]
H3K27me3_IMR90_NULL_4	Sonication	131	[23 - 238]
H3K27me3_IMR90_NULL_5	Sonication	134	[69 - 198]
H3K27me3_IMR90_NULL_6	Sonication	132	[64 - 199]
H3K27me3_Jurkat_NULL_1	Sonication	111	[43 - 179]
H3K27me3_MDER10T_NULL_1	Sonication	150	[48 - 252]
H3K27me3_MEF_0Div_1	Sonication	181	[109 - 252]
H3K27me3_MEF_1Div_1	Sonication	80	[1 - 205]
H3K27me3_MEF_2Div_1	Sonication	91	[1 - 211]
H3K27me3_MEF_3Div_1	Sonication	165	[52 - 278]
H3K27me3_MEF_NULL_1	Sonication	143	[90 - 196]
H3K27me3_MEF_NULL_2	Sonication	488	[360 - 616]
H3K27me3_MEF_NULL_3	Sonication	196	[126 - 265]

Continued on next page

Table S2 – *Continued from previous page*

**Experiment ID**	**Shearing technique**	**Estimated average fragment size [bp]**	**33% fragment size distribution [bp]**
H3K27me3_NEC_NULL_1	Sonication	148	[94 - 201]
H3K27me3_S2_NULL_1	Sonication	126	[106 - 145]
H3K27me3_TCELL_NULL_1	MNase	130	[1 - 826]
H3K27me3_TCELL_NULL_2	MNase	129	[1 - 817]
H3K27me3_TCELL_NULL_3	MNase	130	[1 - 726]
H3K27me3_TCELL_NULL_4	MNase	130	[1 - 726]
H3K27me3_TSC_NULL_1_C	MNase	149	[1 - 595]
H3K27me3_TSC_NULL_2_C	MNase	150	[1 - 567]
H3K27me3_XEN_NULL_1_C	MNase	150	[1 - 693]
H3K27me3_XEN_NULL_2_C	MNase	151	[1 - 678]
H3K36me1_TCELL_NULL_1	MNase	151	[1 - 597]
H3K36me1_TCELL_NULL_2	MNase	151	[1 - 587]
H3K36me1_TCELL_NULL_3	MNase	150	[1 - 602]
H3K36me3_BAM_NULL_1	Sonication	79	[1 - 233]
H3K36me3_c2c12_MB_1	MNase	139	[76 - 202]
H3K36me3_c2c12_MB_2	MNase	129	[75 - 182]
H3K36me3_c2c12_MB_3	MNase	141	[97 - 185]
H3K36me3_c2c12_MT_1	MNase	138	[81 - 194]
H3K36me3_c2c12_MT_2	MNase	130	[76 - 184]
H3K36me3_c2c12_MT_3	MNase	142	[88 - 195]
H3K36me3_CD4T_NULL_1_C	MNase	122	[95 - 148]
H3K36me3_CD4T_NULL_2_C	MNase	119	[73 - 164]
H3K36me3_CD4T_S4KO_1_C	MNase	122	[61 - 183]
H3K36me3_CD4T_S6KO_1_C	MNase	122	[105 - 139]
H3K36me3_HeLa_NULL_1	Sonication	219	[166 - 271]
H3K36me3_HESC_NULL_1	Sonication	114	[55 - 173]
H3K36me3_HESC_NULL_2	Sonication	91	[36 - 146]
H3K36me3_HESC_NULL_3	Sonication	100	[56 - 144]
H3K36me3_HESC_NULL_4	Sonication	99	[52 - 145]
H3K36me3_HESC_NULL_5	Sonication	101	[41 - 161]
H3K36me3_HESC_NULL_6	Sonication	101	[73 - 128]
H3K36me3_HESC_NULL_7	Sonication	100	[71 - 128]
H3K36me3_HESC_NULL_8	Sonication	81	[21 - 141]
H3K36me3_IMR90_NULL_1	Sonication	109	[69 - 148]
H3K36me3_IMR90_NULL_2	Sonication	135	[44 - 226]
H3K36me3_IMR90_NULL_3	Sonication	123	[65 - 180]
H3K36me3_IMR90_NULL_4	Sonication	135	[79 - 190]
H3K36me3_IMR90_NULL_5	Sonication	122	[33 - 211]
H3K36me3_MEF_1Div_1	Sonication	142	[86 - 197]
H3K36me3_MEF_3Div_1	Sonication	141	[93 - 189]
H3K36me3_MEF_NULL_1	Sonication	319	[247 - 391]
H3K36me3_S2_NULL_1	Sonication	118	[105 - 131]
H3K36me3_TCELL_NULL_1	MNase	121	[37 - 204]
H3K36me3_TCELL_NULL_2	MNase	117	[30 - 204]
H3K36me3_TCELL_NULL_3	MNase	120	[32 - 208]
H3K36me3_TCELL_NULL_4	MNase	120	[1 - 406]
H3K4Ac_HESC_NULL_1	Sonication	109	[42 - 176]
H3K4Ac_IMR90_NULL_1	Sonication	120	[93 - 146]
H3K4Ac_IMR90_NULL_2	Sonication	122	[95 - 149]
H3K4Ac_IMR90_NULL_3	Sonication	121	[95 - 147]
H3K4Ac_IMR90_NULL_4	Sonication	117	[90 - 143]
H3K4me1_c2c12_MB_1	MNase	148	[83 - 213]
H3K4me1_c2c12_MB_2	MNase	139	[78 - 199]
H3K4me1_c2c12_MB_3	MNase	128	[85 - 171]
H3K4me1_c2c12_MT_1	MNase	147	[95 - 198]
H3K4me1_c2c12_MT_2	MNase	129	[77 - 180]
H3K4me1_c2c12_MT_3	MNase	128	[63 - 193]
H3K4me1_ES_NULL_1	Sonication	215	[117 - 313]
H3K4me1_ES_NULL_2	Sonication	146	[81 - 210]
H3K4me1_ES_NULL_3	Sonication	125	[62 - 187]
H3K4me1_ES_NULL_4	Sonication	113	[65 - 161]
H3K4me1_HESC_NULL_1	Sonication	151	[112 - 189]
H3K4me1_HESC_NULL_2	Sonication	101	[66 - 135]
H3K4me1_HESC_NULL_3	Sonication	102	[52 - 151]
H3K4me1_HESC_NULL_4	Sonication	100	[53 - 147]
H3K4me1_HESC_NULL_5	Sonication	126	[1 - 315]
H3K4me1_HESC_NULL_6	Sonication	143	[113 - 173]
H3K4me1_IMR90_NULL_1	Sonication	133	[113 - 152]
H3K4me1_IMR90_NULL_2	Sonication	137	[106 - 168]
H3K4me1_IMR90_NULL_3	Sonication	134	[99 - 168]
H3K4me1_IMR90_NULL_4	Sonication	135	[80 - 190]
H3K4me1_IMR90_NULL_5	Sonication	142	[88 - 196]
H3K4me1_IMR90_NULL_6	Sonication	140	[99 - 181]
H3K4me1_IMR90_NULL_7	Sonication	148	[110 - 185]
H3K4me1_IMR90_NULL_8	Sonication	144	[80 - 207]
H3K4me1_MEF_1Div_1	Sonication	159	[113 - 204]
H3K4me1_MEF_3Div_1	Sonication	162	[119 - 204]
H3K4me1_MEF_NULL_1	Sonication	156	[81 - 231]
H3K4me1_MEF_NULL_2	Sonication	159	[127 - 190]
H3K4me1_MEF_NULL_3	Sonication	203	[114 - 292]

Continued on next page

Table S2 – *Continued from previous page*

**Experiment ID**	**Shearing technique**	**Estimated average fragment size [bp]**	**33% fragment size distribution [bp]**
H3K4me1_NEC_NULL_1	Sonication	157	[122 - 191]
H3K4me1_proB_NULL_1	Sonication	155	[90 - 219]
H3K4me1_TCELL_NULL_1	MNase	113	[73 - 153]
H3K4me1_TCELL_NULL_2	MNase	112	[71 - 153]
H3K4me1_TCELL_NULL_3	MNase	112	[73 - 151]
H3K4me1_TCELL_NULL_4	MNase	111	[73 - 149]
H3K4me1_VCaP_NULL_1	Sonication	122	[25 - 219]
H3K4me1_VCaP_NULL_2	Sonication	127	[32 - 221]
H3K4me1_VCaP_NULL_3	Sonication	126	[37 - 215]
H3K4me1_WLiver_NULL_1	Sonication	112	[79 - 144]
H3K4me2_c2c12_MB_1	MNase	128	[106 - 150]
H3K4me2_c2c12_MB_2	MNase	128	[106 - 149]
H3K4me2_c2c12_MT_1	MNase	140	[119 - 160]
H3K4me2_c2c12_MT_2	MNase	139	[118 - 159]
H3K4me2_HeLa_NULL_1	Sonication	170	[114 - 225]
H3K4me2_HESC_NULL_1	Sonication	91	[65 - 117]
H3K4me2_HESC_NULL_2	Sonication	117	[104 - 130]
H3K4me2_HESC_NULL_3	Sonication	99	[78 - 119]
H3K4me2_HESC_NULL_4	Sonication	117	[100 - 134]
H3K4me2_IMR90_NULL_1	Sonication	124	[109 - 139]
H3K4me2_IMR90_NULL_2	Sonication	124	[108 - 139]
H3K4me2_IMR90_NULL_3	Sonication	117	[96 - 138]
H3K4me2_IMR90_NULL_4	Sonication	121	[97 - 145]
H3K4me2_MEF_0Div_1	Sonication	123	[77 - 168]
H3K4me2_MEF_1Div_1	Sonication	198	[146 - 249]
H3K4me2_MEF_2Div_1	Sonication	80	[24 - 136]
H3K4me2_MEF_3Div_1	Sonication	122	[68 - 176]
H3K4me2_MEF_NULL_1	Sonication	134	[99 - 168]
H3K4me2_TCELL_NULL_1	MNase	130	[108 - 151]
H3K4me2_TCELL_NULL_2	MNase	130	[1 - 340]
H3K4me2_TCELL_NULL_3	MNase	94	[1 - 351]
H3K4me3_BAM_NULL_1	Sonication	78	[15 - 140]
H3K4me3_c2c12_MB_1	MNase	146	[99 - 192]
H3K4me3_c2c12_MB_2	MNase	141	[104 - 178]
H3K4me3_c2c12_MB_3	MNase	139	[104 - 173]
H3K4me3_c2c12_MB_4	MNase	151	[136 - 166]
H3K4me3_c2c12_MT_1	MNase	140	[84 - 196]
H3K4me3_c2c12_MT_2	MNase	140	[88 - 192]
H3K4me3_c2c12_MT_3	MNase	138	[86 - 190]
H3K4me3_c2c12_MT_4	MNase	160	[143 - 176]
H3K4me3_CD4T_NULL_1_C	MNase	128	[106 - 150]
H3K4me3_CD4T_NULL_2_C	MNase	129	[97 - 160]
H3K4me3_CD4T_NULL_3_C	MNase	91	[60 - 121]
H3K4me3_CD4T_S3KO_1_C	MNase	148	[111 - 184]
H3K4me3_CD4T_S4KO_1_C	MNase	110	[56 - 164]
H3K4me3_CD4T_S6KO_1_C	MNase	128	[104 - 152]
H3K4me3_ES_NULL_1_C	MNase	169	[141 - 196]
H3K4me3_ES_NULL_2_C	MNase	161	[127 - 194]
H3K4me3_HeLa_NULL_6	Sonication	212	[193 - 231]
H3K4me3_HESC_NULL_1	Sonication	123	[105 - 140]
H3K4me3_HESC_NULL_10	Sonication	149	[111 - 187]
H3K4me3_HESC_NULL_13	Sonication	140	[103 - 177]
H3K4me3_HESC_NULL_14	Sonication	111	[71 - 150]
H3K4me3_HESC_NULL_15	Sonication	115	[82 - 147]
H3K4me3_HESC_NULL_2	Sonication	142	[124 - 160]
H3K4me3_HESC_NULL_8	Sonication	153	[129 - 177]
H3K4me3_HESC_NULL_9	Sonication	124	[98 - 150]
H3K4me3_HFGM_NULL_1	Sonication	137	[67 - 206]
H3K4me3_hiPS_NULL_1	Sonication	122	[97 - 147]
H3K4me3_hiPS_NULL_2	Sonication	142	[119 - 164]
H3K4me3_hiPS_NULL_3	Sonication	126	[87 - 165]
H3K4me3_hiPS_NULL_4	Sonication	126	[101 - 150]
H3K4me3_hiPS_NULL_5	Sonication	141	[104 - 178]
H3K4me3_hiPS_NULL_6	Sonication	124	[96 - 151]
H3K4me3_hiPS_PDB2lox_1	Sonication	134	[108 - 159]
H3K4me3_IMR90_NULL_1	Sonication	130	[117 - 142]
H3K4me3_IMR90_NULL_2	Sonication	134	[121 - 146]
H3K4me3_Jurkat_NULL_1	Sonication	97	[25 - 169]
H3K4me3_MCF7_NULL_1	NA	159	[42 - 276]
H3K4me3_MCF7_NULL_2	NA	139	[30 - 248]
H3K4me3_MCF7_NULL_3	NA	87	[29 - 145]
H3K4me3_MCF7_NULL_4	NA	81	[24 - 138]
H3K4me3_MCF7_NULL_5	NA	112	[43 - 181]
H3K4me3_MCF7_NULL_6	NA	98	[36 - 159]
H3K4me3_MEF_1Div_1	Sonication	151	[123 - 179]
H3K4me3_MEF_3Div_1	Sonication	155	[131 - 179]
H3K4me3_NEC_NULL_1	Sonication	132	[110 - 153]
H3K4me3_S2_NULL_3	Sonication	103	[85 - 121]
H3K4me3_TCELL_NULL_2	MNase	151	[133 - 169]
H3K4me3_TCELL_NULL_3	MNase	148	[130 - 166]

Continued on next page

Table S2 – *Continued from previous page*

Experiment ID	Shearing technique	Estimated average fragment size [bp]	33% fragment size distribution [bp]
H3K4me3_TCELL_NULL_4	MNase	148	[130 - 166]
H3K4me3_TCELL_NULL_5	MNase	151	[131 - 170]
H3K4me3_TCELL_NULL_6	MNase	151	[131 - 171]
H3K4me3_TCELL_NULL_7	MNase	131	[108 - 154]
H3K4me3_TSC_NULL_1_C	MNase	161	[143 - 179]
H3K4me3_TSC_NULL_2_C	MNase	160	[142 - 177]
H3K4me3_XEN_NULL_1_C	MNase	161	[144 - 177]
H3K4me3_XEN_NULL_2_C	MNase	159	[143 - 175]
H3K56Ac_HSEC_NULL_1	Sonication	96	[3 - 188]
H3K56Ac_IMR90_NULL_1	Sonication	123	[79 - 167]
H3K56Ac_IMR90_NULL_2	Sonication	139	[91 - 186]
H3K56Ac_IMR90_NULL_3	Sonication	126	[77 - 174]
H3K79me1_HESC_NULL_1	Sonication	102	[17 - 187]
H3K79me1_HESC_NULL_2	Sonication	80	[1 - 193]
H3K79me1_HESC_NULL_3	Sonication	92	[15 - 168]
H3K79me1_IMR90_NULL_1	Sonication	104	[52 - 155]
H3K79me1_IMR90_NULL_2	Sonication	84	[37 - 131]
H3K79me1_IMR90_NULL_3	Sonication	97	[42 - 151]
H3K79me1_IMR90_NULL_4	Sonication	97	[46 - 148]
H3K79me1_IMR90_NULL_5	Sonication	98	[52 - 143]
H3K79me1_IMR90_NULL_6	Sonication	110	[59 - 161]
H3K79me1_IMR90_NULL_7	Sonication	105	[42 - 168]
H3K79me1_TCELL_NULL_1	MNase	78	[1 - 649]
H3K79me1_TCELL_NULL_2	MNase	82	[1 - 650]
H3K79me1_TCELL_NULL_3	MNase	78	[1 - 649]
H3K79me2_HESC_NULL_1	Sonication	91	[40 - 141]
H3K79me2_HESC_NULL_2	Sonication	81	[24 - 138]
H3K79me2_HESC_NULL_3	Sonication	97	[62 - 131]
H3K79me2_IMR90_NULL_1	Sonication	79	[56 - 101]
H3K79me2_IMR90_NULL_2	Sonication	109	[79 - 139]
H3K79me2_IMR90_NULL_3	Sonication	115	[79 - 151]
H3K79me2_IMR90_NULL_4	Sonication	109	[71 - 147]
H3K79me2_TCELL_NULL_1	MNase	148	[1 - 690]
H3K79me3_HeLa_NULL_1	Sonication	214	[172 - 256]
H3K79me3_MEF_NULL_1	Sonication	75	[55 - 95]
H3K79me3_MEF_NULL_2	Sonication	95	[34 - 155]
H3K79me3_TCELL_NULL_1	MNase	90	[1 - 242]
H3K79me3_TCELL_NULL_2	MNase	90	[1 - 195]
H3K79me3_TCELL_NULL_3	MNase	92	[1 - 202]
H3K9Ac_c2c12_MB_1	MNase	139	[93 - 185]
H3K9Ac_c2c12_MB_2	MNase	128	[71 - 185]
H3K9Ac_c2c12_MB_3	MNase	149	[127 - 170]
H3K9Ac_c2c12_MT_1	MNase	128	[1 - 352]
H3K9Ac_c2c12_MT_2	MNase	128	[1 - 288]
H3K9Ac_c2c12_MT_3	MNase	139	[82 - 196]
H3K9Ac_HeLa_NULL_1	Sonication	213	[197 - 229]
H3K9Ac_HESC_NULL_1	Sonication	104	[75 - 132]
H3K9Ac_HESC_NULL_2	Sonication	127	[95 - 158]
H3K9Ac_IMR90_NULL_1	Sonication	125	[107 - 143]
H3K9Ac_IMR90_NULL_2	Sonication	125	[103 - 147]
H3K9Ac_IMR90_NULL_3	Sonication	136	[112 - 159]
H3K9me1_TCELL_NULL_1	MNase	129	[20 - 238]
H3K9me1_TCELL_NULL_2	MNase	128	[18 - 237]
H3K9me1_TCELL_NULL_3	MNase	91	[1 - 327]
H3K9me1_TCELL_NULL_4	MNase	129	[106 - 151]
H3K9me2_DMLarvae_EHMTKO_1	Sonication	187	[172 - 202]
H3K9me2_DMLarvae_NULL_1	Sonication	186	[175 - 196]
H3K9me2_TCELL_NULL_1	MNase	91	[1 - 760]
H3K9me2_TCELL_NULL_2	MNase	91	[1 - 719]
H3K9me2_TCELL_NULL_3	MNase	92	[1 - 731]
H3K9me3_HeLa_NULL_1	Sonication	204	[45 - 363]
H3K9me3_HESC_NULL_1	Sonication	83	[33 - 133]
H3K9me3_HESC_NULL_2	Sonication	92	[29 - 155]
H3K9me3_HESC_NULL_3	Sonication	103	[46 - 160]
H3K9me3_HESC_NULL_4	Sonication	101	[50 - 152]
H3K9me3_HESC_NULL_5	Sonication	104	[54 - 154]
H3K9me3_HESC_NULL_6	Sonication	103	[58 - 148]
H3K9me3_HESC_NULL_7	Sonication	91	[44 - 137]
H3K9me3_HESC_NULL_8	Sonication	95	[43 - 146]
H3K9me3_IMR90_NULL_1	Sonication	75	[52 - 98]
H3K9me3_IMR90_NULL_2	Sonication	143	[69 - 216]
H3K9me3_IMR90_NULL_3	Sonication	147	[70 - 224]
H3K9me3_IMR90_NULL_4	Sonication	140	[39 - 240]
H3K9me3_IMR90_NULL_5	Sonication	146	[44 - 248]
H3K9me3_IMR90_NULL_6	Sonication	137	[58 - 215]
H3K9me3_IMR90_NULL_7	Sonication	150	[73 - 226]
H3K9me3_MDER10T_NULL_1	Sonication	137	[33 - 241]
H3K9me3_TCELL_NULL_1	MNase	78	[1 - 515]
H3K9me3_TCELL_NULL_2	MNase	77	[1 - 510]
H3K9me3_TCELL_NULL_3	MNase	78	[1 - 482]

Continued on next page

Table S2 – *Continued from previous page*

**Experiment ID**	**Shearing technique**	**Estimated average fragment size [bp]**	**33% fragment size distribution [bp]**
H3K9me3_TCELL_NULL_4	MNase	77	[1 - 536]
H3R2me1_TCELL_NULL_1	MNase	150	[1 - 586]
H3R2me1_TCELL_NULL_2	MNase	149	[1 - 602]
H3R2me1_TCELL_NULL_3	MNase	128	[1 - 585]
H3R2me2_TCELL_NULL_1	MNase	75	[1 - 643]
H3R2me2_TCELL_NULL_2	MNase	151	[1 - 374]
H4K12Ac_c2c12_MB_1	MNase	158	[31 - 284]
H4K12Ac_c2c12_MB_2	MNase	150	[2 - 297]
H4K12Ac_c2c12_MB_3	MNase	158	[145 - 170]
H4K12Ac_c2c12_MB_4	MNase	149	[127 - 170]
H4K12Ac_c2c12_MT_1	MNase	158	[2 - 313]
H4K12Ac_c2c12_MT_2	MNase	139	[102 - 175]
H4K12Ac_c2c12_MT_3	MNase	139	[101 - 176]
H4K12Ac_c2c12_MT_4	MNase	147	[91 - 203]
H4K20me1_HeLa_NULL_1	Sonication	81	[1 - 161]
H4K20me1_HESC_NULL_1	Sonication	90	[41 - 138]
H4K20me1_IMR90_NULL_1	Sonication	85	[31 - 138]
H4K20me1_IMR90_NULL_2	Sonication	89	[41 - 137]
H4K20me1_IMR90_NULL_3	Sonication	109	[58 - 159]
H4K20me1_IMR90_NULL_4	Sonication	101	[36 - 166]
H4K20me1_TCELL_NULL_1	MNase	77	[42 - 112]
H4K20me1_TCELL_NULL_2	MNase	75	[40 - 109]
H4K20me1_TCELL_NULL_3	MNase	75	[35 - 114]
H4K20me3_TCELL_NULL_1	MNase	75	[34 - 116]
H4K20me3_TCELL_NULL_2	MNase	75	[33 - 117]
H4K20me3_TCELL_NULL_3	MNase	75	[32 - 118]
H4K5Ac_HESC_NULL_1	Sonication	111	[84 - 137]
H4K5Ac_HESC_NULL_2	Sonication	111	[64 - 157]
H4K5Ac_IMR90_NULL_1	Sonication	126	[106 - 145]
H4K5Ac_IMR90_NULL_2	Sonication	125	[103 - 146]
H4K5Ac_IMR90_NULL_3	Sonication	115	[89 - 140]
H4K5Ac_IMR90_NULL_4	Sonication	126	[98 - 154]
H4K8Ac_IMR90_NULL_1	Sonication	119	[92 - 146]
H4K8Ac_IMR90_NULL_2	Sonication	93	[73 - 113]
H4K8Ac_IMR90_NULL_3	Sonication	123	[92 - 153]
H4K8Ac_IMR90_NULL_4	Sonication	119	[98 - 139]
H4K8Ac_IMR90_NULL_5	Sonication	116	[90 - 141]
H4K91Ac_HESC_NULL_1	Sonication	90	[46 - 133]
H4K91Ac_IMR90_NULL_1	Sonication	111	[86 - 135]
H4K91Ac_IMR90_NULL_2	Sonication	124	[93 - 155]
H4K91Ac_IMR90_NULL_3	Sonication	116	[84 - 147]
H4R3me2_TCELL_NULL_1	MNase	148	[1 - 330]
H4R3me2_TCELL_NULL_2	MNase	151	[1 - 497]
H4R3me2_TCELL_NULL_3	MNase	162	[1 - 526]
H4R3me2_TCELL_NULL_4	MNase	81	[1 - 494]
hERa_MCF7_NULL_2	Sonication	100	[77 - 122]
hERa_MCF7_NULL_3	Sonication	120	[83 - 157]
hERa_MCF7_NULL_5	Sonication	113	[76 - 150]
hERa_MCF7_NULL_7	Sonication	118	[60 - 175]
hERa_T47D_NULL_1	Sonication	109	[47 - 171]
hERa_T47D_NULL_2	Sonication	119	[56 - 181]
hERa_T47D_NULL_3	Sonication	132	[66 - 197]
hERa_T47D_NULL_4	Sonication	122	[60 - 183]
hERa_T47D_NULL_5	Sonication	118	[44 - 191]
hERa_ZR75_NULL_1	Sonication	106	[69 - 143]
hERa_ZR75_NULL_2	Sonication	104	[68 - 139]
hERa_ZR75_NULL_3	Sonication	133	[102 - 164]
hERa_ZR75_NULL_5	Sonication	131	[98 - 164]
HNF4A_CACO2_DIFF_1	Sonication	114	[96 - 131]
HNF4A_CACO2_PROL_1	Sonication	80	[66 - 93]
HNF4A_HEPG2_NULL_1	Sonication	103	[83 - 123]
HOXC9_Ainv15_NULL_1_C	Sonication	80	[1 - 159]
HOXC9_Ainv15_NULL_2_C	Sonication	97	[23 - 171]
IgG_CADO_NULL_1_C	Sonication	135	[47 - 223]
IgG_CADO_NULL_2_C	Sonication	139	[50 - 227]
IgG_GM12878_NULL_1_C	Sonication	130	[7 - 253]
IgG_GM12878_NULL_2_C	Sonication	134	[12 - 256]
IgG_GM12878_NULL_3_C	Sonication	110	[1 - 320]
IgG_GM12891_NULL_1_C	Sonication	126	[13 - 238]
IgG_GM18526_NULL_1_C	Sonication	134	[1 - 306]
IgG_GM18526_NULL_2_C	Sonication	100	[1 - 299]
IgG_GM18526_NULL_3_C	Sonication	82	[1 - 171]
IgG_GM18526_NULL_4_C	Sonication	82	[1 - 188]
IgG_GM18526_NULL_5_C	Sonication	89	[1 - 248]
IgG_GM18526_NULL_6_C	Sonication	91	[1 - 204]
IgG_GM18951_NULL_1_C	Sonication	127	[8 - 245]
IgG_GM18951_NULL_2_C	Sonication	96	[1 - 286]
IgG_GM18951_NULL_3_C	Sonication	93	[1 - 231]
IgG_GM18951_NULL_4_C	Sonication	89	[1 - 230]
IgG_HESC_NULL_1_C	Sonication	131	[12 - 250]

Continued on next page

Table S2 – *Continued from previous page*

**Experiment ID**	**Shearing technique**	**Estimated average fragment size [bp]**	**33% fragment size distribution [bp]**
IgG_HL60_NULL_1_C	Sonication	98	[13 - 182]
IgG_HSC_NULL_1	Sonication	88	[1 - 758]
IgG_HSC_NULL_2	Sonication	97	[1 - 827]
IgG_Jurkat_NULL_1	Sonication	87	[1 - 446]
IgG_MCF7_NULL_1	Sonication	171	[149 - 192]
IgG_SKNMC_NULL_1	Sonication	88	[1 - 582]
IgG_SKNMC_NULL_2	Sonication	204	[1 - 548]
IgG_SKNMC_NULL_3	Sonication	85	[1 - 363]
IgG_VCaP_NULL_1	Sonication	150	[51 - 249]
IgG_VCaP_NULL_2	Sonication	135	[36 - 234]
IgG_VCaP_NULL_3	Sonication	115	[29 - 201]
IgG_VCaP_NULL_4	Sonication	137	[42 - 232]
IgG_VCaP_NULL_5	Sonication	121	[1 - 282]
IgG_VCaP_NULL_6	Sonication	92	[1 - 352]
LDB1_HSC_NULL_1	Sonication	113	[79 - 146]
LRWD1_HeLa_NULL_1	Sonication	193	[114 - 272]
MCRS2_SalGlan_NULL_1	Sonication	109	[95 - 123]
MED1_ES_NULL_1	Sonication	93	[52 - 133]
MED1_ES_NULL_2	Sonication	150	[116 - 183]
MED1_MEF_NULL_1	Sonication	100	[44 - 156]
MED12_ES_NULL_1	Sonication	93	[54 - 131]
MED12_MEF_NULL_1	Sonication	86	[7 - 165]
MED12_MEF_NULL_2	Sonication	91	[64 - 117]
MSL3TAP_Dmel_NULL_1	Sonication	94	[55 - 132]
NF1_MEF_NFICKO_1	Sonication	165	[129 - 200]
NF1_MEF_WT_1	Sonication	144	[105 - 182]
NFATC1_SELEX_NULL_1_C	Sonication	110	[80 - 140]
NFATC1_SELEX_NULL_2_C	Sonication	101	[49 - 152]
NIPBL_ES_NULL_1	Sonication	105	[61 - 148]
NIPBL_ES_NULL_2	Sonication	88	[12 - 163]
NSL1_SalGlan_NULL_1	Sonication	106	[92 - 119]
NULL_CD4T_NULL_1_C	MNase	103	[60 - 146]
NULL_CD4T_S4KO_1_C	MNase	86	[36 - 136]
NULL_CD4T_S6KO_1_C	MNase	100	[65 - 134]
NULL_HeLa_NULL_1	Sonication	179	[127 - 230]
OCT4_HESC_NULL_1_C	Sonication	120	[50 - 190]
OCT4_HESC_NULL_2_C	Sonication	125	[49 - 200]
OCT4_HESC_NULL_3_C	Sonication	123	[53 - 192]
P130_QuiFib_NULL_1	Sonication	233	[164 - 301]
P130_QuiFib_NULL_2	Sonication	89	[50 - 127]
P130_SenFib_NULL_1	Sonication	107	[73 - 141]
P130_SenFib_NULL_2	Sonication	110	[75 - 144]
P300_CD1_BRAIN_1	Sonication	80	[1 - 380]
P300_CD1_HEART_1	Sonication	89	[1 - 253]
P300_ES_NULL_1	Sonication	125	[88 - 162]
P300_ES_NULL_2	Sonication	133	[98 - 167]
P300_HESC_NULL_1	Sonication	160	[122 - 198]
P300_NEC_NULL_1	Sonication	111	[60 - 162]
PC_S2_NULL_1	Sonication	149	[134 - 163]
PC_S2_NULL_2	Sonication	82	[61 - 102]
PH_S2_NULL_1	Sonication	111	[96 - 126]
PH_S2_NULL_2	Sonication	113	[89 - 136]
PHF8_HeLa_NULL_1	Sonication	109	[66 - 152]
PHF8_HeLa_NULL_2	Sonication	194	[163 - 225]
PHF8_HeLa_NULL_3	Sonication	257	[215 - 299]
PolII_BAM_NULL_1	Sonication	75	[18 - 131]
PolII_c2c12_MB_1	Sonication	223	[132 - 313]
PolII_c2c12_MB_2	Sonication	222	[125 - 318]
PolII_c2c12_MB_3	Sonication	243	[193 - 293]
PolII_c2c12_MT_1	Sonication	228	[106 - 350]
PolII_c2c12_MT_2	Sonication	230	[121 - 338]
PolII_c2c12_MT_3	Sonication	243	[200 - 285]
PolII_GM12878_NULL_1_C	Sonication	79	[46 - 112]
PolII_GM12878_NULL_2_C	Sonication	85	[57 - 113]
PolII_GM12878_NULL_3_C	Sonication	75	[30 - 119]
PolII_GM12878_NULL_4_C	Sonication	83	[49 - 117]
PolII_GM12878_NULL_5_C	Sonication	101	[67 - 135]
PolII_GM12878_NULL_6_C	Sonication	89	[54 - 123]
PolII_GM12878_NULL_7_C	Sonication	93	[60 - 125]
PolII_GM12878_NULL_8_C	Sonication	82	[50 - 114]
PolII_GM12878_NULL_9_C	Sonication	80	[55 - 105]
PolII_GM12891_NULL_1_C	Sonication	144	[100 - 187]
PolII_GM12891_NULL_2_C	Sonication	147	[100 - 193]
PolII_GM12891_NULL_3_C	Sonication	159	[112 - 206]
PolII_GM12891_NULL_4_C	Sonication	136	[98 - 174]
PolII_GM12891_NULL_5_C	Sonication	152	[116 - 188]
PolII_GM12891_NULL_6_C	Sonication	137	[98 - 175]
PolII_GM18526_NULL_1_C	Sonication	78	[58 - 98]
PolII_GM18526_NULL_2_C	Sonication	90	[60 - 119]
PolII_GM18951_NULL_1_C	Sonication	75	[49 - 100]

Continued on next page

Table S2 – *Continued from previous page*

**Experiment ID**	**Shearing technique**	**Estimated average fragment size [bp]**	**33% fragment size distribution [bp]**
PolII_HBG3_S2P_1	Sonication	82	[1 - 179]
PolII_HBG3_S2P_2	Sonication	98	[7 - 189]
PolII_Jurkat_10DRB_1	Sonication	122	[92 - 151]
PolII_Jurkat_DMSO_1	Sonication	112	[83 - 140]
PolII_Jurkat_NULL_1	Sonication	82	[1 - 278]
PolII_Jurkat_NULL_2	Sonication	99	[13 - 184]
PolII_MCF7_NULL_1	NA	86	[1 - 540]
PolII_MCF7_NULL_4	NA	219	[7 - 431]
PolII_MCF7_NULL_6	NA	185	[1 - 517]
PolII_MEF_NULL_2	Sonication	101	[56 - 146]
PolII_MEF_NULL_3	Sonication	95	[56 - 134]
PolII_S2_NULL_1	Sonication	78	[57 - 99]
PolII_TCELL_NULL_2	MNase	76	[30 - 121]
PolII_TCELL_NULL_3	MNase	80	[16 - 143]
PolII_TCELL_NULL_4	MNase	78	[35 - 120]
pRb_GroFib_NULL_1	Sonication	88	[18 - 157]
pRb_GroFib_NULL_2	Sonication	84	[38 - 130]
pRb_QuiFib_NULL_1	Sonication	91	[1 - 185]
pRb_QuiFib_NULL_2	Sonication	252	[120 - 384]
pRb_QuiFib_NULL_3	Sonication	91	[26 - 156]
pRb_QuiFib_NULL_4	Sonication	91	[46 - 135]
pRb_SenFib_NULL_1	Sonication	116	[30 - 202]
pRb_SenFib_NULL_2	Sonication	133	[58 - 207]
pRb_SenFib_NULL_3	Sonication	87	[28 - 146]
pRb_SenFib_NULL_4	Sonication	75	[38 - 112]
PSC_KC_NULL_1	Sonication	122	[101 - 143]
PSC_KC_NULL_2	Sonication	119	[96 - 141]
PSC_KC_NULL_3	Sonication	121	[100 - 142]
PSC_S2_NULL_1	Sonication	107	[91 - 123]
PSC_S2_NULL_2	Sonication	94	[75 - 113]
RAD21_HEPG2_NULL_1	Sonication	101	[78 - 123]
RAR_HBG3_NULL_1_C	Sonication	121	[56 - 186]
RAR_HBG3_NULL_2_C	Sonication	122	[54 - 190]
RAR_HBG3_NULL_3_C	Sonication	121	[55 - 187]
RAR_HBG3_NULL_4_C	Sonication	112	[48 - 175]
RAR_HBG3_NULL_5_C	Sonication	133	[61 - 205]
RAR_HBG3_NULL_6_C	Sonication	112	[59 - 165]
RAR_HBG3_RA_1_C	Sonication	121	[58 - 183]
RAR_HBG3_RA_2_C	Sonication	143	[45 - 240]
RAR_HBG3_RA_3_C	Sonication	122	[44 - 199]
RAR_HBG3_RA_4_C	Sonication	132	[69 - 194]
RAR_HBG3_RA_5_C	Sonication	89	[8 - 169]
RAR_HBG3_RA_6_C	Sonication	133	[34 - 231]
RPA116_HEK293T_NULL_1	Sonication	75	[1 - 167]
SCLTAL1_HSC_NULL_1	Sonication	112	[82 - 142]
SGF29_HeLa_NULL_1	Sonication	208	[154 - 261]
SGF29_HeLa_NULL_2	Sonication	192	[140 - 244]
SMC1_ES_NULL_1	Sonication	91	[66 - 115]
SMC1_ES_NULL_2	Sonication	108	[76 - 139]
SMC1_MEF_NULL_1	Sonication	91	[27 - 154]
SMC3_ES_NULL_1	Sonication	116	[89 - 142]
SMC3_ES_NULL_2	Sonication	95	[71 - 119]
SMC4_HeLa_NULL_1	Sonication	175	[136 - 214]
SOX2_ES_NULL_1	Sonication	124	[86 - 162]
SPDEF_VCaP_NULL_1	Sonication	132	[1 - 336]
SPDEF_VCaP_NULL_2	Sonication	123	[1 - 338]
SPDEF_VCaP_NULL_3	Sonication	111	[1 - 326]
SPDEF_VCaP_NULL_4	Sonication	97	[1 - 317]
SPDEF_VCaP_NULL_5	Sonication	98	[1 - 331]
SPDEF_VCaP_NULL_6	Sonication	106	[1 - 337]
SPDEF_VCaP_NULL_7	Sonication	86	[1 - 334]
SPDEF_VCaP_NULL_8	Sonication	96	[1 - 338]
SPDEF_VCaP_NULL_9	Sonication	80	[1 - 349]
STAT3_CD4T_NULL_1_C	MNase	148	[115 - 180]
STAT4_CD4T_NULL_1_C	MNase	117	[94 - 139]
STAT6_CD4T_NULL_1_C	MNase	93	[73 - 113]
SUZ12_HESC_NULL_1	Sonication	75	[1 - 160]
TBP_ES_NULL_1	Sonication	76	[40 - 111]
TBP_ES_NULL_2	Sonication	98	[62 - 133]
TDRD3_MCF7_NULL_1	Sonication	159	[139 - 179]
TDRD3_MCF7_NULL_2	Sonication	162	[141 - 183]
TDRD3_MCF7_NULL_3	Sonication	130	[114 - 145]
TDRD3_MCF7_NULL_4	Sonication	130	[115 - 145]
TRRAP_HeLa_NULL_1	Sonication	233	[216 - 250]
TRRAP_HeLa_NULL_2	Sonication	193	[134 - 252]
TRX_S2_NULL_1	Sonication	127	[114 - 139]
TRX_S2_NULL_2	Sonication	75	[59 - 91]
UBF_HEK293T_NULL_1	Sonication	108	[33 - 183]
UBF_K562_NULL_1	Sonication	89	[14 - 164]
VitDr_GM10855_NULL_1	Sonication	117	[61 - 172]

Continued on next page

Table S2 – *Continued from previous page*

**Experiment ID**	**Shearing technique**	**Estimated average fragment size [bp]**	33% **fragment size distribution [bp]**
VitDr_GM10855_NULL_2	Sonication	129	[64 - 194]
VitDr_GM10855_VitD_1	Sonication	111	[70 - 152]
VitDr_GM10855_VitD_2	Sonication	107	[69 - 144]
VitDr_GM10861_NULL_1	Sonication	130	[70 - 189]
VitDr_GM10861_NULL_2	Sonication	120	[42 - 198]
VitDr_GM10861_VitD_1	Sonication	125	[82 - 168]
VitDr_GM10861_VitD_2	Sonication	122	[79 - 165]

Table S3: **ChIP-Seq protocols transcribed verbatim from their original reports. Only the description of the ChIP-Seq methods is shown.**

**Protocol**	**Study Reference**
ChIP was performed as described [74], and **sonication was performed to obtain chromatin fragments of approximately 250 bp whereas MNase digestion produced approximately 150 bp fragments**. Antibodies used for ChIP are listed in SI Materials and Methods. Quantitative ChIP for validation was performed using three or more independent biological replicates, except as noted. ChIP enriched DNA was analyzed using real-time PCR (qChIP), and in every case, background was assessed using a negative control antibody (rabbit IgG). For all experiments shown, the IgG control produced an average enrichment $\leq$ 0.05% of the input for a given site.	[4]
$CD4^+$ T cells were purified from human blood using human $CD4^+$ T cell isolation kit II kits (Miltenyi). The cells were digested with MNase to generate mainly mononucleosomes with minor fraction of dinucleosomes for histone modification mapping. For mapping enzyme target sites, the cells were crosslinked with formaldehyde treatment and chromatin **fragmented to 200 to 300 bp by sonication**. Chromatin from 2×10^7 cells was used for each ChIP experiment, which yielded approximately 200 ng of DNA. The ChIP DNA ends were repaired using PNK and Klenow enzyme, followed by treatment with Taq polymerase to generate a protruding 3' A base used for adaptor ligation. Following ligation of a pair of Solexa adaptors to the repaired ends, the ChIP DNA was amplified using the adaptor primers for 17 cycles and the **fragments around 220 bp** (mononucleosome + adaptors) isolated from agarose gel. The purified DNA was used directly for cluster generation and sequencing analysis using the Solexa 1G Genome Analyzer following manufacturer protocols.	[5]
Chromatin immunoprecipitation (ChIP) for SOX2 (R&D Systems, #AF2018; 5μg) and NANOG (R&D Systems, #AF1997, 5μg) was performed as recently described (Hawkins et al., submitted). ChIP for OCT4 (Santa Cruz, #sc8626, 2μg; Santa Cruz, #sc9081, 2μg; R&D Systems, #AF17566, 2μg), p300 (Santa Cruz, #sc585, 5μg), KLF4 (Abcam, #ab21949, 10μg) TAFIIp250/TAF1 (Santa Cruz, #sc735, 5μg) were carried out as previously described with 500μg chromatin and 2-10μg antibody [75, 76]. ChIP libraries for sequencing were prepared following standard protocols from Illumina (San Diego, CA) with the following minor modifications. Following linker ligation, libraries were run on an 8% acrylamide gel **and size selected for 175 - 250bp**. This was repeated following PCR amplification. After each size selection, acrylamide was shredded and incubated with 300μl EB buffer (Qiagen, Valencia, CA) overnight at 4°C or 50°C for 20 mins with shaking. DNA was eluted using Nanosep MF filter tubes (Pall, East Hills, NY). The experimental detail and in depth data analysis of the histone modifications will be described separately Hawkins et al., submitted.	[9]
ChIP profiling of histone modifications and RNA polymerase II using Affymetrix tiling arrays was performed and analyzed as previously described [77]. [...] We prepared libraries for Illumina sequencing of Psc ChIP with the Illumina ChIP-seq DNA Sample Prep Kit (Cat# IP-102-1001) according to Illumina's instructions and sequenced on the Genome Analyzer 2 following the manufacturer's protocols.	[10]
Embryonic heart and midbrain tissues were isolated from approximately 270 CD-1 strain embryos at e11.5 respectively by microdissection in cold PBS. Tissue samples were processed for ChIP and DNA sequencing as described previously [78]. Briefly, tissues were cross-linked in formaldehyde and cells dissociated in a glass douncer. Chromatin isolation, sonication, and immunoprecipitation using an anti-p300 antibody (rabbit polyclonal anti-p300; SC-585, Santa Cruz Biotechnology) were performed as previously described [79, 80]. Approximately 0.1 ng of each ChIP DNA sample was sheared by sonication, end-repaired, ligated to sequencing adapters and amplified by emulsion PCR for 40 cycles [81]. Amplified ChIP DNA was sequenced for 36 cycles on the Illumina Genome Analyzer II as described previously [78].	[11]
Antibodies used in ChIP assays included: 3 μg of anti-β–catenin (BD transduction, 610154), 3 μg of anti-TCF4 (Millipore, 05-511), 2 μl of anti-c-Jun (Millipore, 06-225) and 6 μg rabbit anti mouse IgG (Jackson Immunoresearch, 315-005-003). β–Catenin ChIP DNA for the ChIP-Seq library was prepared using the Chromatin Immunoprecipitation Assay Kit (Millipore, 17-295) according to the instructions. To assess β–catenin, TCF4 and c-Jun binding to ChIP-Seq peak loci, ChIP assays contained 5–10 $\times 10^6$ cells and were conducted as previously reported [82]. Chromatin in formaldehyde fixed cell lysates **was sonicated to an average size of 500–700 bp** using a Misonix Ultrasonic XL-2000 Liquid Processor (5 $\times$ 20 s, output wattage 7, with 45 s rest intervals on ice between pulses). [...] β–Catenin precipitated and purified ChIP DNA (350 ng) was processed using the ChIP-Seq DNA Sample Preparation Kit (Illumina, 1003473) according to instructions provided by the manufacturer. Prior to sequencing, DNAs were re-quantified using a NanoDrop 1000 Spectrophotometer and the quality of DNA was assessed using a Bioanalyzer DNA 1000 (Agilent). Samples were diluted to 10 nM and 54-nt reads were obtained from one lane of sequencing on a Illumina GA II sequencer. The High Throughput Sequencing Facility at the University of Oregon (http://htseq.uoregon.edu) sequenced the library.	[12]

Continued on next page

Table S3 – *Continued from previous page*

Protocol	Study Reference
Chromatin immunoprecipitation (ChIP).<br>4×10^7 cells of each condition were pre-fix for 30 minutes in 1.5mM Disuccinimidyl glutarate (EGS) followed by fixing in 4% paraformaldehyde at room temperature for 10 min. The cross-linking reaction was quenched with 0.125M Glycine followed by several washes with cold PBS. The pellets were washed for 15 minutes at RT with buffer 1 (10mM Tris HCl (pH 8.0), 0.25% Triton X-100, 10 mM EDTA, 0.5 mM EGTA, 1mM PMSF) and buffer 2 (10mM Tris HCl (pH 8.0), 200 mM NaCl, 10 mM EDTA, 0.5 mM EGTA) 1mM PMSF). The pellets were resuspended in 2mL buffer 3 (10mM Tris HCl (pH 8.0), 1 mM EDTA, 0.5 mM EGTA 1mM PMSF) and **fragmented to an average size of 100–200 bp** using the Biodisruptor. The buffer was adjusted for IP (IP buffer - 20mM Tris HCl (pH 8.0), 200 mM NaCl, 1 mM EDTA, 0.5 mM EGTA) 1mM PMSF 0.5% Triton X-100, 0.05% Deoxycholate 0.1% IGE-PAL, 1mM PMSF and protease inhibitor cocktail). 500μL aliquots of solubilized chromatin were clarified by centrifugation at 12,000g, and the supernatant was preincubated for 2 h with protein A agarose beads blocked with salmon sperm DNA and BSA. Precleared chromatin was incubated with 5 μL of anti-Rb antibody (Cell Signaling 4H1) or no antibody control at 4°C for 16 h. Immune complexes were bound to protein A agarose beads at 4°C for an additional 2h. The beads were washed two times with IP buffer, two times with high salt buffer (IP buffer with 0.5 M NaCl), 2 times with LiCl buffer (10 mM Tris at pH 8.0, 250 mM LiCl, 0.5% NP40, 1% Triton X-100, 5 mM EDTA), and two times with TE. To remove the crosslinks, the DNA–protein complexes were incubated in elution buffer (50 mM Tris (pH 8.0), 5 mM EDTA, and 1% SDS and 0.1 mg/mL proteinase K) for 6 hours at 65°. After 2 phenol-chloroform extractions of the eluate, the DNA was precipitated with 2.5 volumes of ethanol, 0.3M sodium acetate and 1μL of Pellet Paint as carrier at -20 overnight. Precipitated DNA was pelleted, washed once with 70% ethanol, dried, and resuspended in 100 μL of water.<br><br>*Illumina library construction and sequencing.*<br>We started with 30uL of IP DNA (out of a total of 100μl that was obtained from each IP). The DNA ends were repaired in a 50 μL reaction containing (30μl IP DNA, 5μL of 10X T4 DNA ligase buffer, 2μL of 10mM dNTP mix, 1 μL of T4 DNA polymerase (3U/μL), 1 μL of Klenow DNA polymerase (1U/μL) and 1 μL of T4 polynucleotide kinase (10U/μl); all from New England Biolabs) that was incubated at 20C for 30 minutes. The repaired DNA was extracted with phenol–chloroform–isoamyl alcohol (pH 8.0; 100 mul; Fisher) in 0.5-ml phase-lock gel tubes (heavy; Eppendorf) and precipitated with 2.5V of 100% EtOH, 0.3M sodium acetate and 1 μL of Pellet Paint (Novagen). We added a single adenine base to the DNA using a 50uL reaction containing (43μL of repaired DNA, 5μL of buffer, 1μL of 10mM dATP and 1 μL of 5U/μl Klenow 3' and 5' exo minus from New England Biolabs) that was incubated for 30 minutes at 37 °C. The illumina adaptors were ligated in a 30 μL reaction containing (23μL of modified DNA, 3 μl of buffer, 1 μL of 1/10 diluted adaptors, and 4 μL of DNA ligase from New England Biolabs) that was incubated at RT for 2 hours. The DNA was recovered using a QIAquick PCR Purification Kit (Qiagen) according to the manufacturer's instructions and using 30 μl of elution buffer. The adaptor-modified DNA fragments were enriched by PCR (denature for 30s at 98 followed by 15 cycles of 10s at 98, 30s at 65, 30s at 72 and a final extension at 72 for 5 minutes) using Phusion polymerase (Finnzymes) and PCR primer 1.1 and 2.1 (Illumina) following the manufacturer's instructions. The amplified DNA was separated on a 2% agarose gel and **products in the 150-300 bp range** were excised from the gel and purified using a QIAGEN Gel Extraction Kit. The DNA was quantified using the Nanodrop 7500 spectrophotometer. 10 nM DNA solution was used for cluster generation and 36 cycles of sequencing on the Illumina cluster station and 1G analyzer.	[13]
Chromatin Immune Precipitation. Cells were chemically cross-linked either on plate or in suspension by the addition of one-tenth volume of fresh 11% formaldehyde solution containing [1 mM EDTA, 0.5 mM EGTA, 100 mM NaCl, and 50 mM Hepes-KOH (pH 7.5)] for 15 min at room temperature. Cells were rinsed twice with 1× PBS, harvested using a silicon scraper or centrifuge, flash-frozen in liquid nitrogen, and stored at −80 °C before use. Typically 1–3 $\times 10^8$ cells were resuspended in 10 mL lysis buffer [50 mM Hepes- KOH (pH 7.5), 140 mM NaCl, 1 mM EDTA, 10% glycerol, 0.5% Nonidet P-40, and 0.25% Triton X-100] and rocked at 4 °C for 10 min. Cells were pelleted at 2880 × g on a tabletop centrifuge at 4 °C and resuspended in wash buffer [200 mM NaCl, 1 mM EDTA, 0.5 mM EGTA, and 10 mM Tris (pH 8.0)] and rocked at room temperature for 10 min. Cells were pelleted by spinning at 2880 × g/ 4 °C for 5 min and resuspended in 3 mL of sonication buffer [1 mM EDTA, 0.5 mM EGTA, 10 mM Tris (pH 8.0), 100 mM NaCl, 0.1% Na-deoxycholate, and 0.5% N-lauroylsarcosine]. We used a Mi- sonix Sonicator 3000 and sonicated at ≈20 W for 8 × 30-s pulses (60-s pause between pulses) at 4 °C for ES cells while samples were immersed in an ice bath. For adult tissues we sonicated six to seven rounds of 30-s pulses. Triton X-100 was added to the resulting whole-cell extract (1% end concentration), which was then cleared by centrifugation (20,800 × g at 4 °C), and supernatant was incubated overnight at 4 °C with 100 μL of Dynal Protein G magnetic beads that had been preincubated with 10 μg of the appropriate antibody for at least 3 h. Beads were washed five times with RIPA buffer [50 mM Hepes (pH 7.6), 1 mM EDTA, 0.7% deoxycholate, 1% Nonidet P-40, and 0.5 M LiCl] and once with Tris/EDTA buffer (TE) containing 50 mM NaCl. Bound complexes were eluted from the beads by heating at 65 °C with occasional vortexing in elution buffer [50 mM Tris (pH 8), 10 mM EDTA, and 1% SDS], and cross-linking was reversed by overnight incubation at 65 °C. After removal of the beads, immunoprecipitated DNA was diluted 1:1 with TE and then treated with RNase A (0.2 μg/μL final) for 2 h at 37 °C, followed by proteinase K (0.2 μg/μL final) treatment for 2 h at 50 °C. DNA was purified using two consecutive phenol:chloroform extractions using Phase Lock Gel tubes (5') and once using Qiagen PCR purification columns. The resulting DNA was either used for gene-specific PCR or further treated for analysis on the Solexa sequencer (GA2X genome sequencer) using the ChIP seq sample prep kit (1003473; Illumina) according to the manufacturer's protocol (11257047; Illumina), selecting **library fragments between 200 and 350 bp**. Samples were run by the Massachusetts Institute of Technology biopolymers facility (http://web.mit.edu/ki/facilities/biopolymers/index.html) using the GA2X genome sequencer (SCS v2.6, pipeline 1.5).	[14]
Control ($Stat3^{fl/fl}$) and $Stat3^{-/-}$ (Cd4 Cre; $Stat3^{fl/fl}$) naive $CD4^+CD44^-CD62L^+$ T cells were isolated and sorted on the FACSAria flow cytometer. Cells were cultured for 72 hr with CD3 (5 μg/ml) and CD28 (5 μg/ml), IL-6 (10 ng/ml), and TGF-β (2.5 ng/ml) with blocking antibodies to 10 μg/ml IL-2, 10 μg/ml IL-4, and 10 μg/ml IFN-γ. Cells were restimulated for 1 hr with IL-6 (10 ng/ml) and then processed for ChIP-Seq as previously described [83]. Antibodies against histone H3K4me3 (ab8580, Abcam) and phosphoserine (PS)-STAT3 (Cat. #9134, Cell Signaling) were used. The ChIP DNA fragments were blunt-ended, ligated to the illumina adaptors, and sequenced with the Illumina Genome Analyzer II.	[15]

Continued on next page

Table S3 – *Continued from previous page*

**Protocol**	**Study Reference**
ChIP and RNA isolation<br>Chromatin fixation and immunoprecipitation were performed essentially as described by [84]. Cells (1×10^9) were fixed in 200 mL of medium with 1% formaldehyde for 10 min at room temperature. Cross-linked cells were sonicated to produce **chromatin fragments of an average size of 200–400 bp**. Soluble chromatin was separated from insoluble material by centrifugation. The supernatant containing chromatin of 5×10^7 cells was used for immunoprecipitation. Psc and Ph antibodies were described by [85] and Pc and TRX-C antibodies were described by [86]. Anti-H3K4me3 was purchased from Millipore. RNA was isolated using TRIzol reagent (Invitrogen) following the manufacturer's instructions.<br><br>Preparation of ChIP-seq and mRNA-seq libraries<br>Sequencing libraries were prepared with the Illumina mRNA-Seq 8-Sample Prep kit and ChIP-Seq DNA Sample Prep kit according to Illumina's instructions. After adapter ligation, **library fragments of ∼250 bp** were isolated from an agarose gel. The DNA was PCR amplified with Illumina primers for 15 (RNA-seq) and 18 (ChIP-seq) cycles, purified, and loaded on an Illumina flow cell for cluster generation. Libraries were sequenced on the Genome Analyzer II and Genome Analyzer IIx following the manufacturer's protocols.	[16]
MCF-7 cells grown in hormone-depleted medium were treated with 17β–oestradiol ('oestrogen', E2) for 45 min before being crosslinked with 1% formaldehyde for 10 min. ChIA-PET libraries were constructed by first performing ChIP with HC-20 antibody (Santa Cruz) or Mab-NRF3A6-050 antibody (Diagenode) [76] against ER-α. [...] To characterize ER-α-bound interactions and associated genes functionally, we conducted gene expression microarray experiments in a time course with and without E2 treatment, and generated genome-wide maps of H3K4me3 (ab8580; Abcam) and RNAPII (serine-5 phosphorylation antibody, ab5131; Abcam) ChIP-Seq data by using Illumina GA single-read sequencing.	[17]
Chromatin immunoprecipitation<br>For each modified histone and Pol II ChIP experiment, we dissected approximately 200 pairs of bam testes in cold phosphate-buffered saline (PBS) and grouped them in 200 μl PBS that contained protease inhibitor (Roche complete mini, #11836153001, Nutley, NJ, USA) and 0.5 mM phenylmethanesulfonyl fluoride (PMSF; MP Biomedicals, #195381, Solon, OH, USA). Approximately 1,000 cells could be extracted from one bam testis. We then added 5.5 μl 37% fresh formaldehyde (Supelco, #47083-U, Bellefonte, PA, USA) and incubated at 37°C for 15 minutes. The testes were washed twice with 450 μl cold 1× PBS (with inhibitors and PMSF). Then 200 μl lysis buffer (50 mM Tris-HCl, pH7.6, 1 mM CaCl2, 0.2% Triton X-100, 5 mM butyrate, 1× protease inhibitor cocktail, and 0.5 mM fresh PMSF) was added and the tissues were homogenized thoroughly followed by incubation at room temperature for 10 minutes. **Chromatin was sheared into approximately 200-bp fragments by sonication** using Microtip (Misonix, Inc., Microson XL-2000, Farmingdale, NY, USA) with the following procedure: 4 s at power 20, rest for 50 s, 4 to 5 times, followed by spinning at 14k rpm for 10 minutes at 4°C. The chromatin was diluted 10× with RIPA buffer (10 mM Tris, pH7.6, 1 mM EDTA, 0.1% SDS, 0.1% Na-Deoxycholate, 1% Triton X-100, with protease inhibitors and PMSF) and 50 μl of this dilution was reverse cross-linked with 0.25 M NaCl for 2 hours at 65°C and used as input for real-time PCR analysis.<br>We washed 40 μl of Dynabeads Protein A (Invitrogen, #100.01D) with 600 μl 1× PBS. We then added 100 μl 1× PBS with 4 μg antibody and incubated the antibody-Protein A beads mixture at room temperature for 40 minutes with occasional tapping. After the unbound antibody was removed using the manipulator (Invitrogen, DYNAL MPC-S), 1 ml of the chromatin extract was added to the beads and the mixture was rotated at 4°C overnight. The beads were then washed twice with 1 ml RIPA buffer, twice with 1 ml RIPA buffer containing 0.3 M NaCl, once with LiCl wash buffer (0.25 M LiCl, 0.5% NP40, and 0.5% sodium deoxycholate), once with 1 ml TE (10 mM Tris-HCl, pH 8.0 and 1 mM EDTA) containing 0.2% Triton X-100, and once with 1 ml TE. The beads were then suspended in 100 μl 1× TE containing 3 μl 10% SDS and 5 μl 20 mg/ml proteinase K, followed by incubation at 65°C overnight. After the supernatant was collected, the beads were washed once more with 100 μl TE with 0.5 M NaCl. The supernatant from this wash was combined with the previous supernatant. The combined samples were treated by Phenol/Chloroform extraction, salt/EtOH precipitation, and dissolved in 50 μl 1× TE. The products were either used for real-time PCR analyses or processed for Solexa sequencing according to the established protocol. Antibodies used include those against H3K4me3 (Abcam, #ab8580, Cambridge, MA, USA), H3K27me3 (Millipore, #07-449, Billerica, MA, USA), H3K36me3 (Abcam, #ab9050), H3 (Abcam, #ab1791) and RNA Pol II (Abcam, ab5408).<br><br>ChIP experiment using S2 cells<br>Exponentially growing S2 cells were harvested and dissolved in digestion buffer (50 mM Tris-HCl, pH7.6, 1 mM CCl2, 0.2% Triton X-100, 5 mM butyrate, 1× protease inhibitor cocktail and 0.5 mM PMSF). Chromatin was prepared and ChIP-seq experiments were performed as described previously [5] with antibodies against Pol II (Abcam, #ab5408), H3K4me3 (Abcam, #ab8580), H3K27me3 (Millipore/Upstate, #07-449), and H3K36me3 (Abcam, #ab9050).<br><br>Solexa pipeline analysis<br>The 25-bp sequencing reads were obtained from the Illumina Genome Analyzer pipeline.	[18]
Preparation of chromatin for ChIP from mixed sex embryos (ChIP) or sexed larvae (ChIP and ChIP-seq) was performed as described previously [87,88]. Commercial antibodies used for ChIP were from Abcam (H3K36me3, ab9050; H3K4me2, ab7766; H3, ab1791; RNA polymerase II (4H8), ab5408) and Upstate Biotechnologies (H4K16ac, 07-329). Quantitation of ChIP DNA (three biological replicates) by qPCR (in duplicates) was done as described previously [87]. Error bars correspond to standard deviations among three independent experiments. Sequences of real-time PCR primers used can be found in Supplemental Table 1. To prepare large batches of chromatin from TrojanElephant; MSL3-TAP; msl31 mixed sex larvae (second and third instars), the animals were separated from food and pupae by washing in 20% sucrose. Up to 20 g of larvae were used per chromatin prep. For ChIP-seq, ∼10,000 TrojanElephant/Y; MSL3-TAP; msl31 male larvae were collected.	[19]

Continued on next page

Table S3 – *Continued from previous page*

**Protocol**	**Study Reference**
Chromatin immunoprecipitation<br>Protocols describing chromatin immunoprecipitation (ChIP) materials and methods can be downloaded from http://web.wi.mit.edu/young/hES_PRC and have previously been described in detail [89].<br>Human ES, iPS or fibroblast cells were grown to a final count of $\sim 5 \times 10^7$ cells to obtain starting material for six chromatin immunoprecipitations. Cells were chemically cross-linked by the addition of one-tenth volume of fresh 11% formaldehyde solution for 15 minutes at room temperature. Cells were rinsed twice with 1X PBS, harvested by centrifugation, and flash frozen in liquid nitrogen. Cross-linked cells were stored at -80°C prior to use.<br>Cells were re-suspended, lysed and sonicated to solubilize and shear cross-linked DNA. Sonication was performed using a Misonix Sonicator 3000 at a power of 27W for ten 30 second pulses with a 90 second pause between each pulse. Samples were kept on ice at all times.<br>The resulting whole cell extract was incubated overnight at 4 degrees C with 10μl of Dynal Protein G magnetic beads that had been pre-incubated with approximately 3 μg of the appropriate antibody. Each individual immunoprecipitation used 1/6 of the 3ml total, or $\sim 8 \times 10^6$ cells per IP. The immunoprecipitation was allowed to proceed overnight. Beads were washed three times (3 x 1.5ml) with RIPA buffer and one time (1x 1.5ml) with TE containing 50 mM NaCl. Bound complexes were eluted from the beads by heating at 65 degrees C with occasional vortexing and cross-linking was reversed by overnight incubation at 65 degrees C. Whole cell extract DNA (reserved from the sonication step) was also treated for cross-link reversal. Immunoprecipitated DNA and whole cell extract DNA were then purified by treatment with RNAse A, proteinase K and two phenol:chloroform:isoamyl alcohol extractions.<br>The ChIP antibodies used were ab8580 (Abcam) for H3K4me3 and ab6002 (Abcam) for H3K27me3.<br><br>ChIP-Seq sample preparation<br>All protocols for Solexa sample preparation and sequencing are provided by Illumina (http://www.illumina.com/). A brief summary of the technique, minor protocol modifications, and data analysis methods are described below.<br>Purified ChIP DNA was prepared for sequencing according to a modified version of the Illumina/Solexa Genomic DNA protocol. Approximately 50-200ng of IP DNA was prepared for ligation of Solexa linkers by repairing the ends and adding a single adenine nucleotide overhang to allow for directional ligation. A 1:100 dilution of the Adaptor Oligo Mix (Illumina) was used in the ligation step. A subsequent PCR step with 18 amplification cycles added additional linker sequence to the fragments to prepare them for annealing to the Genome Analyzer flow-cell. Amplified material was purified by Qiaquick MinElute (Qaigen) and a narrow range of fragment sizes was selected by separation on a 2% agarose gel and **excision of a band between 150-300 bp, representing IP fragments between 50 and 200nt in length and ∼100bp of primer sequence**. The DNA was purified from the agarose and diluted to 10 nM for loading on the flow cell.<br><br>Solexa sequencing<br>The DNA library (2-4 pM) was applied to one lane of the flow-cell (eight samples per flow-cell) using a Cluster Station device (Illumina). The concentration of library applied to the flow-cell was calibrated so that polonies generated in the bridge amplification step originate from single strands of DNA. Multiple rounds of amplification reagents were flowed across the cell in the bridge amplification step to generate polonies of approximately 1,000 strands in 1μm diameter spots. Double stranded polonies were visually checked for density and morphology by staining with a 1:5000 dilution of SYBR Green I (Invitrogen) and visualizing with a microscope under fluorescent illumination. Validated flow-cells were stored at 4 degrees C until sequencing.<br>Flow-cells were removed from storage and subjected to linearization and annealing of sequencing primer on the Cluster Station. Primed flow-cells were loaded into the Genome Analyzer 1G (Illumina). After the first base was incorporated in the sequencing-by-synthesis reaction the process was paused for a key quality control checkpoint. A small section of each lane was imaged and the average intensity value for all four bases was compared to minimum thresholds. Flow-cells with low first base intensities were re-primed and if signal was not recovered the flow-cell was aborted. Flow-cells with signal intensities meeting the minimum thresholds were resumed and sequenced.<br>Images acquired from the Genome Analyzer were processed through the bundled image extraction pipeline (Illumina), which identified polony positions, performed base-calling and generated QC statistics.	[20]
For each islet ChIP experiment, islets from at least 10 adult (8–10 wk old) ICR (for transcription factor libraries) or C57BL6/J (for histone methylation libraries) mice were purified by collagenase digestion and gradient centrifugation, with subsequent hand picking. Perfused liver or purified E14.5 hepatocytes were obtained from C57BL6/J, and ChIP was performed as described [90] using 3 μg of anti-FOXA2 (Santa Cruz), anti-PDX1 (Upstate), anti-HNF4A (Santa Cruz), anti-H3K4me1 (Santa Cruz), anti-H3K4me3 (Santa Cruz), or normal rabbit IgG (Santa Cruz). DNA from triplicate pooled ChIP experiments was purified by 8% PAGE to obtain 100–300-bp fragments and sequenced on an Illumina 1G sequencer as previously reported [91]	[21]
ChIP DNA was amplified as described [92]	[22]
Jurkat cells were treated as described above and ChIP-seq was performed as recently described [92]. Immunoprecipitation was performed with the murine monoclonal antibody 4H8 (Abcam), which recognizes the C-terminal domain of Pol II. The immunoprecipitated material was end-repaired, A-tailed, ligated to sequencing adapters, amplified using 18 cycles of PCR, and **size-selected (200–300 bp)** material was subject to single-end sequencing using an Illumina Genome Analyzer, as per the manufacturers' recommendations.	[23]
Chromatin immunoprecipitations were performed as per standard protocols (Upstate) [93]. Briefly, cells were treated with either vehicle or 100 nM dexamethasone for 1 h. Cells were crosslinked for 10 min at 37 $^{\circ}$C in 1% formaldehyde followed by a quenching step for 10 min with 150 mM glycine. A single chromatin immunoprecipitation contained 400 μg of sonicated, soluble chromatin and a cocktail of antibodies to the glucocorticoid receptor (7.5 μg of PA1-511A antibody, ABR, 15 μg of MA1-510 antibody, ABR and 3 μg of sc-1004; Santa Cruz). The ChIP reaction was scaled $\times 5$ for ChIP-seq. DNA isolates from immunoprecipitates were used as templates for real-time quantitative PCR amplification or sequenced as described below. All ChIP experiments were performed at least two times.	[24]
ChIP-seq and data analysis was performed essentially as described in [94]. Briefly, proteins were cross-linked to DNA by incubation of cells for 10 min in medium containing 1% formaldehyde at room temperature, after which the cross-linking was quenched, nuclei were extracted, and DNA was fragmented by sonication. Precleared samples were incubated with 4 μg of specific antibody overnight at 4°C, and the antibodies were collected by incubation with 30 μL of protein G–Sepharose beads for 2–3 h at +4°C followed by centrifugation at 800g. Subsequently, the beads were washed, precipitated chromatin complexes were eluted, and the cross-links were reversed by incubating overnight at 65°C. DNA was extracted with a QiaQuick PCR purification kit (QIAGEN). ChIP DNA was quantitated by PicoGreen dsDNA quantitation reagent (Molecular Probes). A ChIP library was prepared for sequencing as described in [94], and **$120-350$ bp fragments were size-selected on a 2% agarose gel**. The fragments were enriched by 18 cycles of PCR amplification and **size-selected again (to $150-300$ bp)**. Purified DNA (QIAGEN gel purification kit) was quantified (Nanodrop 1000 spectrophotometer) and used for massively parallel sequencing (Illumina Genome Analyzer) according to the manufacturer's instructions.	[25]

Continued on next page

Table S3 – *Continued from previous page*

Protocol	Study Reference
Hb9::GFP ES cells were differentiated as previously described [95]. ES cells were trypsinized and seeded at 5×10^5 cells/ml in ANDFK medium (Advanced DMEM/F12:Neurobasal (1:1) Medium, 10% Knockout-SR, Pen/Strep, 2 mM L-Glutamine, and 0.1 mM 2-mercaptoethanol) to initiate formation of embryoid bodies (Day 0). Medium was exchanged on Days 1, 2 and 5 of differentiation. Patterning of embryoid bodies was induced by supplementing media on Day 2 with 1 μM all-trans-Retinoic acid (Sigma) and 0.5 μM agonist of hedgehog signaling (SAG, Calbiochem). For ChIP experiments, the same conditions were used but scaled to seed 1×10^7 cells on Day 0. An inducible Hoxc9-V5 tagged line was made by inserting tagged Hoxc9 in the LoxP site of the Ainv15 cell line (ATCC SCRC-1029).<br>Approximately 6×10^7 cells taken at Day 5 of differentiation were cross-linked using formaldehyde and snap-frozen in liquid nitrogen. Cells were thawed on ice, resuspended in 5ml lysis buffer 1 and mixed on a rotating platform at 4°C for 5 minutes. Samples were spun down for 3 minutes at 3000rpm, resuspended in 5ml lysis buffer 2 and mixed on a rotating platform for 5 minutes at room temperature. Samples were spun down once more, resuspended in lysis buffer 3 and sonicated using a Misonix 3000 model sonicator to shear cross-linked DNA to an average fragment size of approximately 500bp. Triton X-100 was added to the lysate after sonication to final concentrations of 1% and the lysate spun down to pellet cell debris. The resulting whole-cell extract supernatant was incubated on a rotating mixer overnight at 4°C with 100 μl of Dynal Protein G magnetic beads that had been preincubated for 24 hours with 10 μg of anti V5 antibody (Abcam, ab15828) antibody in a PBS/BSA solution. After incubation, the beads were washed and the protein-DNA complexes were eluted at 65°C for 15 minutes. Cross-links were reversed by an overnight incubation at 65°C. samples were then treated with RNase A and Proteinase K to remove proteins and contaminating nucleic acids, and the DNA fragments precipitated with cold EtOH.<br>The enriched DNA was prepared to sequence using the ChIP-seq sample Prep kit from Illumina and sequenced according to manufacturer specifications.	[26]

Continued on next page

Table S3 – *Continued from previous page*

Protocol	Study Reference
Biological replicates of all ChIP-Seq datasets with the exception of Mediator (Med12 and Med1) in MEFs, were generated and combined for analysis. A summary of the ChIP-Seq data is contained within Supplementary Table 6.<br>For Med1 (CRSP1/TRAP220) occupied genomic regions, we performed ChIP- Seq experiments using Bethyl Laboratories (A300-793A) antibody. The affinity purified antibody was raised in rabbit against an epitope corresponding to amino acids 1523-1281 mapping at the C-terminus of human Med1.<br>For Med12 occupied genomic regions, we performed ChIP-Seq experiments using Bethyl Laboratories (A300-774A) antibody. The affinity purified antibody was raised in rabbit against an epitope corresponding to amino acids 2150-2212 mapping at the C-terminus of human Med12.<br>For Smc1a occupied genomic regions, we performed ChIP-Seq experiments using Bethyl Laboratories (A300-055A) affinity purified rabbit polyclonal antibody. The epitope recognized by A300-055A maps to a region between residue 1175 and the C-terminus of human Smc1a.<br>For Smc3 occupied genomic regions, we performed ChIP-Seq experiments using Abcam (ab9263) antibody. The affinity purified antibody was raised in rabbit against an epitope corresponding to the last 100 amino acids of the human Smc3 protein.<br>For TBP occupied genomic regions, we performed ChIP-Seq experiments using Abcam (ab818) antibody. The antibody was raised with a synthetic peptide which represents amino acid residues 1-20 of human TBP.<br>For Pol2 occupied genomic regions, we performed ChIP-Seq experiments using Covance 8WG16 antibody. This mouse monoclonal antibody was raised against the C-terminal heptapeptide repeat region on the largest subunit of Pol2, purified from wheat germ extract.<br>For H3K79me2 occupied genomic regions, we performed ChIP-Seq experiments using Abcam ab3594 rabbit polyclonal antibody. The antibody was raised with a synthetic peptide that is within residues 50 to the C-terminus of Human Histone H3, di methylated at K79.<br>For CTCF occupied genomic regions, we performed ChIP-Seq experiments using an Upstate 07-729 rabbit polyclonal antibody.<br>For Nipbl occupied genomic regions, we performed ChIP-Seq experiments using a Bethyl A301-779A rabbit polyclonal antibody. The affinity purified antibody was raised in rabbit to a region between amino acid residues 1025 and 1075 of human Nipbl.<br>Protocols describing chromatin immunoprecipitation materials and methods have been previously described [96].<br>Embryonic stem cells or MEFs were grown to a final count of 5-10 x 10^7 cells for each ChIP experiment. Cells were chemically crosslinked by the addition of one-tenth volume of fresh 11% formaldehyde solution for 15 minutes (Esc cells) or 10 minutes (MEFs) at room temperature. Cells were rinsed twice with 1X PBS and harvested using a silicon scraper and flash frozen in liquid nitrogen. Cells were stored at -80°C prior to use. Cells were resuspended, lysed in lysis buffers and sonicated to solubilize and shear crosslinked DNA. Sonication conditions vary depending on cells, culture conditions, crosslinking and equipment.<br>For Nipbl, Smc1a, Smc3, Pol2, H3K79me2 and Med1 the sonication buffer was 20mM Tris-HCl pH8, 150mM NaCl, 2mM EDTA, 0.1% SDS, 1% Triton X-100. We used a Misonix Sonicator 3000 and sonicated at approximately 24 watts for 10 x 30 second pulses (60 second pause between pulses). Samples were kept on ice at all times. The resulting whole cell extract was incubated overnight at 4°C with 100 μl of Dynal Protein G magnetic beads that had been pre-incubated with approximately 10 μg of the appropriate antibody. Beads were washed 1X with the sonication buffer, 1X with 20mM Tris-HCl pH8, 500mM NaCl, 2mM EDTA, 0.1% SDS, 1%Triton X-100, 1X with 10mM Tris-HCl pH8, 250nM LiCl, 2mM EDTA, 1% NP40 and 1X with TE containing 50 mM NaCl.<br>For Med12 and CTCF, the sonication buffer was 10mM Tris-HCl pH8, 100mM NaCl, 1mM EDTA, 0.5mM EGTA, 0.1% Na-Deoxycholate, 0.5% N- lauroylsarcosine. We used the same sonication and wash conditions as described above.<br>For TBP, the sonication buffer was 10mM Tris-HCl pH8, 100mM NaCl, 1mM EDTA, 0.5 mM EGTA, 0.1% Na-Deoxycholate and 0.5% N-lauroylsarcosine. We used a Misonix Sonicator 3000 and sonicated at approximately 24 watts for 10 x 30 second pulses (60 second pause between pulses). After Sonication, 10% Triton-X was added. After immunoprecipitation, beads were washed 4X with the RIPA buffer (50 mM Hepes-KOH pH 7.6, 500 mM LiCl, 1 mM EDTA, 1% NP40 and 0.7% Na-Deoxycholate) and 1X with TE containing 50 mM NaCl.<br>Bound complexes were eluted from the beads (50 mM Tris-Hcl, pH 8.0, 10 mM EDTA and 1% SDS) by heating at 65°C for 1 hour with occasional vortexing and crosslinking was reversed by overnight incubation at 65°C. Whole cell extract DNA reserved from the sonication step was also treated for crosslink reversal.<br>ChIP-Seq Sample Preparation and Analysis<br>All protocols for Illumina/Solexa sequence preparation, sequencing and quality control are provided by Illumina (http://www.illumina.com/pages.ilmn?ID=203). A brief summary of the technique and minor protocol modifications are described below.<br><br>Sample Preparation<br>DNA was prepared for sequencing according to a modified version of the Illumina/Solexa Genomic DNA protocol. Fragmented DNA was prepared for ligation of Solexa linkers by repairing the ends and adding a single adenine nucleotide overhang to allow for directional ligation. A 1:100 dilution of the Adaptor Oligo Mix (Illumina) was used in the ligation step. A subsequent PCR step with limited (18) amplification cycles added additional linker sequence to the fragments to prepare them for annealing to the Genome Analyzer flow-cell. After amplification, a narrow range of fragment sizes was selected by separation on a 2% agarose gel and **excision of a band between 150-350 bp (representing shear fragments between 50 and 250nt in length and ∼100bp of primer sequence)**. The DNA was purified from the agarose and diluted to 10 nM for loading on the flow cell.<br>Polony Generation and Sequencing<br>The DNA library (2-4 pM) was applied to the flow-cell (8 samples per flow-cell) using the Cluster Station device from Illumina. The concentration of library applied to the flow-cell was calibrated such that polonies generated in the bridge amplification step originate from single strands of DNA. Multiple rounds of amplification reagents were flowed across the cell in the bridge amplification step to generate polonies of approximately 1,000 strands in 1μm diameter spots. Double stranded polonies were visually checked for density and morphology by staining with a 1:5000 dilution of SYBR Green I (Invitrogen) and visualizing with a microscope under fluorescent illumination. Validated flow-cells were stored at 4°C until sequencing.<br>Flow-cells were removed from storage and subjected to linearization and annealing of sequencing primer on the Cluster Station. Primed flow-cells were loaded into the Illumina Genome Analyzer 1G. After the first base was incorporated in the Sequencing-by-Synthesis reaction the process was paused for a key quality control checkpoint. A small section of each lane was imaged and the average intensity value for all four bases was compared to minimum thresholds. Flow-cells with low first base intensities were re-primed and if signal was not recovered the flow-cell was aborted. Flow-cells with signal intensities meeting the minimum thresholds were resumed and sequenced for 26 or 32 cycles.<br>ChIP-Seq Data Analysis<br>Images acquired from the Illumina/Solexa sequencer were processed through the bundled Solexa image extraction pipeline, which identified polony positions, performed base-calling and generated QC statistics.	[27]

Continued on next page

Table S3 – *Continued from previous page*

Protocol	Study Reference
ChIP Seq. Chromatin immunoprecipitation for NFκB was carried out as previously described [97,98]. Biological replicates were grown in separate batches and at separate times. $3-5$ different cell lines were grown in parallel at any given time. Briefly, 2×10^8 cells were grown to a density of $0.6-0.8 \times 10^6$/mL in 15% fetal bovine serum and treated with 25 ng/mL human recombinant TNF-alpha (eBioscience #14-8329, San Diego, CA) for six hours at 37°C, 5% CO2. After stimulation, cells were cross-linked in 1% formaldehyde for 10 minutes at room temperature. Nuclear lysates were sonicated using a Branson 250 Sonifier (power setting 2, 100% duty cycle for 7×30-s intervals), such that the chromatin fragments ranged from 50-2000kb. Clarified lysates were divided in half and treated overnight at four degrees Celsius with 8μg of either anti-NFκB p65 (C-20) rabbit polyclonal antibody or normal rabbit IgG (Santa Cruz Biotechnology Sc-2027, Santa Cruz, CA). Protein-DNA complexes were captured on Protein A agarose beads (Millipore #16-156, Billerica, MA) and eluted in 1% SDS TE buffer at 65°C. Following cross-link reversal and purification, the ChIP DNA sequencing libraries were generated according to Illumina DNA Sample Kit Instructions (Illumina Part # 0801– 0303, San Diego, CA). The protocol was modified such that enzymes were obtained from other suppliers, as described [99]. Libraries were sequenced on an Illumina Genome Analyzer II. PolII ChIP DNA and libraries were prepared as described for NFκB, except that cells were not stimulated with TNF-alpha. DNA was immunoprecipitated with either 24μg of mouse monoclonal 8WG16 antibody (Covance MMS-126R, Princeton, NJ) or normal mouse IgG (Millipore #12-371).	[28]
ChIP-seq library preparation and RRBS: After necessary treatments, approximately 500K MEF cells were crosslinked with 1% formaldehyde for 10 minutes at 370 C. After quenching with glycine for 5 min, the cells were washed twice with ice cold PBS with 10% serum. Cell pellets were re-suspended in 100 ml of lysis buffer (1% SDS, 10mM EDTA, 50mM Tris-HCl, pH 8.1) and incubated on ice for 10 min. The lysate was then diluted with 400 ml of ChIP dilution buffer containing (0.01% SDS, 1.1% Triton X-100, 1.2mM EDTA, 16.7mM Tris-HCl, pH 8.1)). Chromatin was sonicated for 3.5 min using a Branson 250 at 40 % power amplitude (pulses: 0.7 second "on", and 1.3 second "off"). The fragmented chromatin was then immunoprecipitated overnight in a total volume of 1 ml ChIP Dilution buffer containing protease inhibitor cocktails (Roche), using: 1 mg/ml K4me1 (Abcam ab8895), 1 mg/ml K4me2 (Abcam ab7766), 1 mg/ml K4me3 (Millipore 07473), 2 mg/ml K27me3 (Millipore 07449) or 1 mg/ml K36me3 (Abcam ab9050) antibody. Next, the samples were incubated with $\sim$10 ml of pre-washed Protein A-Sepharose beads at 4 deg C for 2 hours. We then collected the beads by brief centrifugation at 1,000 x g, keeping the unbound fraction to check chromatin fragmentation. Then, the beads were washed twice with 700 ml of each of the following buffers at 4 deg C: Low Salt Immune Complex Wash Buffer (0.1% SDS, 1% Triton X-100, 2mM EDTA, 20mM Tris-HCl, pH 8.1, 150mM NaCl); LiCL wash buffer (0.25M LiCl, 1% NP40, 1% deoxycholate, 1mM EDTA, 10mM Tris-HCl, pH 8.1); and TE (10mM Tris-HCl, 1mM EDTA, pH 8.0). We used filter columns (Costar 8160) in order to minimize the beads and sample loss during washes. DNA was then eluted from the beads twice in 125 ml of Chip Elution Buffer (0.2 % SDS, 0.1 M NaHCO3 supplemented with fresh 5 mM DTT) by incubation at 65 deg C for 10 min. The eluted chromatin and the "input" sample were then incubated at 65 deg C for 5 hrs and Proteinase K digested at 37 deg C for 2 hours. The ChIP DNA was recovered by phenol-chloroform extraction and ethanol precipitation. After validating the ChIP enrichments in the precipitated DNA, ChIP DNA was processed into Illumina sequencing libraries, as described before.	[29]
Third instar larvae were homogenized in PBS followed by crosslinking with 1% formaldehyde. Crosslinked chromatin was fragmented by sonicating four times for eight minutes (high power, 30 seconds on/off) with a Bioruptor (Diagenode). Chromatin immunoprecipitation was performed with anti-H3K9me2 antibodies (07-441, Upstate) and Prot A/G beads (Santa Cruz) were used to capture antibody bound chromatin. End repair was performed on 10-20 ng of enriched ChIPed DNA (measured by Qubit fluorometer) using the Quant-iT dsDNA HS Assay Kit from (Invitrogen, Q32851). Adaptors were ligated to DNA fragments, which were **subsequently size selected ($\sim$300 bp)**. The adapter-modified DNA fragments were subjected to limited PCR amplification (14 cycles) and quality control was made by qPCR, as well as by running the PCR products on a Bioanalyzer (BioRad). Finally, cluster generation and sequencing-by-synthesis (36 bp) was performed using the Illumina Genome Analyzer IIx according to standard protocols of the manufacturer (Illumina). The image files generated by the Genome Analyzer were processed to extract DNA sequence data.	[30]
ChIP assays were performed as described previously [100]. Briefly, GM06990 cells cultured for 72h were cross-linked with 1% formaldehyde and incubated for 7min at room temperature. Formaldehyde was deactivated by the addition of glycine (125mM final concentration). **Sonicated cell lysate containing an average size of 500bp** DNA fragments was used for immunoprecipitation to enrich E2F4-DNA complexes using an anti-E2F4 antibody (SC-1082X, Santa Cruz Biotech). Immunoprecipitated DNA was sequenced using Illumina sequencing technology (single end sequencing).	[31]
ChIP-Seq for Ldb1, Scl, GATA-2 and immunoglobulin G (control) was done as described [5]	[32]

Continued on next page

Table S3 – *Continued from previous page*

**Protocol**	**Study Reference**
ChIP protocols were adapted from the Young Lab ChIP protocol (http://jura.wi.mit.edu/young_public/hESregulation/ChIP.html). Descriptions of these protocol modifications have been previously published [101]. Briefly, approximately 6 × 10e7 cells taken from each developmental time point were cross-linked using formaldehyde and snap-frozen in liquid nitrogen. Cells were thawed on ice, resuspended in 5 ml lysis buffer 1 (50 mM Hepes-KOH, pH 7.5, 140 mM NaCl, 1 mM EDTA, 10% glycerol, 0.5% NP-40, 0.25% Triton X-100) and mixed on a rotating platform at 4°C for 5 minutes. Samples were spun down for 3 minutes at 3,000 rpm, resuspended in 5 ml lysis buffer 2 (10 mM Tris-HCl, pH 8.0, 200 mM NaCl, 1 mM EDTA, 0.5 mM EGTA), and mixed on a rotating platform for 5 minutes at room temperature. Samples were spun down once more, resuspended in lysis buffer 3 (10 mM Tris-HCl, pH 8.0, 100 mM NaCl, 1 mM EDTA, 0.5 mM EGTA, 0.1% Na-deoxycholate, 0.5% N-lauroylsarcosine) and sonicated using a Misonix 3000 model sonicator to **sheer cross-linked DNA to an average fragment size of approximately 500 bp**. Triton X-100 was added to the lysate after sonication to final concentrations of 1% and the lysate spun down to pellet cell debris. The resulting whole-cell extract supernatant was incubated on a rotating mixer overnight at 4°C with 100 μl of Dynal Protein G magnetic beads that had been preincubated for 24 hours with 10 μg of the appropriate antibody in a phosphate-buffered saline/bovine serum albumin solution. Pan-RAR (Santa Cruz Biotechnology, Santa Cruz, CA, USA, sc-773), Pol2-S5P (Abcam, Cambridge, UK, ab5131), and Pol2-S2P (Abcam, H5 clone ab24758) antibodies were used for ChIP experiments. After approximately 16 hours of bead-lysate incubation, beads were collected with a Dynal magnet. ChIP samples probing for TF binding were washed with the following regimen, mixing on a rotating mixer at 4°C for 5 minutes per buffer: low-salt buffer (20 mM Tris at pH 8.1, 150 mM NaCl, 2 mM EDTA, 1% Triton X-100, 0.1% SDS), high-salt buffer (20 mM Tris at pH 8.1, 500 mM NaCl, 2 mM EDTA, 1% Triton X-100, 0.1% SDS), LiCl buffer (10 mM Tris at pH 8.1, 250 mM LiCl, 1 mM EDTA, 1% deoxycholate, 1% NP-40), and TE containing 50 mM NaCl. ChIP samples probing for histone and chromatin marks were washed four times with RIPA buffer (50 mM Hepes-KOH, pH 7.6, 500 mM LiCl, 1 mM EDTA, 1% NP-40, 0.7% Na-deoxycholate) and then once with TE containing 50 mM NaCl, again mixing on a rotating mixer at 4°C for 5 minutes per buffer. After the final bead wash, samples were spun down to collect and discard excess wash solution, and bound antibody-protein-DNA fragment complexes were eluted from the beads by incubation in elution buffer at 65°C with occasional vortexing. Cross-links were reversed by overnight incubation at 65°C. Samples were digested with RNase A and Proteinase K to remove proteins and contaminating nucleic acids, and the DNA fragments precipitated with cold ethanol. Purified DNA fragments were processed according to a modified version of the Illumina/Solexa sequencing protocol (http://www.illumina.com/systems/genome_analyzer_iix.ilmn)	[33]
Protocols describing all materials and methods have been previously described [89] and can be downloaded from http://web.wi.mit.edu/young/hES_PRC.<br>Briefly, we performed independent immunoprecipitations for each analysis. Embryonic stem cells were grown to a final count of $5 \times 10^{7} - 1 \times 10^{8}$ cells for each location analysis experiment. Cells were chemically crosslinked by the addition of one-tenth volume of fresh 11% formaldehyde solution for 15 minutes at room temperature. Cells were rinsed twice with 1xPBS and harvested using a silicon scraper and flash frozen in liquid nitrogen. Cells were stored at -80°C prior to use.<br>Cells were resuspended, lysed in lysis buffers and sonicated to solubilize and shear crosslinked DNA. Sonication conditions vary depending on cells, culture conditions, crosslinking and equipment. We used a Misonix Sonicator 3000 and sonicated at approximately 28 watts for 10 x 30 second pulses (90 second pause between pulses). For ChIP of Oct4, Nanog, Tcf3 and Suz12 in murine ES cells, SDS was added to lysate after sonication to a final concentration of 0.1%. Samples were kept on ice at all times.<br>The resulting whole cell extract was incubated overnight at 4°C with 100 μl of Dynal Protein G magnetic beads that had been pre-incubated with approximately 10 μg of the appropriate antibody. Beads were washed 4-5 times with RIPA buffer and 1 time with TE containing 50 mM NaCl. For ChIP of Oct4, Nanog, Tcf3 and Suz12 in murine ES cells, the following 4 washes for 4 minutes each were used instead of RIPA buffer: 1X low salt (20mM Tris pH 8.1, 150mM NaCl, 2mM EDTA, 1% Triton X-100, 0.1% SDS), 1X high salt (20mM Tris pH 8.1, 500mM NaCl, 2mM EDTA, 1% Triton X-100, 0.1% SDS), 1X LiCl (10mM Tris pH 8.1, 250mM LiCl, 1mM EDTA, 1% deoxycholate, 1% NP-40), and 1X TE+ 50mM NaCl. Bound complexes were eluted from the beads by heating at 65°C with occasional vortexing and crosslinking was reversed by overnight incubation at 65°C. Whole cell extract DNA (reserved from the sonication step) was also treated for crosslink reversal.<br>All protocols for Illumina/Solexa sequence preparation, sequencing and quality control are provided by Illumina (http://www.illumina.com/pages.ilmn?ID=203). A brief summary of the technique and minor protocol modifications are described below.<br>Sample Preparation<br>Immunoprecipitated (ChIP) DNA was prepared for sequencing according to a modified version of the Illumina/Solexa Genomic DNA protocol. Fragmented DNA was prepared for ligation of Solexa linkers by repairing the ends and adding a single adenine nucleotide overhang to allow for directional ligation. A 1:100 dilution of the Adaptor Oligo Mix (Illumina) was used in the ligation step. A subsequent PCR step with limited (18) amplification cycles added additional linker sequence to the fragments to prepare them for annealing to the Genome Analyzer flow-cell. After amplification, a narrow range of fragment sizes was selected by separation on a 2% agarose gel and **excision of a band between 150-300 bp (representing shear fragments between 50 and 200nt in length and ~100bp of primer sequence**). The DNA was purified from the agarose and diluted to 10 nM for loading on the flow cell.<br>Polony generation on Solexa Flow-Cells<br>The DNA library (2-4 pM) was applied to the flow-cell (8 samples per flow-cell) using the Cluster Station device from Illumina. The concentration of library applied to the flow-cell was calibrated such that polonies generated in the bridge amplification step originate from single strands of DNA. Multiple rounds of amplification reagents were flowed across the cell in the bridge amplification step to generate polonies of approximately 1,000 strands in 1μm diameter spots. Double stranded polonies were visually checked for density and morphology by staining with a 1:5000 dilution of SYBR Green I (Invitrogen) and visualizing with a microscope under fluorescent illumination. Validated flow-cells were stored at 4°C until sequencing.<br><br>Sequencing<br>Flow-cells were removed from storage and subjected to linearization and annealing of sequencing primer on the Cluster Station. Primed flow-cells were loaded into the Illumina Genome Analyzer 1G. After the first base was incorporated in the Sequencing-by-Synthesis reaction the process was paused for a key quality control checkpoint. A small section of each lane was imaged and the average intensity value for all four bases was compared to minimum thresholds. Flow-cells with low first base intensities were re-primed and if signal was not recovered the flow-cell was aborted. Flow-cells with signal intensities meeting the minimum thresholds were resumed and sequenced for 26 cycles.	[34]
Native ChIP, T7 in vitro amplification, RNA-chip, and the MEFF and MEFB1 cell lines were described previously [102]. [. . .] Sequencing libraries were obtained from 10 ng of ChIP DNA by adaptor ligation, gel purification and 18 cycles of PCR. Sequencing was carried out using the Illumina Genome Analyzer (GA) I system according to the manufacturer's protocol.	[35]

Continued on next page

Table S3 – *Continued from previous page*

**Protocol**	**Study Reference**
Chromatin immunoprecipitation (ChIP), sequential ChIP, FAIRE and antibodies<br>ChIP assays were performed from approximately 10^7 hESCs or hNECs per experiment, according to previously described protocol with slight modifications [103]. Briefly, cells were crosslinked with 1% formaldehyde for 10 min at room temperature and formaldehyde was quenched by addition of glycine to a final concentration of 0.125 M. **Chromatin was sonicated to an average size of 0.5–2 kb**, using Bioruptor (Diagenode). A total of 3–5 μg of antibody was added to the sonicated chromatin and incubated overnight at 4 °C. 10% of chromatin used for each ChIP reaction was kept as input DNA. Subsequently, 75 μl of protein A or protein G Dynal magnetic beads (depending of antibody species and Ig isotype) were added to the ChIP reactions and incubated for four additional hours at 4 °C. Magnetic beads were washed and chromatin eluted, followed by reversal of the crosslinkings and DNA purification. Resultant ChIP DNA was dissolved in water. [...]<br>ChIP-seq<br>Libraries were prepared from: hESC and hNEC p300 ChIP, hESC BRG1 ChIP, hESC FAIRE, hESC and hNEC H3K4me3 ChIP, hESC and hNEC H3K4me1 ChIPs, hESC and hNEC H3K27me3 ChIPs, hESC and hNEC H3K27ac ChIPs, hESC and hNEC input DNAs. ChIP-seq, FAIRE-seq and input libraries were prepared according to Illumina protocol and sequenced using Illumina Genome Analyser.	[36]
VDR chromatin immunoprecipitation<br>Lymphoblastoid cell lines from CEPH individuals (GM10855 and GM10861) from the International HapMap Project were used. ChIP was carried out as described in Labhart et al. [104]. Cells were cultured as described [105], all in biological duplicates, either unstimulated or stimulated for 36 h with 0.1 μM calcitriol (Sigma) and then fixed with 1% formaldehyde for 15 min and quenched with 0.125 M glycine. Chromatin was isolated by adding lysis buffer, followed by disruption with a Dounce homogenizer. Lysates were sonicated (Misonix) to **shear the DNA to an average length of 300–500 bp**. Genomic DNA (input) was purified from an aliquot of chromatin and quantified on a Nanodrop spectrophotometer. Extrapolation to the original chromatin volume allowed quantitation of the total chromatin yield.<br>ChIP assays were carried out as follows: An aliquot of chromatin (50 μg) was precleared with protein A agarose beads (Invitrogen). VDR-bound genomic DNA regions were isolated using a rabbit polyclonal antibody against VDR (Santa Cruz Biotechnology, sc-1008). After incubation at 4°C overnight, protein A agarose beads were used to isolate the immune complexes. Complexes were washed, eluted from the beads with SDS buffer, and subjected to RNase and proteinase K treatment. Crosslinks were reversed by incubation overnight at 65°C, and ChIP DNA was purified by phenol-chloroform extraction and ethanol precipitation.<br>[...]<br>ChIP sequencing (Illumina)<br>Remaining ChIP DNA (90% of entire sample) was amplified following the Illumina ChIP-seq library generation protocol. In parallel, 20ng each of input DNA (isolated from nonimmunoprecipitated chromatin) of the pooled GM10855 and GM10861 samples were also amplified for sequencing. In brief, DNA ends were polished and 5'-phosphorylated using T4 DNA polymerase, Klenow polymerase, and T4 polynucleotide kinase. After addition of 3'-A to the ends using Klenow fragment (3'-5' exo minus), Illumina genomic adapters were ligated and the sample **was size-fractionated (∼180–250 bp)** on a 2% agarose gel. After a final PCR amplification step (18 cycles, Phusion polymerase), the resulting DNA libraries were quantified and tested by QPCR at the same specific genomic regions as the original ChIP DNA to assess quality of the amplification reactions. DNA libraries were sent to Vanderbilt Microarray Shared Resource for sequencing on a Genome Analyzer II.	[37]

Continued on next page

Table S3 – *Continued from previous page*

**Protocol**	**Study Reference**
Chromatin Immunoprecipitation (ChIP). Different ChIP procedures are necessary for different applications/analyses. For example, where possible we used native ChIP to examine histone modifications, whereas fixed ChIP is needed to examine Polycomb binding and for sequential ChIP. Similarly, cChIP is used when examining ∼1,000 cells. Importantly, the same assay was always used within a figure or experiment, or when comparing cell lines. In addition, all key findings were replicated using alternative ChIP approaches where possible, confirming that the different methodologies do not affect the outcome of the experiment. In particular, the low prevalence of H3K27me3 sequence reads in TS and XEN cells was confirmed for a subset of developmentally important genes by quantitative PCR analysis of ChIP DNA. We also verified that our findings were not unique to our methodology by obtaining highly similar results using an alternative H3K27me3 antibody and by performing ChIP on fixed chromatin.<br>Native (unfixed) ChIP was performed as described [106]. Briefly, 10–50 million cells were lysed in 0.2% (vol/vol) IGEPAL CA-630 (Sigma) for 10 min on ice, nuclei were collected by centrifugation at 10,000 × g for 20 min at 4 °C and suspended in digestion buffer at 1 mg/mL. Chromatin aliquots (0.5 mg in 500 μL) were digested with 10 U micrococcal nuclease (GE Healthcare) for 6–9 min at 37 °C and soluble chromatin recovered by overnight dialysis followed by centrifugation. Successful chromatin fractionation (sample contains predominantly mononucleosomes to tetranucleosomes) was verified by agarose gel electrophoresis. Fragmented chromatin (35 μg per ChIP) was immunoprecipitated overnight at 4 °C with 2–10 μg of one of the following antibodies: H3K4me3 (Abcam, ab8580), H3K9me2 (Millipore, 07–212), H3K9me3 (Abcam, ab8898), 1 μL H3K9me3 [107], H3K27me3 (Millipore, 07–449), 1 μL H3K27me3 [108], H3K79me3 (Abcam, ab2621), H4K20me3 (Millipore, 07–463), H3K9 acetylation (Millipore, 07–352), or rabbit anti-mouse IgG (Jackson, 315–005- 003). Protein A-sepharose (100 μL of 50% (vol/vol) slurry; GE Healthcare) was added to each sample and rotated for 4 h at 4 °C. Unbound material was removed, sepharose beads washed in salt buffer (increasing sodium chloride concentration from 75 to 175 mM) and chromatin eluted from the beads with two 15-min incubations in 1% SDS at room temperature. DNA from bound, unbound, and input samples was extracted by two rounds of phenol/chloroform and precipitated with isopropanol overnight at −20 °C with 30 μg glycogen (Roche) as carrier. Air-dried DNA pellets were reconstituted in TE buffer. Throughout the procedure, all solutions were ice-cold and freshly supplemented with protease inhibitors (mini complete-EDTA free, Roche) and 5 mM sodium butyrate.<br>Formaldehyde crosslinked and sonicated chromatin was analyzed using the ChIP assay kit (Millipore) following manufacturer's protocol. Chromatin from 1 million cells was immunoprecipitated using the following antibodies: 3 μg Rnf2 (Abnova, H00006045- M01), 3 μg Ezh2 (Active Motif, 39103), 5 μg Ezh2 (Abnova, PAB0648), 5 μg H3K27me3 (Millipore, 07–449), 5 μg Eed (Mil- lipore, 09–774), and 5 μg anti-mouse IgG (Jackson 315–005- 003). [. . .] To prepare samples for Illumima sequencing, the concentration of immunoprecipitated DNA was determined using Quant-iT PicoGreen dsDNA reagent (Invitrogen) and 100 ng of each sample was sent to BC Cancer Agency Genome Sciences Centre, Vancouver, Canada, where the service was performed. Sample processing and initial raw data processing was carried out as described [91].<br>Carrier Chromatin Immunoprecipitation (cChIP). cChIP was used to analyze histone modifications in small sample sizes and performed as described [109], with minor modifications. Briefly, 50 million Drosophila melanogaster S2 cells were added to each sample of 500–2,000 target mouse cells and nuclei were isolated by incubation in 0.1% Tween 40 (vol/vol) for 1 h on ice followed by homogenization in a Dounce. Nuclei were collected by cen- trifugation, washed twice in 5% sucrose (wt/vol), and suspended in digestion buffer to a chromatin DNA concentration of 0.5 mg/ mL. Chromatin was digested with 10 U micrococcal nuclease per 50 μg chromatin for 5 min at 28 °C and soluble chromatin was recovered by overnight dialysis and centrifugation. Successful chromatin fractionation was verified by agarose gel electrophoresis. Fragmented chromatin was immunoprecipitated overnight at 4 °C with 2 μg H3K4me3 (Abcam, ab8580), 2 μg H3K27me3 (Millipore, 07–449), 1 μL H3K27me3 (7), 2 μg H3K9me3 (Ab- cam, ab8898), 1 μL H3K9me3 (6), or 2 μg anti-mouse IgG (Jackson 315–005-003). Protein A-sepharose (200 μL of 50% (vol/vol) slurry) was added to each sample and rotated for 3 h at room temperature. Unbound material was removed and kept, sepharose beads washed in salt buffer (increasing sodium chloride concentration from 50 to 150 mM), and chromatin was eluted from the beads with 2 × 15-min incubations in 1% SDS at room temperature. DNA from bound and unbound samples was extracted using the DNA purification kit (Qiagen) and eluted in 50–125 μL buffer EB. Immunoprecipitated DNA was subjected to qPCR analysis. For each sample, immunoprecipitated DNA was calculated as a ratio to antibody unbound DNA. Primers were designed to target the promoter region of each gene. Primer sequences are detailed in Table S1. [. . .] DNA (2 μg) was bisulphite modified using the Epitect bisulphite kit (Qiagen) following the manufacturer's protocol. Modified DNA (10 ng) was amplified with primers specific to bisulphite converted DNA using HotStartTaq (Qiagen) for 35 cycles, with annealing temperatures ranging from 53 °C to 56 °C. We used the universal biotinylated primer strategy, developed by Royo and colleagues, to biotin label the DNA fragment during PCR amplification [110]. Each PCR was optimized so that only one DNA fragment was generated. Quantification of DNA methylation was performed on a PSQ 96 Pyrosequencer following the manufacturer's protocol (Pyrosequencing AB) using reagents purchased from Biotage. All primers were designed using PSQ Assay Design software (Biotage) to target the promoter regions of each gene and are detailed in Table S1. Hypermethylated and hyomethylated genomic mouse DNA was used as template to confirm the absence of methylation bias within the assay (Fig. S3D).	[38]
ChIP experiments were performed with well-characterized antibodies against CTCF (Millipore, 07-729), STAG1 (Abcam, ab4457), RAD21 (Abcam, ab992), ER (Santa Cruz, sc-543), CEBPA (Santa Cruz, sc-9314), and HNF4A (Aviva Systems Biology, ARP31946) [111–114], as recently described [92]. Briefly, the immunoprecipitated material was end-repaired, A-tailed, ligated to the sequencing adapters, amplified by 18 cycles of PCR, and **size selected (200–300 bp)** followed by single end sequencing on an Illumina Genome Analyzer according to the manufacturer's recommendations.	[39]
ChIP-Seq was carried out by conventional ChIP followed by end repair of 15 ng (for protein-GFP fusions) or 30 ng (for the histone modifications) enriched DNA as measured by Qubit fluorometer using the Quant-iT dsDNA HS Assay Kit from (Invitrogen, Q32851). Adaptors were ligated to DNA fragments, which were subsequently **size selected (∼300 base pair [bp])**. The adaptor-modified DNA fragments were subjected to limited PCR amplification (14 cycles) and quality control was made by qPCR (primers sequences are available upon request), as well as by running the PCR products on a Bioanalyzer (BioRad). Finally, cluster generation and sequencing-by-synthesis (36 bp) was performed using the Illumina Genome Analyzer IIx (GAIIx) according to standard protocols of the manufacturer (Illumina).	[40]

Continued on next page

Table S3 – *Continued from previous page*

**Protocol**	**Study Reference**
Chromatin Immunoprecipitation (ChIP) and co-immunoprecipitation for transcription factors, ChIP-seq, and data analysis. Caco-2 cells were cultured in Dulbecco's Modified Eagle Medium supplemented with 10% fetal bovine serum (FBS) and 2mM L-glutamine. Proliferating cells were passaged every 2 days to maintain subconfluency; to induce maturation, cells were grown to confluence and the medium replaced every other day for an additional 26 days [115, 116]. Mouse villi were isolated as described [117] and passed through a 70uM filter (BD Falcon 352350); the material retained by the filter was harvested. For each immunoprecipitate (IP), ∼15E6 cells were cross-linked with 1% formaldehyde for 10 min at 37°C, washed in cold phosphate-buffered saline (PBS), resuspended in lysis buffer (1% SDS, 10 mM EDTA, 50mM Tris-HCl, pH 8.1, and complete protease inhibitors (Roche)), and sonicated to obtain chromatin fragments between 200 bp and 1200 bp. Sonicated chromatin was resuspended in IP buffer (1% triton, 2mM EDTA, 150 mM NaCl, 20 mM Tris-HCl, pH 8.1) and incubated overnight at 4oC with magnetic beads (Dynal) conjugated to one of the following antibodies: CDX2 (Bethyl BL3194), GATA6 c-20 (Santa Cruz 7244), GATA6 h-92 (Santa Cruz 9055), HNF4α c-19 (Santa Cruz 6547) or HNF4α h-171 (Santa Cruz 8987). The IP was washed 6 times with RIPA buffer (50 mM HEPES, pH 7.6, 1 mM EDTA, 0.7% Na deoxycholate, 1% NP-40, 0.5M LiCl) and the DNA recovered by reversing the cross-links in 1% SDS, 0.1M NaHCO3 for 8h at 65°C. DNA was purified and quantified by Picogreen (Manufacturer). 700pg of DNA was used per qPCR reaction (Applied Biosystems). [...] For ChIP-seq, 10 ng each of ChIP and input DNA from up to 3 pooled experiments were processed for deep sequencing according to manufacturer's instructions (Illumina).	[41]
For ChIP analysis of VCaP, Jurkat, HL60, SK-N-MC and CADO-ES1 cells, proteins were cross-linked to DNA by incubation of cells for 10 min in medium containing 1% formaldehyde at room temperature, after which the cross-linking was quenched by addition of glycine to 125 mM final concentration. Cells were then incubated on ice for 15 min in hypotonic lysis buffer (20 mM Tris-Cl, pH 8.0 with 10 mM KCl, 10% glycerol, 2mM DTT, and complete protease inhibitor cocktail (Sigma). Prepared nuclei were resuspended in RIPA buffer (10 mM Tris-Cl, pH 8.0 with 140 mM NaCl, 1% Triton X-100, 1% Sodium deoxycholate, 0.1% SDS, 1mM DTT, and complete protease inhibitor cocktail (Sigma)) and incubated on ice for 10 min. DNA was fragmented by sonication (16 times for 25 s each with SoniPrep 150, incubating on ice for 1 min between each pulse), and the sample cleared by centrifugation at 15000 x g for 10 min to obtain cleared chromatin lysate. The soluble lysate were diluted by addition of 5 volumes of IP buffer (20 mM Tris-Cl, pH 8.1 containing 2 mM EDTA, 150 mM NaCl and 1% Triton X-100). Subsequently, the lysate was precleared with 30 μl of protein A or G- Sepharose beads for 3 hours at 4°C under rocking. Precleared samples were incubated with 4 μg of specific antibody overnight at 4°C and the antibodies collected by incubation with 30 μl of protein A or G- Sepharose beads for 2-3 h at +4°C followed by centrifugation at 800 x g. Subsequently the beads were washed serially with wash buffers I, II, III and IV (Buffer I: 2 mM EDTA, 0.1% SDS, 1% Triton X-100, 150 mM NaCl in 20 mM Tris- Cl, pH 8.1; Buffer II: same as I but with 500 mM NaCl; Buffer III: 250 mM LiCl, 1 mM EDTA, 1% Sodium Deoxycholate and 1% NP-40 in 10 mM Tris-Cl, pH 8.1; Buffer IV 1 mM EDTA in 10 mM Tris-Cl, pH 8.1). Precipitated chromatin complexes were eluted twice with 200 μl of extraction buffer (10 mM Tris-Cl, pH 8.0 with 1 mM EDTA and 1% SDS). Subsequently, RNase A (0.2 mg/ml), Proteinase K (0.2 mg/ml), and NaCl (to 300 mM) were serially added, and the cross-links reversed by incubating overnight at 65°C. DNA was extracted with QiaQuick PCR purification kit (Qiagen) or phenol/chloroform followed by ethanol precipitation.<br>In order to induce AR activity and ERG expression in VCaP cells prior to ChIP, 70% confluent VCaP cells were treated with 1 nM of the synthetic androgen methyltrienolone (R1881, dissolved in ethanol, from Prof. Olli A. Jänne) for 24 h. [...] ChIP DNA was quantitated by PicoGreen dsDNA quantitation reagent (Molecular Probes). For preparation of the ChIP library for sequencing, 50 ng of DNA was blunted and phosphorylated with T4 DNA polymerase and T4 polynucleotide kinase at 20°C for 30 min. Subsequently, an A nucleotide was added to the 3' end of the DNA fragments using Klenow exo- (3' to 5' exo minus), followed by column purification (QIAquick, Qiagen). Illumina/Solexa amplification and sequencing adapters were ligated to the DNA-fragments and **∼120 to 350 bp fragments were size-selected** on 2% agarose gel. The fragments were enriched by 16 cycles of PCR amplification, and **size-selected again using a 2% agarose gel (to 150–300 bp)**. Purified DNA (Qiagen gel purification kit) was quantified (Nanodrop 1000 spectrophotometer) and used for massively parallel sequencing (Illumina genome analyzer) according to the manufacturer's instructions.	[42]
ChIP-Seq experiments and data processing were performed as described previously [83, 118]. Briefly, T cells (2×10^7) were treated with MNase to generate mononucleosomes fraction to analyze histone modifications using following antibodies (anti-H3K4me3, ab8580; anti-H3K36me3, ab9050, Abcam, Cambridge, MA; and anti-H3K27me3, 07-449, Millipore, Billerica MA). For STAT-ChIP, cells were chemically cross-linked and sonicated to generate fractionated genomic DNA and immunoprecipitated with anti-STAT4 (sc486) or anti-STAT6 (sc981, Santa Cruz Biotechnology Inc., Santa Cruz, CA). The DNA fragments were blunt-end ligated to the Illumina adaptors, amplified and sequenced using the Illumina Genome Analyzer II (illumina, San Diego, CA). Sequenced reads of 25 bp were obtained using the Illumina Analysis Pipeline.	[43]
MCF7 cells were grown in DMEM medium supplemented with 10% charcoal-dextran-stripped FBS for 3 days before E2 treatment. After 10 nM E2 treatment for 45 min, cells were cross-linked with 1% formaldehyde at 37°C for 10 min. ChIP assay was performed using antibodies as indicated and followed the protocols as described [119]. [...] Deep sequencing was performed using the Illumina Solexa Genome Analyzer II.	[44]
Mobilized $CD34^{+}CD133^{+}$ human hematopoietic progenitor cells (HPCs) were purified from peripheral blood lymphocytes of healthy donors using established protocols [120]. The chromatin fragments were prepared and immunoprecipitated with an anti-GABPα antibody (H180; Santa Cruz Biotechnology) or a control IgG as described previously [121]. The chromatin immunoprecipitation (ChIP) samples were amplified and sequenced using the Solexa 1G Genome Analyzer (Illumina).	[45]

Table S4: **Annotations quality using the Arpeggio data representation.A.** Davies-Bouldin indexes are shown on the left. Empirical p-values have been computed with bootstrapping (n=1000). **B.**Balanced accuracies of a KNN (k=1) classifier are shown on the right. Empirical p-values have been computed with bootstrapping (n=100).

	**A.**		**B.**		
**Annotation**	**Davies-Bouldin index**	**(p-value)**	**KNN balanced accuracy**	**Random balanced accuracy**	**(p-value)**
AB target	4.26	(0.0099)	0.31	0.08	(0.019)
Cell Line	3.97	(0.0099)	0.27	0.16	(0.019)
Organism	5.04	(0.0099)	0.56	0.50	(0.019)
Shearing	3.05	(0.0099)	0.55	0.33	(0.019)
Functional annotation	3.89	(0.0099)	0.44	0.20	(0.019)
Interaction domain	7.07	(0.015)	0.41	0.20	(0.019)
Activity annotation	5.85	(0.0099)	0.39	0.25	(0.019)
Cellular mechanism	2.61	(0.0099)	0.50	0.20	(0.019)

Table S5: **Median AUC performance of ChIP-seq data representations**

H.sapiens (n=541)

**Annotation**	**Peak overlap**	**Arpeggio**	**Arpeggio MDS**
Protein	0.58	0.54	0.52
AB target	0.72	0.59	0.59
Interaction domain	0.55	0.57	0.55
Functional annotation	0.55	0.58	0.56
Activity annotation	0.64	0.62	0.61
Cellular Mechanism	0.54	0.60	0.62
Shearing	0.58	0.58	0.57
Cell Line	0.70	0.63	0.64
Study ID	0.70	0.63	0.64

M.musculus (n=237)

**Annotation**	**Peak overlap**	**Arpeggio**	**Arpeggio MDS**
Protein	0.77	0.65	0.60
AB target	0.86	0.64	0.59
Interaction domain	0.58	0.55	0.58
Functional annotation	0.59	0.57	0.58
Activity annotation	0.68	0.61	0.59
Cellular Mechanism	0.60	0.61	0.65
Shearing	0.49	0.50	0.49
Cell Line	0.61	0.56	0.55
Study ID	0.58	0.59	0.58

Supplementary Figures

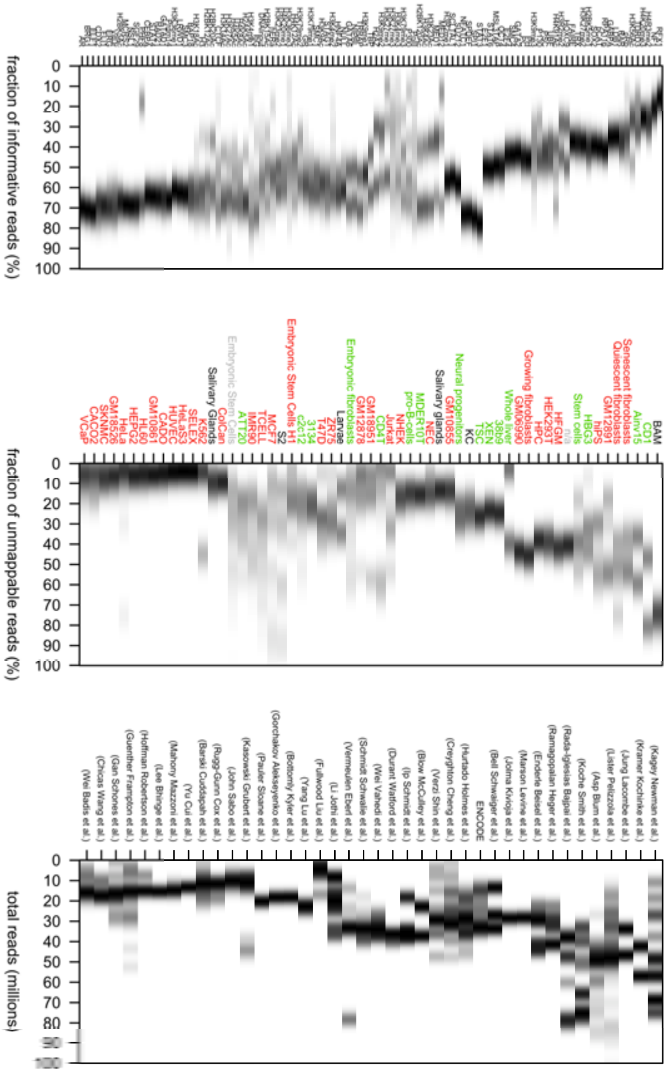

Figure S1: The different experiments exhibited large heterogeneity in terms of total number of reads, fraction of umappable reads, and fraction of flattened aligned reads. In the central panel, cell names are colored according to the organism: black for fruit fly, red for human and green for mouse.

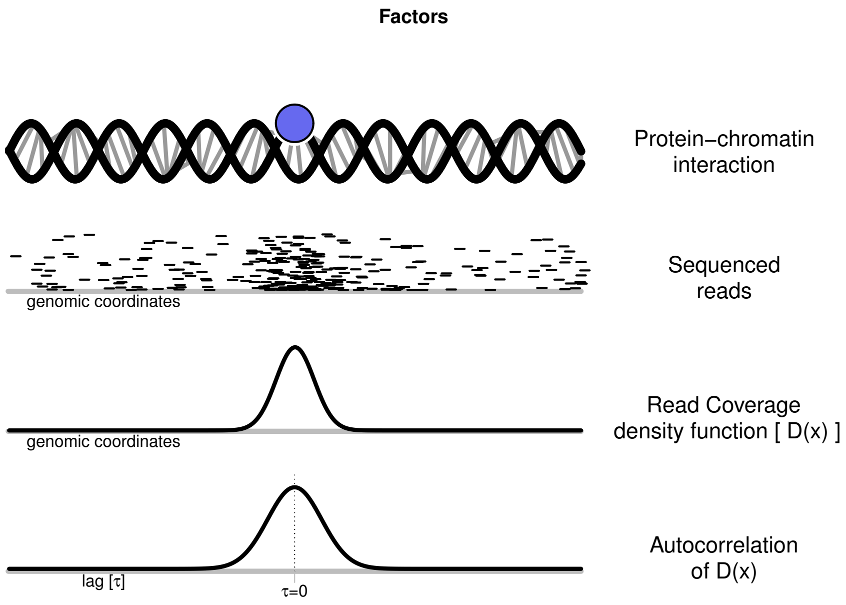

Figure S2: Illustrative cartoon of the autocorrelation function expected from the interaction between chromatin and a factor. The localized interaction between a factor and the chromatin results in isolated pulses. The corresponding autocorrelation function is also a pulse centered at lag $\tau = 0$.

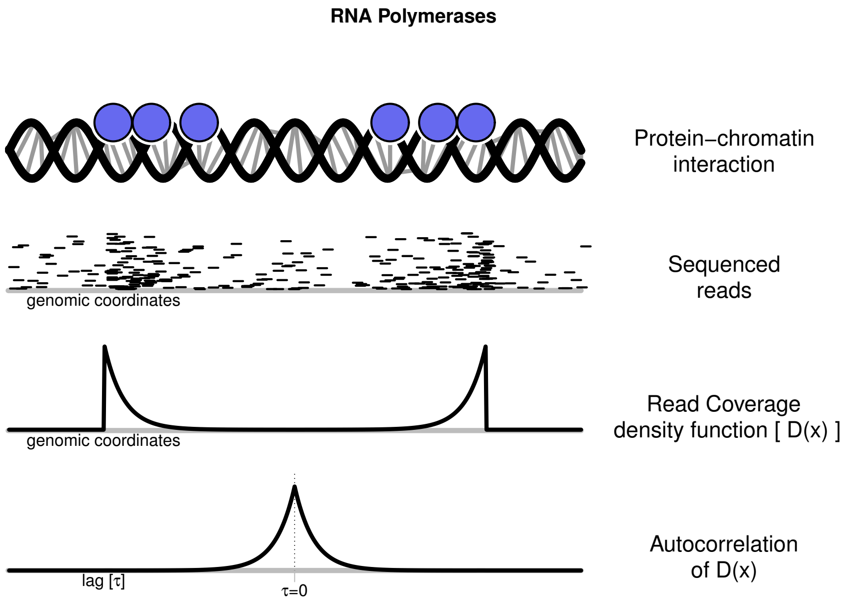

Figure S3: Illustrative cartoon of the autocorrelation function expected from the interaction between chromatin and polymerases. The delocalized interaction between chromatin and polymerase complexes corresponds to decaying functions, with a maximum near the transcription start site where the polymerase sits before initiating transcription. In the figure, two genes on opposite strands are being transcribed by three polymerases each. The corresponding autocorrelation function is a decaying function. Symmetry in the autocorrelation is due to averaging between gene bodies on opposite strands.

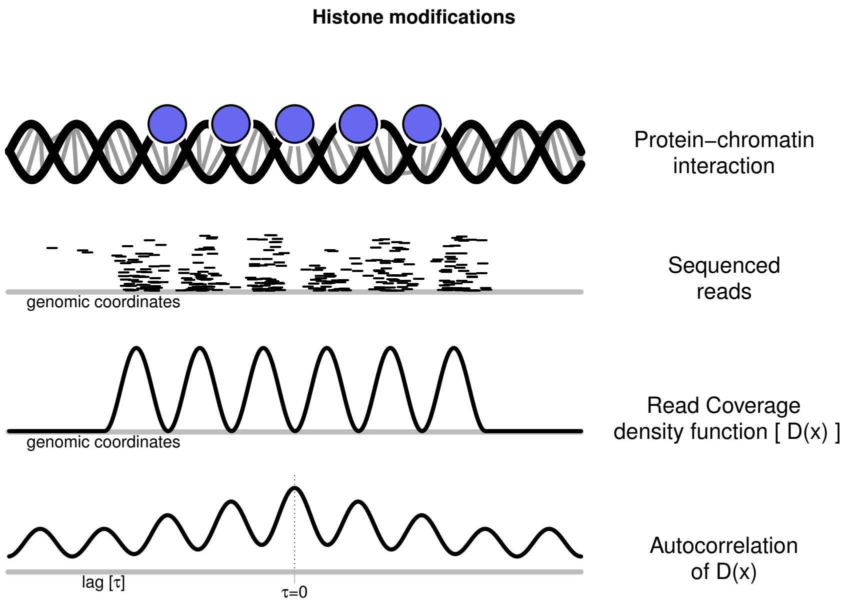

Figure S4: Illustrative cartoon of the autocorrelation function expected from the interaction between chromatin and nucleosomes carrying a specific histone modification. For simplicity of representation, each blue circle indicates the region of DNA protected by the presence of a nucleosome. The regularity in the spacing between consecutive nucleosomes determines periodicity in the read coverage density function. As a consequence, the autocorrelation is a periodic function, with period equal to the inter-nucleosomal distance.

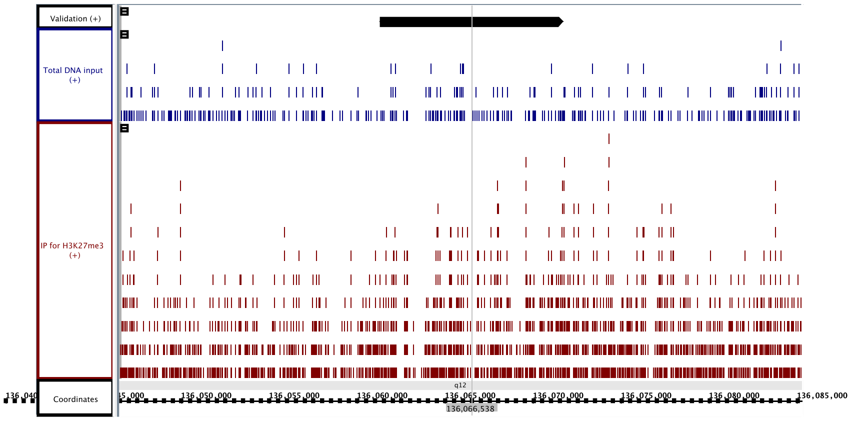

Figure S5: **Illustrative example of noise and undersampling in ChIP-seq data.** Aligned reads for a histone 3 Lysine 27 tri-methylation (H3K27me3) murine sample are shown (in red) together with reads from the best matching total DNA input control (in blue) in a region around a PCR validated H3K27me3 site (in black). Sequencing data was from [4]. Validation data was obtained from [66]. The figure was generated using the IgV genome browser [122].

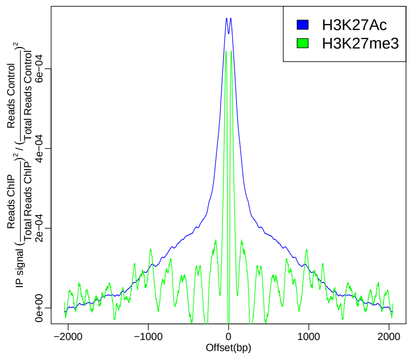

Figure S6: Examples of Arpeggio profiles with poorly matched controls. The difference in read count and distribution shape gives rise to the inverted center peak. The value representing the total read count difference between ChIP and control at $\tau = 0$ was removed and smoothing was applied to aid in visualization.

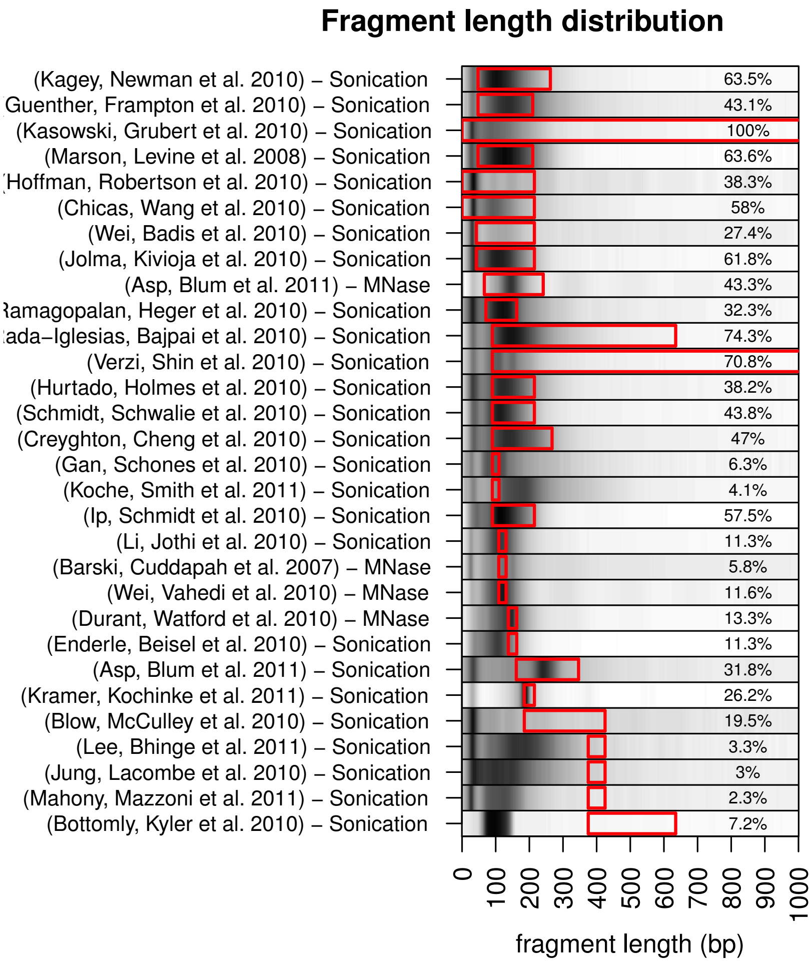

Figure S7: Comparison between reported fragment sizes grouped by study, where available, and the distribution of expected fragment sizes estimated using the Arpeggio technique. For each experiment the inferred fragment size distribution is shown as a heatmap (in greyscale), where darker colors correspond to a higher probability of a specific fragment size. The reported excision band is shown as a red rectangle, extended by 5% in both directions. The weight enclosed within the excision rectangle is shown as a percentage relative to the weight of the distribution between 0bp and 2kbp.

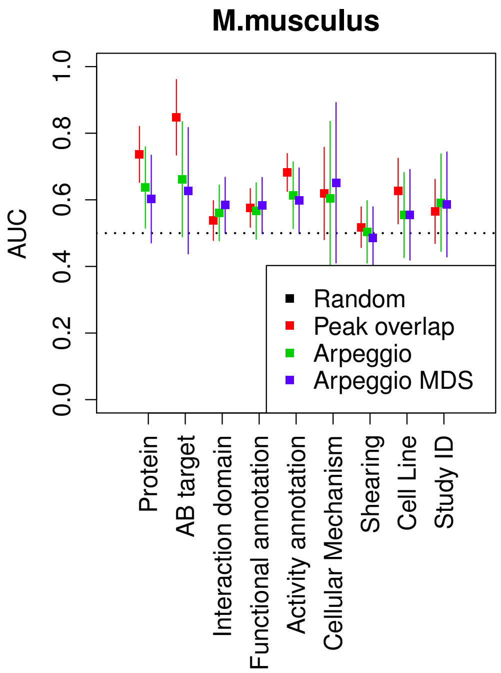

Figure S8: Classificatin of biological and experimental factors in terms of peak-based and Arpeggio-based ChIP-seq signatures for murine samples (see Figure 5). Proximity based on Arpeggio MDS had a higher median performance of predicting cellular mechanism.

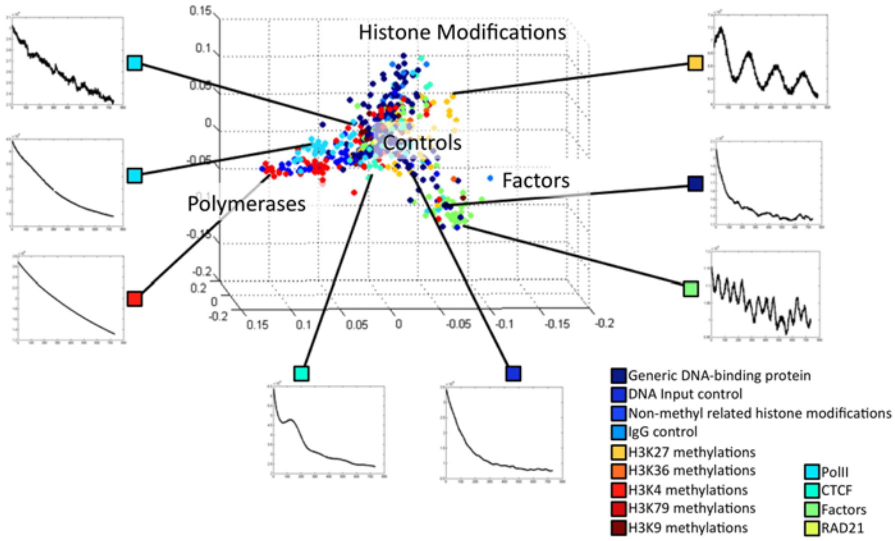

Figure S9: **Low-dimensional embedding of ChIP-seq autocorrelations using the first three diffusion coordinates.** The low-dimensional mapping has a three-branch structure with a common origin. In the insets, the autocorrelation functions in the range $0 \leq \tau \leq 1000$bp are shown for selected samples as illustrative examples.

Supplementary References

[68] Chen, Y., Negre, N., Li, Q., Mieczkowska, J.O., Slattery, M., Liu, T., Zhang, Y., Kim, T.K., He, H.H., Zieba, J. *et al.* (2012) Systematic evaluation of factors influencing ChIP-seq fidelity. *Nat. Methods*, **9**, 609–614.

[69] Chung, F.R.K. (1997) Spectral Graph Theory. Amer Mathematical Society. Providence, USA.

[70] Coifman, R.R., Lafon, S., Lee, A.B., Maggioni, M., Nadler, B., Warner, F. and Zucker, S.W. (2005) Geometric diffusions as a tool for harmonic analysis and structure definition of data: diffusion maps. *Proc. Natl. Acad. Sci. U.S.A.*, **102**, 7426–7431.

[71] Coifman, R.R. and Lafon, S. (2006) Diffusion maps. *Appl. Comput. Harmon. Anal.*, **21**, 5–30.

[72] Nadler, B., Lafon, S., Coifman, R.R. and Kevrekidis, I.G. (2006) Diffusion maps, spectral clustering and reaction coordinates of dynamical systems. *Appl. Comput. Harmon. Anal.*, **21**, 113–127.

[73] Maimon, O. (2009) *Soft computing for knowledge discovery and data mining.* Springer-Verlag. New York. USA

[74] Blais, A., van Oevelen, C.J., Margueron, R., Acosta-Alvear, D. and Dynlacht, B.D. (2007) Retinoblastoma tumor suppressor protein-dependent methylation of histone H3 lysine 27 is associated with irreversible cell cycle exit. *J. Cell. Biol.*, **179**, 1399–1412.

[75] Heintzman, N.D., Stuart, R.K., Hon, G., Fu, Y., Ching, C.W., Hawkins, R.D., Barrera, L.O., Van Calcar, S., Qu, C., Ching, K.A. *et al.* (2007) Distinct and predictive chromatin signatures of transcriptional promoters and enhancers in the human genome. *Nat. Genet.*, **39**, 311–318.

[76] Welboren, W.-J., van Driel, M.A., Janssen-Megens, E.M., van Heeringen, S.J., Sweep, F.C.G.J., Span, P.N. and Stunnenberg, H.G. (2009) ChIP-Seq of ERα and RNA polymerase II defines genes differentially responding to ligands. *EMBO J.*, **28**, 1418–1428.

[77] Schwaiger, M., Stadler, M.B., Bell, O., Kohler, H., Oakeley, E.J. and Schubeler, D. (2009) Chromatin state marks cell-type- and gender-specific replication of the Drosophila genome. *Genes Dev.*, **23**, 589–601.

[78] Visel, A., Blow, M.J., Li, Z., Zhang, T., Akiyama, J.A., Holt, A., Plajzer-Frick, I., Shoukry, M., Wright, C., Chen, F. *et al.* (2009) ChIP-seq accurately predicts tissue-specific activity of enhancers. *Nature*, **457**, 854–858.

[79] Barrera, L.O., Li, Z., Smith, A.D., Arden, K.C., Cavenee, W.K., Zhang, M.Q., Green, R.D. and Ren, B. (2007) Genome-wide mapping and analysis of active promoters in mouse embryonic stem cells and adult organs. *Genome Res.*, **18**, 46–59.

[80] Li, Z., Van Calcar, S., Qu, C., Cavenee, W.K., Zhang, M.Q. and Ren, B. (2003) A global transcriptional regulatory role for c-Myc in Burkitt's lymphoma cells. *Proc. Natl. Acad. Sci. U.S.A.*, **100**, 8164–8169.

[81] Blow, M.J., Zhang, T., Woyke, T., Speller, C.F., Krivoshapkin, A., Yang, D.Y., Derevianko, A. and Rubin, E.M. (2008) Identification of ancient remains through genomic sequencing. *Genome Res.*, **18**, 1347–1353.

[82] Yochum, G.S., Cleland, R., McWeeney, S. and Goodman, R.H. (2006) An Antisense Transcript Induced by Wnt/β-Catenin Signaling Decreases E2F4. *J. Biol. Chem.*, **282**, 871–878.

[83] Wei, G., Wei, L., Zhu, J., Zang, C., Hu-Li, J., Yao, Z., Cui, K., Kanno, Y., Roh, T.-Y., Watford, W.T. *et al.* (2009) Global Mapping of H3K4me3 and H3K27me3 Reveals Specificity and Plasticity in Lineage Fate Determination of Differentiating CD4+ T Cells. *Immunity*, **30**, 155–167.

[84] Orlando, V., Strutt, H. and Paro, R. (1997) Analysis of Chromatin Structure by in Vivo Formaldehyde Cross-Linking. *Methods*, **11**, 205–214.

[85] Strutt, H. and Paro, R. (1997) The polycomb group protein complex of Drosophila melanogaster has different compositions at different target genes. *Mol. Cell. Biol.*, **17**, 6773.

[86] Beisel, C., Buness, A., Roustan-Espinosa, I.M., Koch, B., Schmitt, S., Haas, S.A., Hild, M., Katsuyama, T. and Paro, R. (2007) Comparing active and repressed expression states of genes controlled by the Polycomb/Trithorax group proteins. *Proc. Natl. Acad. Sci. U.S.A.*, **104**, 16615–16620.

[87] Larschan, E., Alekseyenko, A.A., Gortchakov, A.A., Peng, S., Li, B., Yang, P., Workman, J.L., Park, P.J. and Kuroda, M.I. (2007) MSL Complex Is Attracted to Genes Marked by H3K36 Trimethylation Using a Sequence-Independent Mechanism. *Mol. Cell*, **28**, 121–133.

[88] Alekseyenko, A.A., Peng, S., Larschan, E., Gorchakov, A.A., Lee, O.-K., Kharchenko, P., McGrath, S.D., Wang, C.I., Mardis, E.R., Park, P.J. *et al.* (2008) A Sequence Motif within Chromatin Entry Sites Directs MSL Establishment on the Drosophila X Chromosome. *Cell*, **134**, 599–609.

[89] Lee, T.I., Johnstone, S.E. and Young, R.A. (2006) Chromatin immunoprecipitation and microarray-based analysis of protein location. *Nat. Protoc.*, **1**, 729–748.

[90] Wederell, E.D., Bilenky, M., Cullum, R., Thiessen, N., Dagpinar, M., Delaney, A., Varhol, R., Zhao, Y., Zeng, T., Bernier, B. *et al.* (2008) Global analysis of in vivo Foxa2-binding sites in mouse adult liver using massively parallel sequencing. *Nucleic Acids Res.*, **36**, 4549–4564.

[91] Robertson, G., Hirst, M., Bainbridge, M., Bilenky, M., Zhao, Y., Zeng, T., Euskirchen, G., Bernier, B., Varhol, R., Delaney, A. *et al.* (2007) Genome-wide profiles of STAT1 DNA association using chromatin immunoprecipitation and massively parallel sequencing. *Nat. Met.*, **4**, 651–657.

[92] Schmidt, D., Wilson, M.D., Spyrou, C., Brown, G.D., Hadfield, J. and Odom, D.T. (2009) ChIP-seq: Using high-throughput sequencing to discover protein-DNA interactions. *Methods*, **48**, 240–248.

[93] John, S., Sabo, P.J., Johnson, T.A., Sung, M.-H., Biddie, S.C., Lightman, S.L., Voss, T.C., Davis, S.R., Meltzer, P.S., Stamatoyannopoulos, J.A. *et al.* (2008) Interaction of the Glucocorticoid Receptor with the Chromatin Landscape. *Mol. Cell*, **29**, 611–624.

[94] Tuupanen, S., Turunen, M., Lehtonen, R., Hallikas, O., Vanharanta, S., Kivioja, T., Björklund, M., Wei, G., Yan, J., Niittymäki, I. *et al.* (2009) The common colorectal cancer predisposition SNP rs6983267 at chromosome 8q24 confers potential to enhanced Wnt signaling. *Nat. Genet.*, **41**, 885–890.

[95] Wichterle, H., Lieberam, I., Porter, J.A. and Jessell, T.M. (2002) Directed Differentiation of Embryonic Stem Cells into Motor Neurons. *Cell*, **110**, 385–397.

[96] Boyer, L.A., Plath, K., Zeitlinger, J., Brambrink, T., Medeiros, L.A., Lee, T.I., Levine, S.S., Wernig, M., Tajonar, A., Ray, M.K. *et al.* (2006) Polycomb complexes repress developmental regulators in murine embryonic stem cells. *Nature*, **441**, 349–353.

[97] Euskirchen, G.M., Rozowsky, J.S., Wei, C.L., Lee, W.H., Zhang, Z.D., Hartman, S., Emanuelsson, O., Stolc, V., Weissman, S., Gerstein, M.B. *et al.* (2007) Mapping of transcription factor binding regions in mammalian cells by ChIP: Comparison of array- and sequencing-based technologies. *Genome Res.*, **17**, 898–909.

[98] Rozowsky, J., Euskirchen, G., Auerbach, R.K., Zhang, Z.D., Gibson, T., Bjornson, R., Carriero, N., Snyder, M. and Gerstein, M.B. (2009) PeakSeq enables systematic scoring of ChIP-seq experiments relative to controls. *Nat. Biotechnol.*, **27**, 66–75.

[99] Auerbach, R.K., Euskirchen, G., Rozowsky, J., Lamarre-Vincent, N., Moqtaderi, Z., Lefrancois, P., Struhl, K., Gerstein, M. and Snyder, M. (2009) Mapping accessible chromatin regions using Sono-Seq. *Proc. Natl. Acad. Sci. U.S.A.*, **106**, 14926–14931.

[100] Kim, J., Lee, J.-H. and Iyer, V.R. (2008) Global Identification of Myc Target Genes Reveals Its Direct Role in Mitochondrial Biogenesis and Its E-Box Usage In Vivo. *PLoS ONE*, **3**, e1798.

[101] Guenther, M.G., Lawton, L.N., Rozovskaia, T., Frampton, G.M., Levine, S.S., Volkert, T.L., Croce, C.M., Nakamura, T., Canaani, E. and Young, R.A. (2008) Aberrant chromatin at genes encoding stem cell regulators in human mixed-lineage leukemia. *Genes Dev.*, **22**, 3403–3408.

[102] Regha, K., Sloane, M.A., Huang, R., Pauler, F.M., Warczok, K.E., Melikant, B., Radolf, M., Martens, J.H.A., Schotta, G., Jenuwein, T. *et al.* (2007) Active and Repressive Chromatin Are Interspersed without Spreading in an Imprinted Gene Cluster in the Mammalian Genome. *Mol. Cell*, **27**, 353–366.

[103] Boyer, L.A., Lee, T.I., Cole, M.F., Johnstone, S.E., Levine, S.S., Zucker, J.P., Guenther, M.G., Kumar, R.M., Murray, H.L., Jenner, R.G. *et al.* (2005) Core Transcriptional Regulatory Circuitry in Human Embryonic Stem Cells. *Cell*, **122**, 947–956.

[104] Labhart, P. (2005) Identification of target genes in breast cancer cells directly regulated by the SRC-3/AIB1 coactivator. *Proc. Natl. Acad. Sci. U.S.A.*, **102**, 1339–1344.

[105] Ramagopalan, S.V., Maugeri, N.J., Handunnetthi, L., Lincoln, M.R., Orton, S.-M., Dyment, D.A., DeLuca, G.C., Herrera, B.M., Chao, M.J., Sadovnick, A.D. *et al.* (2009) Expression of the Multiple Sclerosis-Associated MHC Class II Allele HLA-DRB1*1501 Is Regulated by Vitamin D. *PLoS Genetics*, **5**, e1000369.

[106] Umlauf, D., Goto, Y. and Feil, R. (2004) Site-specific analysis of histone methylation and acetylation. *Methods Mol. Biol.*, **287**, 99–120.

[107] Cowell, I., Aucott, R., Mahadevaiah, S., Burgoyne, P., Huskisson, N., Bongiorni, S., Prantera, G., Fanti, L., Pimpinelli, S., Wu, R. *et al.* (2002) Heterochromatin, HP1 and methylation at lysine 9 of histone H3 in animals. *Chromosoma*, bf 111, 22–36.

[108] Peters, A.H.F.M., Kubicek, S., Mechtler, K., O'Sullivan, R.J., Derijck, A.A.H.A., Perez-Burgos, L., Kohlmaier, A., Opravil, S., Tachibana, M., Shinkai, Y. *et al.* (2003) Partitioning and Plasticity of Repressive Histone Methylation States in Mammalian Chromatin. *Mol. Cell*, **12**, 1577–1589.

[109] O'Neill, L.P., VerMilyea, M.D. and Turner, B.M. (2006) Epigenetic characterization of the early embryo with a chromatin immunoprecipitation protocol applicable to small cell populations. *Nat. Genet.*, **38**, 835–841.

[110] Royo, J.L., Hidalgo, M. and Ruiz, A. (2007) Pyrosequencing protocol using a universal biotinylated primer for mutation detection and SNP genotyping. *Nat. Protoc.*, **2**, 1734–1739.

[111] Carroll, J.S., Meyer, C.A., Song, J., Li, W., Geistlinger, T.R., Eeckhoute, J., Brodsky, A.S., Keeton, E.K., Fertuck, K.C., Hall, G.F. *et al.* (2006) Genome-wide analysis of estrogen receptor binding sites. *Nat. Genet.*, **38**, 1289–1297.

[112] Kim, T.H., Abdullaev, Z.K., Smith, A.D., Ching, K.A., Loukinov, D.I., Green, Roland D., Zhang, M.Q., Lobanenkov, V.V. and Ren, B. (2007) Analysis of the Vertebrate Insulator Protein CTCF-Binding Sites in the Human Genome. *Cell*, **128**, 1231–1245.

[113] Lefterova, M.I., Zhang, Y., Steger, D.J., Schupp, M., Schug, J., Cristancho, A., Feng, D., Zhuo, D., Stoeckert, C.J., Liu, X.S. *et al.* (2008) PPAR and C/EBP factors orchestrate adipocyte biology via adjacent binding on a genome-wide scale. *Genes Dev.*, **22**, 2941–2952.

[114] Wendt, K.S., Yoshida, K., Itoh, T., Bando, M., Koch, B., Schirghuber, E., Tsutsumi, S., Nagae, G., Ishihara, K., Mishiro, T. *et al.* (2008) Cohesin mediates transcriptional insulation by CCCTC-binding factor. *Nature*, **451**, 796–801.

[115] Halbleib, J.M., Sääf, A.M., Brown, P.O. and Nelson, W.J. (2007) Transcriptional Modulation of Genes Encoding Structural Characteristics of Differentiating Enterocytes During Development of a Polarized Epithelium In Vitro. *Mol. Biol. Cell*, **18**, 4261–4278.

[116] Sääf, A.M., Halbleib, J.M., Chen, X., Yuen, S.T., Leung, S.Y., Nelson, W.J. and Brown, P.O. (2007) Parallels between Global Transcriptional Programs of Polarizing Caco-2 Intestinal Epithelial Cells In Vitro and Gene Expression Programs in Normal Colon and Colon Cancer. *Mol. Biol. Cell*, **18**, 4245–4260.

[117] Guo, J., Longshore, S., Nair, R. and Warner, B.W. (2008) Retinoblastoma Protein (pRb), but Not p107 or p130, Is Required for Maintenance of Enterocyte Quiescence and Differentiation in Small Intestine. *J. Biol. Chem.*, **284**, 134–140.

[118] Zang, C., Schones, D.E., Zeng, C., Cui, K., Zhao, K. and Peng, W. (2009) A clustering approach for identification of enriched domains from histone modification ChIP-Seq data. *Bioinformatics*, **25**, 1952–1958.

[119] Iberg, A.N., Espejo, A., Cheng, D., Kim, D., Michaud-Levesque, J., Richard, S. and Bedford, M.T. (2007) Arginine Methylation of the Histone H3 Tail Impedes Effector Binding. *J. Biol. Chem.*, **283**, 3006–3010.

[120] Migliaccio, G., Di Pietro, R., di Giacomo, V., Di Baldassarre, A., Migliaccio, A.R., Maccioni, L., Galanello, R. and Papayannopoulou, T. (2002) In Vitro Mass Production of Human Erythroid Cells from the Blood of Normal Donors and of Thalassemic Patients. *Blood Cells, Molecules, and Diseases*, **28**, 169–180.

[121] Cui, K., Zang, C., Roh, T.-Y., Schones, D.E., Childs, R.W., Peng, W. and Zhao, K. (2009) Chromatin Signatures in Multipotent Human Hematopoietic Stem Cells Indicate the Fate of Bivalent Genes during Differentiation. *Cell Stem Cell*, **4**, 80–93.

[122] Thorvaldsdóttir, H., Robinson, J.T. and Mesirov, J.P. (2013) Integrative Genomics Viewer (IGV): high-performance genomics data visualization and exploration. *Brief. Bioinform.*, **14**, 178–192.
